# A β-barrel-like tetramer formed by a β-hairpin derived from Aβ†

**DOI:** 10.1039/d3sc05185d

**Published:** 2023-11-28

**Authors:** Tuan D. Samdin, Chelsea R. Jones, Gretchen Guaglianone, Adam G. Kreutzer, J. Alfredo Freites, Michał Wierzbicki, James S. Nowick

**Affiliations:** a Department of Chemistry, University of California Irvine California 92697-2025 USA jsnowick@uci.edu; b Department of Pharmaceutical Sciences, University of California, Irvine Irvine California 92697-2025 USA

## Abstract

β-Hairpins formed by the β-amyloid peptide Aβ are building blocks of Aβ oligomers. Three different alignments of β-hairpins have been observed in the structures of Aβ oligomers or fibrils. Differences in β-hairpin alignment likely contribute to the heterogeneity of Aβ oligomers and thus impede their study at high-resolution. Here, we designed, synthesized, and studied a series of β-hairpin peptides derived from Aβ_12–40_ in one of these three alignments and investigated their solution-phase assembly and folding. These assays reveal the formation of tetramers and octamers that are stabilized by intermolecular hydrogen bonding interactions between Aβ residues 12–14 and 38–40 as part of an extended β-hairpin conformation. X-ray crystallographic studies of one peptide from this series reveal the formation of β-barrel-like tetramers and octamers that are stabilized by edge-to-edge hydrogen bonding and hydrophobic packing. Dye-leakage and caspase 3/7 activation assays using tetramer and octamer forming peptides from this series reveal membrane-damaging and apoptotic properties. A molecular dynamics simulation of the β-barrel-like tetramer embedded in a lipid bilayer shows membrane disruption and water permeation. The tetramers and octamers described herein provide additional models of how Aβ may assemble into oligomers and supports the hypothesis that β-hairpin alignment and topology may contribute directly to oligomer heterogeneity.

## Abstract 2

β-Hairpins formed by the β-amyloid peptide Aβ are building blocks of Aβ oligomers.

## Graphical abstract

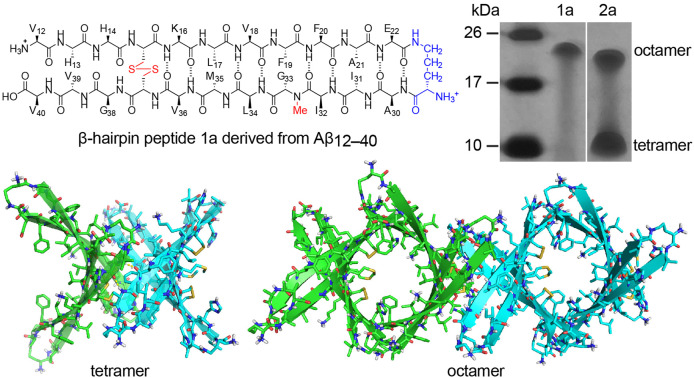

## Introduction

The formation and biological activity of β-amyloid oligomers are central events in the pathogenesis and progression of Alzheimer's disease. Dimers of Aβ isolated from Alzheimer's brains have been shown to disrupt neuritic integrity *in vitro*, and in a separate study impair synaptic structure and function *in vivo*.^1,2^ Synthetic trimers of peptides derived from Aβ have been used to raise antibodies that recognize pathological features in tissues isolated from Alzheimer's brains.^3^ Tetramers and octamers of Aβ have demonstrated pore-forming activity against lipid-bilayers.^4^ Photo-induced crosslinking studies of Aβ_42_ have revealed the formation of pentamers and hexamers, while ion mobility-mass spectrometry studies have shown that Aβ_42_ forms β-barrel shaped hexamers in the presence of membrane mimetic micelles.^5,6^ Understanding the biophysical and biological activity of Aβ oligomers is key to understanding the molecular basis of neurodegeneration and cognitive decline in Alzheimer's disease.

Aβ oligomers, however, are unstable and exhibit significant variation in their stoichiometry and structure—variation that is reflected by the dizzying alphabet soup of terms used to describe them, including ADDLs (Aβ-derived diffusible ligands), AβOs (Aβ oligomers), PFs (protofibrils), PFOs (prefibrillar oligomers), and APFs (annular protofibrils).^7–11^ Structure–activity relationship studies are often frustrated by the heterogeneous and polydisperse nature of Aβ assemblies, as it equilibrates between monomeric, oligomeric, and fibrillar species.^12–16^ To overcome these difficulties fragment-based investigations, covalent-stabilization, synthetic Aβ oligomer homologues, and *in silico* modeling have emerged as tools to aid in the study of more uniform and homogenous models of Aβ oligomers.^17^ A substantial body of evidence has emerged from these investigations, identifying β-hairpins as a key structural element of Aβ oligomers.

The NMR-based structure of a tetramer formed by full-length Aβ_42_ reported by Carulla and coworkers is the only atomic-resolution structure of an Aβ oligomer that has been deposited in the Protein Data Bank, (PDB 6RHY).^4^ The tetramer comprises a six-stranded antiparallel β-sheet, with two β-hairpins of Aβ_42_ that flank two antiparallel β-strands of Aβ_42_ (ESI Fig. S1a†). Additional solution-phase studies of this tetramer also provide evidence for the formation of an octamer. Using molecular dynamics (MD) simulations, Carulla and coworkers propose a model in which this tetramer, as well as the octamer, can act to disrupt a lipid membrane and facilitate water permeation. Collectively, these studies have brought into sharp relief the importance of β-hairpins in the structures of Aβ oligomers.

Several additional studies have also established the significance of β-hairpins in Aβ oligomers.^18–22^ In 2008, Härd and Hoyer reported the NMR structure of a monomer of Aβ_40_ adopting a β-hairpin conformation when sequestered and stabilized by an affibody.^19^ Härd and coworkers subsequently stabilized this Aβ β-hairpin using an intermolecular disulfide-bridge and found that the stabilized β-hairpin formed oligomers that mimicked some of the properties of oligomers formed by unmodified Aβ.^20^ NMR spectroscopic studies revealed that related disulfide-stabilized β-hairpins derived from Aβ_16–42_ assembled to form a barrel-shaped hexamer stabilized by hydrophobic packing and edge-to-edge hydrogen bonding between β-hairpins (ESI Fig. S1b†).^21^

**Fig. 1:**
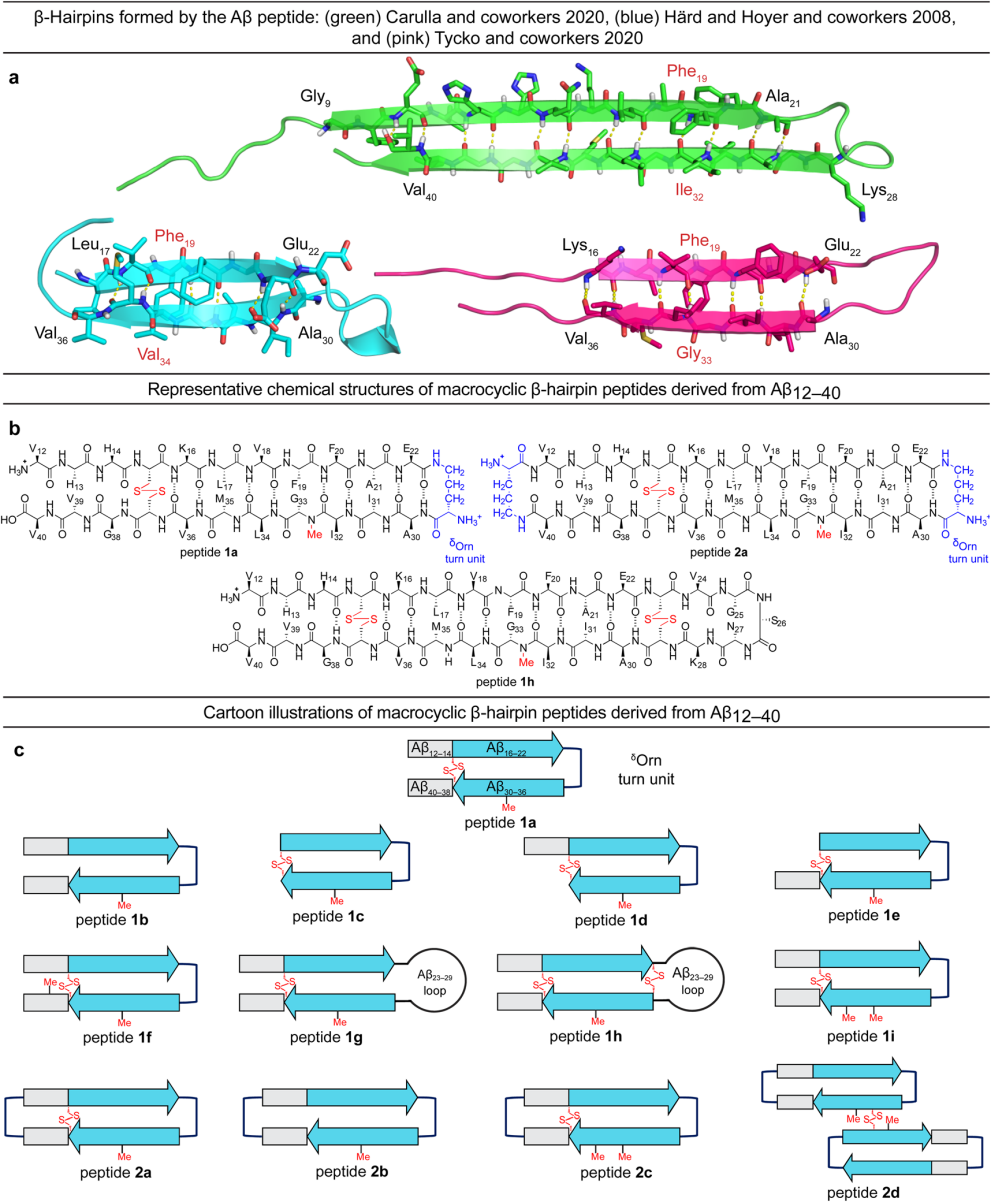
(a) Structures of Aβ β-hairpins reported by Carulla and coworkers (PDB 6RHY), Härd and Hoyer and coworkers (PDB 2OTK), and Tycko and coworkers (not deposited). (b and c) Chemical structures and cartoons of β-hairpin peptides derived from Aβ_12–40_.

Tycko and coworkers recently reported a β-hairpin as a component in the structure of an atypical Aβ_40_ fibril structure, in which the characteristic core of parallel in-register β-sheets is coated by an outer layer of β-hairpins formed by Aβ (ESI Fig. S1c†).^23^ A model of the β-hairpin that fits the cryo-EM and NMR spectroscopic data shows residues 16–22 and 30–36 hydrogen bonding to form a β-sheet, with the intervening residues 23–29 forming a loop (Fig. 1a).

The Aβ β-hairpins reported by Carulla and coworkers, Härd and Hoyer and coworkers, and Tycko and coworkers all differ in the alignment of their β-strands (Fig. 1a).^4,19,23^ In the tetramer reported by Carulla and coworkers, β-strands comprising residues 9–21 and 28–40 hydrogen bond to form an antiparallel β-sheet, with residues 22–27 forming a loop. In the barrel-shaped hexamer reported Härd and coworkers, β-strands comprising residues 17–22 and 30–36 hydrogen bond to form an antiparallel β-sheet, with residues 23–29 forming a loop. These differences in β-strand alignment alter the overall topology of the β-hairpins by shifting residue pairings across the β-strands, the hydrophobicity of the β-hairpin surfaces, and the size of the loop segments between the β-strands. In the β-hairpin reported by Carulla and coworkers, Ile_32_ is across from Phe_19_; in the β-hairpin reported by Tycko and coworkers, Gly_33_ is across from Phe_19_; and in the β-hairpin reported by Härd and Hoyer and coworkers, Val_34_ is across from Phe_19_ (Fig. 1a). These changes in alignment and topology may contribute to the immense variation and heterogeneity observed in the assembly and structures of Aβ fibrils and oligomers.

In the current study, we set out to explore oligomers formed by β-hairpins in the alignment reported by Tycko and coworkers by designing, synthesizing, and studying a series of β-hairpin peptides derived from Aβ_12–40_.^17,24^ The structures of these peptides are illustrated in Fig. 1b and c. Four of these peptides (1a, 1d, 1h, and 2a) assemble to form octamers in SDS-PAGE. X-ray crystallographic studies of peptide 2a reveal a hitherto unprecedented β-barrel-like tetramer composed of β-hairpins. The crystallographic tetramers concatenate within the crystal lattice to create an octamer, and thus suggest a structural model for the octamers observed in SDS-PAGE.

## Results and discussion

### Design of peptides 1a–i and 2a–d

We designed peptide 1a to fold into an Aβ β-hairpin in the alignment reported by Tycko and coworkers to probe the assembly of Aβ β-hairpins into oligomers. Peptide 1a comprises two peptide β-strands of Aβ_12–22_ and Aβ_30–40_ linked by a ^δ^Orn turn unit connecting residues 22 and 30, a cross-strand disulfide bridge replacing Gln_15_ and Gly_37_, and an *N*-methyl group on Gly_33_ to prevent uncontrolled aggregation.^22,25–33^ We designed homologous peptides 1b–i and 2a–d to further explore the effects of the N- and C-terminal residues 12–14 and 38–40, the Aβ_23–29_ loop, and additional ^δ^Orn turn units and disulfide bridges on folding and assembly.

### Oligomerization of peptides 1a–h and 2a–d

**Fig. 2:**
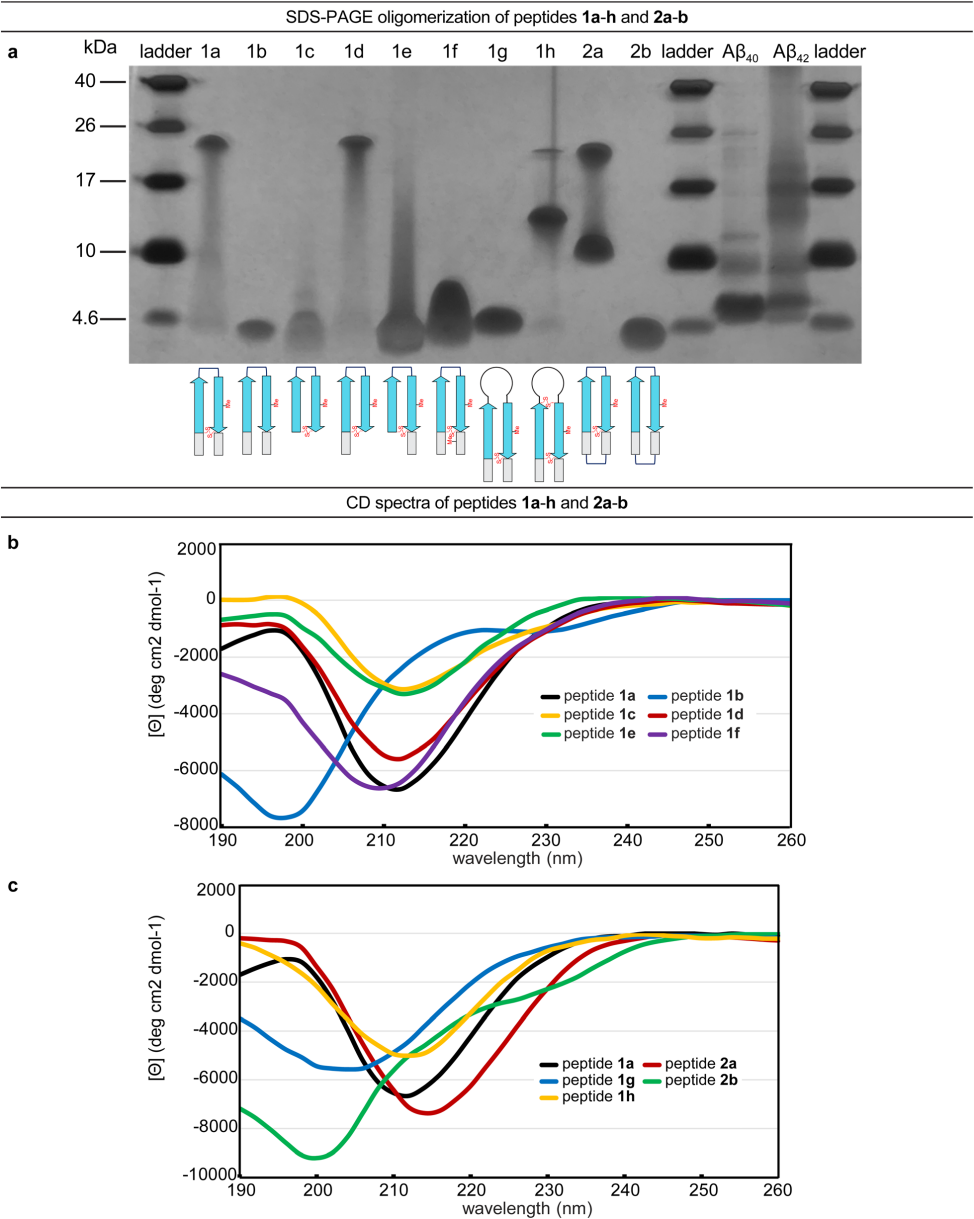
(a) Silver stained SDS-PAGE of peptides 1a–h, 2a–b, and Aβ_40_ and Aβ_42_. SDS-PAGE was performed in Tris buffer at pH 6.8 with 2% (w/v) SDS on a 16% polyacrylamide gel with 50 µM solutions of peptide in each lane. (b and c) Circular dichroism (CD) spectra of peptides 1a–h and 2a–b. CD spectra were acquired for each peptide at 50 µM in 10 mM phosphate buffer at pH 7.4; ellipticity was normalized for the number of residues in each peptide. Additional CD spectra for peptides 1i, 2c, and 2d are provided in the (ESI Fig. S2†).

The formation of oligomers that can be observed in SDS-PAGE is a hallmark of Aβ.^7,34–37^ Peptide 1a (2.5 kDa) runs as an oligomer in SDS-PAGE, migrating at a molecular weight consistent with that of an octamer (*ca.* 20 kDa; Fig. 2a). The octamer band streaks downward from below the 26 kDa ladder band, suggesting that the octamer is in equilibrium with lower molecular weight species. Unlike the oligomer formed by peptide 1a, the oligomers formed by full-length, unmodified Aβ are heterogeneous in size and typically display a substantial band for the monomer (Fig. 2a).^5,34,36^ The formation of a well-defined oligomer band by peptide 1a suggests that this constrained Aβ β-hairpin peptide forms a well-defined supramolecular assembly in the membrane-like environment provided by the amphiphilic SDS molecules.

To better understand the assembly of the putative octamer, we prepared and studied peptides 1b–h (Fig. 1c). Peptide 1b (2.5 kDa) lacks a cross-strand disulfide bridge and instead contains the native Gln_15_ and Gly_37_ residues. Peptide 1b does not assemble in SDS-PAGE, migrating just below the 4.6 kDa ladder band (Fig. 2a). Peptide 1c (1.8 kDa) maintains the cross-strand disulfide bridge but lacks the N- and C-terminal residues 12–14 and 38–40. Peptide 1c does not assemble, migrating as a downward-streaking band from the 4.6 kDa ladder band. In contrast to peptide 1c, peptide 1d (2.3 kDa) lacks only the C-terminal residues 38–40 and does assemble. Like peptide 1a, peptide 1d migrates as a downward-streaking band from below the just 26 kDa ladder band. The band formed by peptide 1d suggests an octamer in equilibrium with lower molecular weight species. Peptide 1e (2.1 kDa) lacks only the N-terminal residues 12–14 but does not assemble. Instead, peptide 1e migrates as an upward-streaking band from just below the 4.6 kDa ladder band.

The lack of oligomer formation by peptide 1b reveals that the cross-strand disulfide bridge near the N- and C-terminal residues is necessary for assembly of the putative octamer. It has been noted that SDS is known to contribute to the assembly of small Aβ oligomers.^38^ The differences in the oligomerization of peptides 1a and 1b suggests that stabilization of the β-hairpin conformation by the disulfide bridge, rather than SDS, induces the formation of the putative octamer. The assembly of peptide 1d, in contrast to peptides 1c and 1e, is surprising and suggests that residues 12–14 participate in intermolecular interactions crucial for assembly of the putative octamer.

To further examine the role of interactions between the N- and C-terminal residues of peptide 1a, 12–14 and 38–40, in the assembly of the octamer, we prepared and studied peptide 1f. In peptide 1f, an *N*-methyl group on Gly_38_ is positioned to disrupt hydrogen bonding interactions between residues 12–14 and 38–40. In contrast to peptide 1a, peptide 1f (2.5 kDa) does not assemble as an octamer, but instead migrates as a band between the 10 kDa and 4.6 kDa ladder bands that streaks downward. This result suggests that residues 12–14 and 38–40 hydrogen bond together as part of an extended β-hairpin in the octamer formed by peptide 1a.

To examine whether the putative octamer formed by peptide 1a can accommodate residues 23–29 as a loop, and to better mimic endogenous Aβ β-hairpins and Aβ oligomers, we prepared and studied peptides 1g and 1h. In peptide 1g a loop comprising residues 23–29 replaces the ^δ^Orn turn unit. In contrast to peptide 1a, peptide 1g (3.0 kDa) does not assemble and instead migrates just above the 4.6 kDa ladder band. Peptide 1h is a homologue of peptide 1g, that incorporates an additional cross-strand disulfide bridge replacing Asp_23_ and Gly_29_ to fortify the β-hairpin conformation of the peptide. Peptide 1h (3.0 kDa) migrates as two bands, a lower molecular weight band consistent with a tetramer and a higher molecular weight band consistent with an octamer. The tetramer band migrates between the 10 and 17 kDa ladder bands and is much greater in intensity than the octamer band, which migrates just below the 26 kDa ladder band. The difference in intensity between the tetramer and octamer bands suggest that peptide 1h favors assembly of the tetramer. Thus, it appears that the octamer formed by peptide 1a can accommodate the Aβ_23–29_ loop, but that the loop destabilizes the β-hairpin conformation required for assembly—unless an additional stabilizing disulfide bridge is present, as in peptide 1h.

Peptide 2a is a macrocyclic homologue of peptide 1a with a second ^δ^Orn turn unit connecting the N- and C-terminal residues 12 and 40. Like peptide 1h, peptide 2a (2.6 kDa) migrates as two oligomers, a lower molecular weight oligomer consistent with a tetramer and a higher molecular weight oligomer consistent with an octamer. The tetramer band migrates just above the 10 kDa ladder band, and the octamer band migrates between the 17 and 26 kDa ladder bands. Both bands are equal in their intensity, with the octamer band streaking downward toward the tetramer band, suggesting that the two species are in equilibrium with each other. Peptide 2b is a homologue of peptide 2a that lacks the cross-strand disulfide bridge and does not assemble, instead migrating just below the 4.6 kDa ladder band.

Collectively, the SDS-PAGE studies of peptides 1b–1h and 2a–2b highlight key factors in the formation of an octamer by peptide 1a. The N-terminal residues 12–14 form critical intermolecular contacts in the octamer. The formation of both tetramers and octamers by peptides 1h and 2a indicates that the tetramers and octamers are in equilibrium and suggests that the tetramers may be components of the octamers, for these peptides as well as for peptide 1a. Peptides 1h and 2a bear additional stabilizing constraints not present in peptide 1a—a second disulfide bridge and a second ^δ^Orn turn unit respectively. Rather than stabilizing the octamer, these additional constraints destabilize the octamer and promote the formation of a tetramer, which appears to be a subunit of the octamer. The lack of assembly by peptide 2b, despite the second ^δ^Orn turn unit, suggests that the cross-strand disulfide bridge replacing Gln_15_ and Gly_37_ is essential for octamer assembly.

### Folding of peptides 1a–h and 2a–b

The CD spectrum of peptide 1a is similar to that of a β-sheet, suggesting that the peptide folds as designed into a β-hairpin (Fig. 2b). The CD spectrum of peptide 1a displays a strong negative band centered at *ca.* 212 nm, with increasing ellipticity at lower wavelengths that reaches a maximum at *ca.* 198 nm before decreasing once again. The disulfide bridge in peptide 1a appears to be essential for folding—the CD spectrum of peptide 1b indicates that it does not fold and that a random coil conformation predominates. Peptide 1b displays a strong negative band centered at *ca.* 198 nm and a weak maximum at *ca.* 220 nm (Fig. 2b). The CD spectra of peptides 1c–f and 1h show that these peptides also fold to adopt β-hairpin-like conformations.

Differences between the CD spectra of peptides 1a and 1g reveal that replacing the ^δ^Orn turn unit with Aβ residues 23–29 abrogates β-hairpin folding (Fig. 2c). The CD spectrum of peptide 1g displays a broad and shallow negative band centered at *ca.* 204 nm, which suggests a random-coil-like conformation. Addition of a disulfide linkage between residues 23 and 29 partially restores folding. Thus peptide 1h displays a negative band centered at *ca.* 212 nm with increasing ellipticity at lower wavelengths.

The CD spectra of peptides 2a and 2b suggest that incorporation of a second ^δ^Orn turn unit connecting residues 12 and 40 does not substantially affect folding (Fig. 2c). Peptide 2a displays a strong negative band centered at *ca.* 214 nm with increasing ellipticity at lower wavelengths, similar to peptide 1a, reflecting a β-hairpin-like conformation. Peptide 2b displays a strong negative band centered at *ca.* 200 nm, similar to peptide 1b, indicating a random coil conformation. Thus, the constraint of peptide 1b into a macrocycle but without a disulfide bridge is not sufficient to induce β-hairpin folding.

Correlation of these CD studies with the SDS-PAGE studies suggests that folding is necessary for assembly, but that not all folded β-hairpin peptides can assemble. Key residues and contacts also appear to be necessary to achieve the putative octamer that is observed in SDS-PAGE for peptides 1a, 1d, 1h, and 2a.

### X-ray crystallographic and REMD studies of peptide 2a

**Fig. 3:**
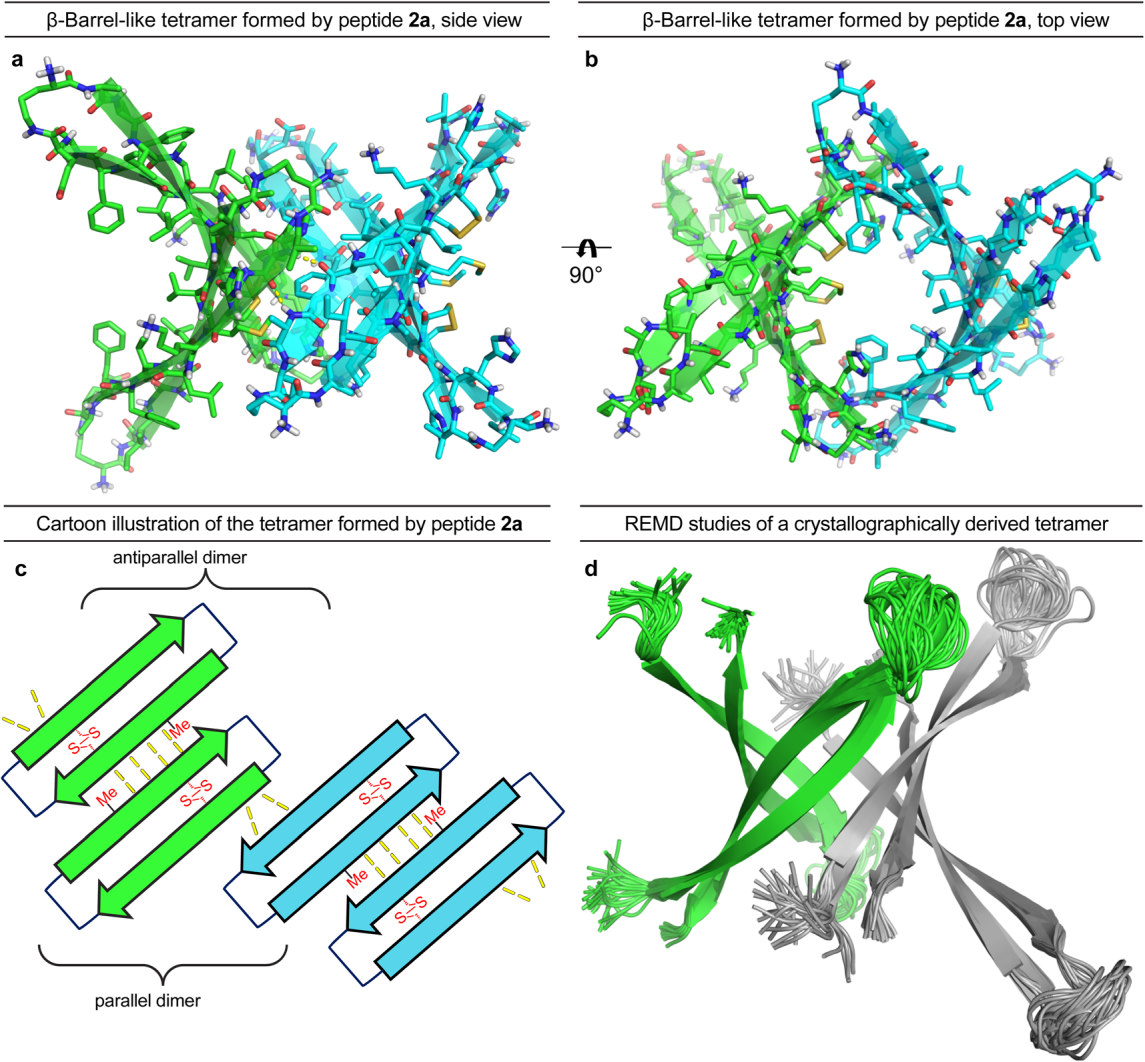
X-ray crystallographic structure of the β-barrel-like tetramer formed by peptide 2a (PDB 7RTZ). (a) Side view of the tetramer. (b) Top view of the tetramer. (c) Cartoon illustration of the parallel and antiparallel β-sheet interactions that stabilize the tetramer. (d) REMD simulation of a tetramer of Aβ_9–42_ based on the β-barrel-like tetramer formed by peptide 2a. Residues 12–22 and 30–40 are constrained to the crystallographic coordinates of peptide 2a.

To gain further insights into the structures of the oligomers observed in SDS-PAGE, we grew crystals of peptide 2a and performed X-ray crystallography. None of the other peptides afforded crystals. The X-ray crystallographic structure of peptide 2a reveals that the peptide assembles to form antiparallel β-sheet dimers that further assemble in a face-to-back fashion to form concatenated β-barrel-like tetramers (Fig. 3, PDB 7RTZ). The core of each β-barrel-like tetramer is lined with hydrophobic residues (Cys_15_, Val_18_, Phe_20_, Ile_32_, Ile_34_, Met_35_, Val_36_ and Cys_37_), which pack against each other while creating a pore through the center, *ca.* 7.5 Å in diameter (ESI Fig. S3b†). ESI Table S1† summarizes the crystallographic properties, conditions, data collection, and model refinement statistics for peptide 2a.

The β-hairpin subunits comprise residues 12–22 and 30–40 and include a disulfide bridge that replaces Gln_15_ and Gly_37_ (ESI Fig. S3a†). The β-hairpin subunits assemble in pairs to form the antiparallel β-sheet dimers (ESI Fig. S3b†), which are stabilized by two pairs of intermolecular hydrogen bonds between Met_35_ and Cys_37_. The two dimers further assemble through parallel β-sheet interactions between His_14_ and Phe_19_ to form the eight-stranded β-barrel-like tetramer (Fig. 3a–c). Molecular modeling suggests that full-length Aβ can assemble in the same fashion as peptide 2a to form a β-barrel-like tetramer (Fig. 3d). Thus, we observed that Aβ_9–42_ could form a β-barrel-like tetramer without steric clashes when modeled into the crystallographic coordinates of the tetramer using replica exchange molecular dynamics (REMD) simulations in implicit solvent to generate realistic conformations of the loops and N- and C-terminal regions.

**Fig. 4:**
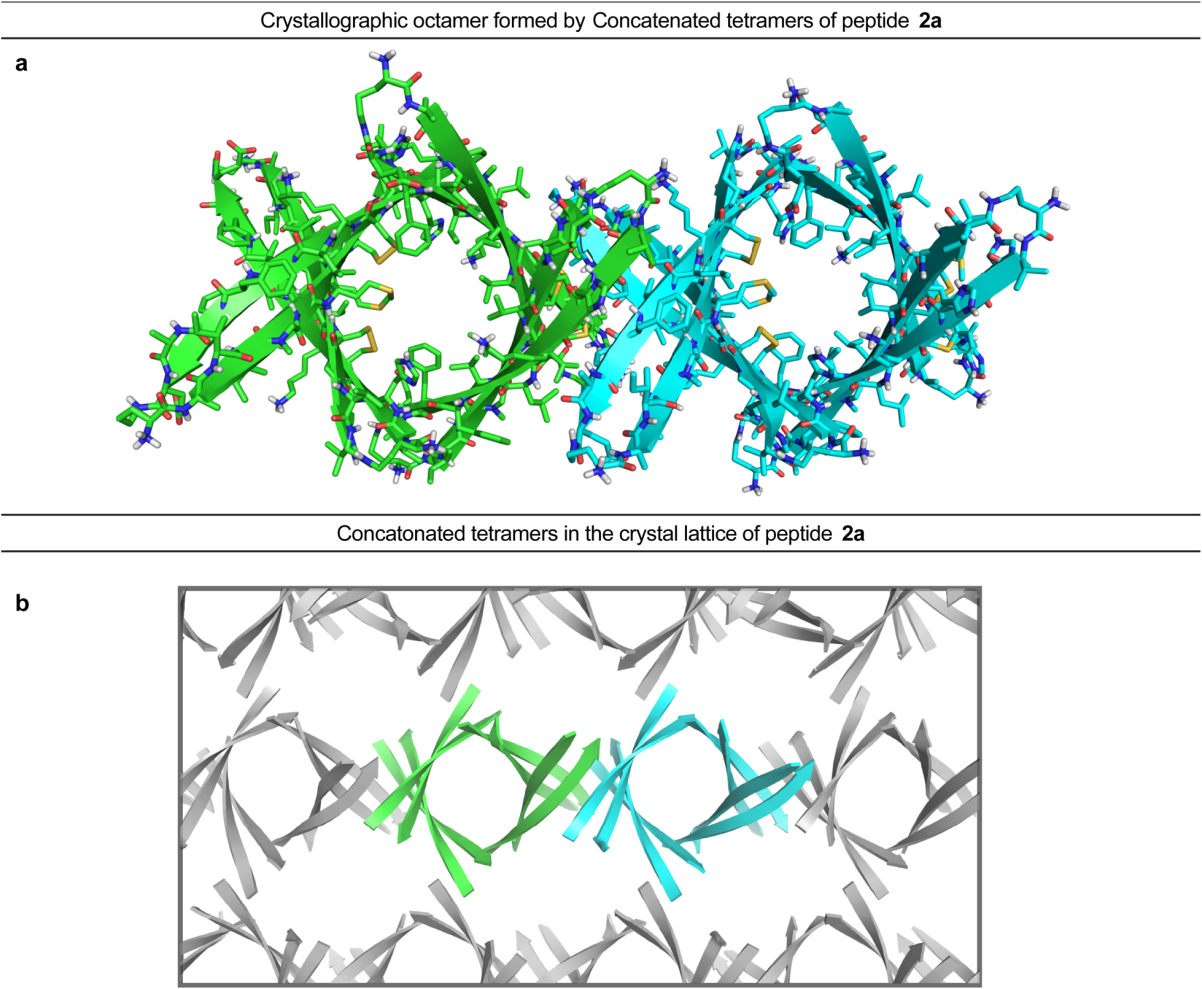
X-ray crystallographic structure of the octamer formed by peptide 2a (PDB 7RTZ). (a) Two concatenated β-barrel-like tetramers form an octamer. (b) Assembly of the tetramers within the crystal lattice of peptide 2a to form concatenated chains of β-barrels.

In the crystal lattice, the β-barrel-like tetramers link together to form concatenated networks of β-barrels running the length of the lattice, with the interface between each tetramer also constituting a β-barrel-like tetramer (Fig. 4). Thus, two tetramers may be thought of as composing an octamer consisting of three linked β-barrels. The two tetramers that make up the octamer are parallel to each other, and the tetramer formed at their interface is perpendicular but otherwise identical. Larger oligomers composed of more tetramers can also be envisioned. The crystallographically observed tetramers and octamers may explain the structures of the tetramers and octamers observed in SDS-PAGE for peptides 1a, 1d, 1h, and 2a, with each tetramer consisting of a single β-barrel and two tetramers further assembling to form an octamer consisting of three concatenated β-barrels.

### Correlating the crystallographic tetramer and octamer with the oligomers observed in SDS-PAGE

**Fig. 5:**
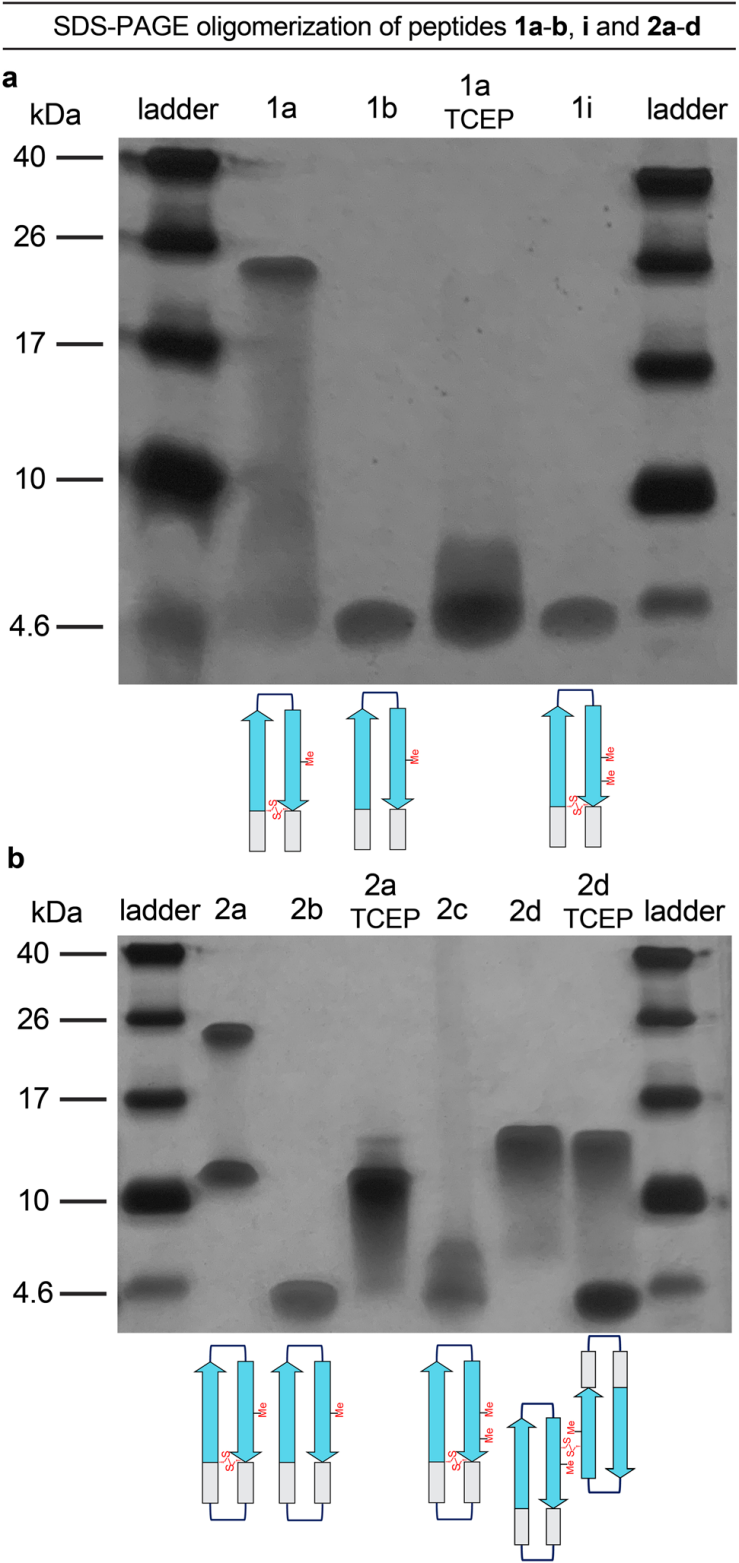
(a) Silver stained SDS-PAGE of peptides 1a, 1b, 1i, and 1a after treatment with TCEP. (b) Silver stained SDS-PAGE of peptides 2a–d, and 2a and 2d after treatment with TCEP. SDS-PAGE was performed in Tris buffer at pH 6.8 with 2% (w/v) SDS on a 16% polyacrylamide gel with 50 µM solutions of peptide in each lane. Treatment with TCEP was performed at 10 mM.

To better understand the tetramers and octamers observed in SDS-PAGE and to correlate these species with the crystallographically observed tetramers and octamers formed by peptide 2a, we performed TCEP reduction, additional *N*-methylation, and disulfide crosslinking experiments (Fig. 5a and b).^39^ Reduction of the disulfide bonds of peptides 1a and 2a with tris(2-carboxyethyl)phosphine (TCEP) disrupts octamer formation in SDS-PAGE. Treatment of peptide 1a with TCEP results in a streaky band that is similar in position to that of peptide 1b, which does not assemble (Fig. 5a). Treatment of peptide 2a with TCEP eliminates octamer formation and gives a downward streaking band at a position that corresponds to a tetramer (Fig. 5b). Disruption of the dimerization interface by *N*-methylation of Met_35_, in peptides 1i and 2c, also disrupts octamer formation (Fig. 5a and b) while partially or fully retaining propensities to adopt β-hairpin-like conformations (ESI Fig. S2†). These experiments demonstrate that disruption of the hydrogen-bonding interfaces or destabilization of component β-hairpins disrupts octamer or tetramer formation.

Covalent crosslinking of the antiparallel β-sheet dimer subunit observed in the X-ray crystallographic structure of peptide 2a stabilizes tetramer formation. Peptide 2d is a homologue of peptide 2b in which Val_36_ is replaced with cysteine and crosslinked intermolecularly. Peptide 2d runs as a tetramer in SDS-PAGE (Fig. 5b). Upon treatment with TCEP, peptide 2d runs as bands that correspond to monomer and tetramer (Fig. 5b). The formation of the monomer upon reduction further correlates the tetramer assembly observed in SDS-PAGE with that observed in the crystal structure. The CD spectrum of peptide 2d resembles a β-sheet, suggesting that unlike peptide 2b, it folds as designed to adopt a β-hairpin like conformations (ESI Fig. S2†).

We performed dynamic light scattering (DLS) studies to further characterize the oligomers formed by peptides 1a, 2a, and 2b in an aqueous environment without SDS. Under the same pH and buffer conditions of the CD experiments, DLS shows that these peptides form aggregates with hydrodynamic diameters of about 2 µm (ESI Fig. S4†). The aggregates observed by DLS are far larger than the tetramer or octamer observed by SDS-PAGE, or the crystallographic tetramer formed by peptide 2a. These observations suggest that peptides derived from the β-hairpin reported by Tycko and coworkers can assemble to form large assemblies in aqueous environments.

### Cytotoxicity and membrane disruption of peptides 1a and 2a

Aβ oligomers have been shown to activate apoptotic pathways *in vivo* and damage artificial membranes *in vitro*.^40–44^ Because the crystallographic β-barrel-like tetramer formed by peptide 2a resembles a pore, we speculated that peptides 1a and 2a may damage cell membranes through a pore-like mechanism of action. To investigate the cytotoxic and membrane disrupting potential of Aβ derived peptides stabilized in the alignment reported by Tycko and coworkers, we carried out a caspase-3/7 activation assay and a dye-leakage assay using peptides 1a and 2a. Peptides 1a and 2a were thus found to activate caspase 3/7, an apoptotic marker, at 12.5–25 µM in SH-SY5Y cells (ESI Fig. S5†). The dye-leakage assay was performed using negatively charged large unilamellar vesicles (LUVs) and peptides 1a and 2a (ESI Fig. S6†). In these experiments, peptides 1a and 2a disrupt anionic lipid membranes at 0.6–2.7 µM. Together, these data suggest that peptides 1a and 2a have limited potential to mimic some of the apoptotic and membrane-damaging behaviors of oligomers formed by full-length Aβ.

### Molecular dynamics simulation of an Aβ tetramer in a lipid bilayer membrane

**Fig. 6:**
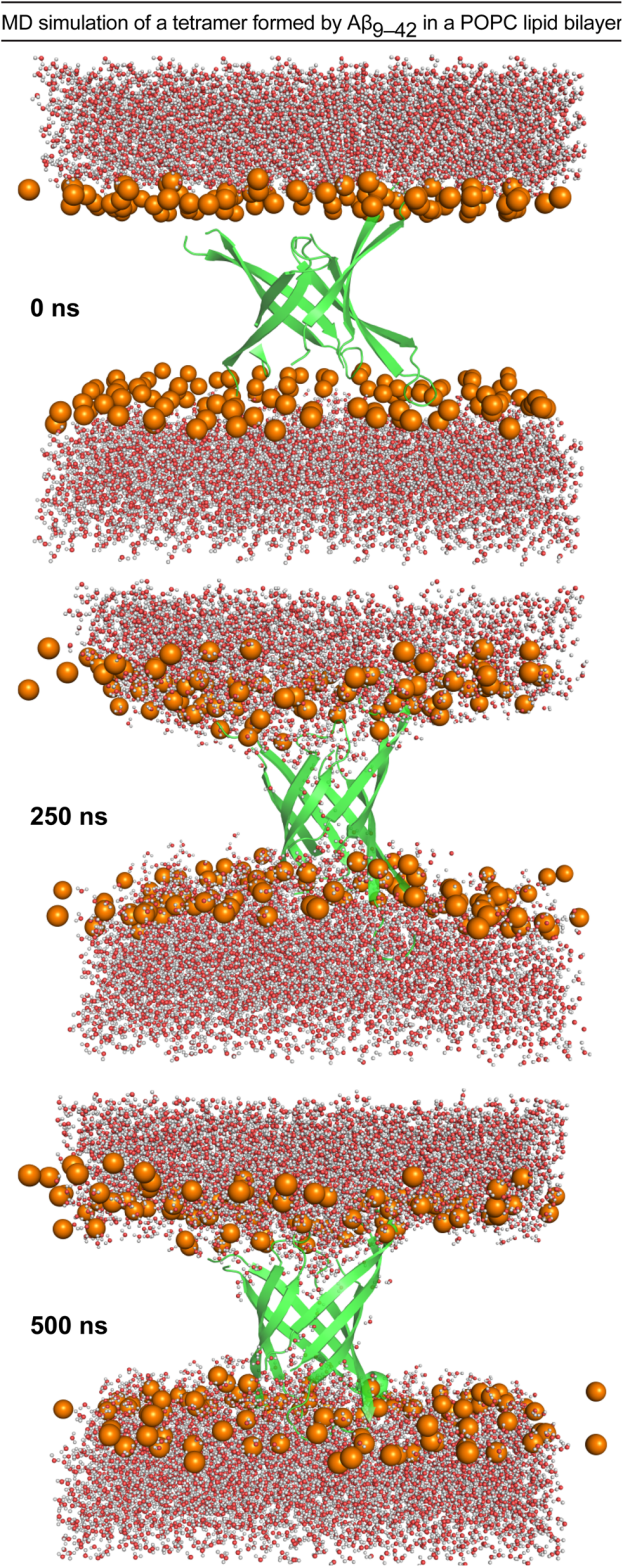
Configuration snapshots from an all-atom molecular dynamics simulation of a β-barrel-like tetramer formed by Aβ_9–42_ in a POPC lipid bilayer at 0 ns, 250 ns, and 500 ns. The Aβ_9–42_ tetramer is shown as a secondary structure representation. The lipid phosphate P atoms are shown as orange spheres, and the water molecules are shown in ball-and-stick representations colored by atom (O, red; H, white). The rest of the system is omitted for clarity. The simulation was set up using CHARMM-GUI,^54,55^ and run for 500 ns at constant temperature and pressure using NMAD 2.14 (ref. 56) with the CHARMM36 forcefield.^57,58^ Molecular graphics and simulation analyses were generated with VMD 1.9.3.^59^

We performed molecular dynamics (MD) simulations using the X-ray crystallographic structure of peptide 2a to explore the consequences of inserting the β-barrel-like tetramer into a membrane.^4,45–49^ We thus generated a model of an Aβ_9–42_ β-barrel-like tetrameric assembly embedded in a fully hydrated POPC lipid bilayer from the crystallographic structure of peptide 2a and the REMD simulation and performed a 500 ns all-atom MD simulation (Fig. 6 and ESI S7†). The incorporation of the tetramer in the lipid bilayer results in a reduction of the lipid bilayer thickness around the β-barrel-like assembly (ESI Fig. S8†) as lipid phosphate groups and water molecules interact with polar moieties in the β-hairpins connecting loops and termini (Fig. 6 and ESI S9†). Water molecules cross the membrane through the tetramer pore, and also interact with polar moieties on the outer surface of the tetramer (Fig. 6 and ESI S10 and S11†). Although these results indicate that it is possible for a tetrameric assembly of peptide 2a to disrupt the membrane by forming pores, they cannot establish whether the caspase 3/7 activation and dye leakage observed for peptides 1a and 2a are caused by pore formation.^50,51^ The caspase 3/7, dye-leakage, and simulation data suggest that peptides 1a and 2a, and other peptides stabilized in the β-hairpin alignment described by Tycko and coworkers can interact with lipid membranes in a manner that our laboratory is beginning to explore.^52,53^

## Conclusion

β-Hairpins are a key structural component of Aβ oligomers and may contribute directly to oligomer heterogeneity through variation in hairpin alignment and topology. In this investigation, we studied the assembly and structure of oligomers formed by peptides derived from Aβ_12–40_ that were stabilized in the β-hairpin alignment reported by Tycko and coworkers. SDS-PAGE and X-ray crystallography reveal that Aβ derived β-hairpins in this alignment assemble to form β-barrel-like tetramers that concatenate to form octamers. We envision that Aβ β-hairpins in other alignments might form other oligomers, with different structures, stoichiometries, and stabilities—and that all of these β-hairpins and oligomers exist in equilibrium. Earlier investigations from our laboratory on related Aβ derived peptide systems have revealed the assembly of dimers, trimers, tetramers, hexamers, octamers, and dodecamers that differ substantially in assembly and structure. Further, mutations in familial Alzheimer's disease that alter the biophysical properties of the Aβ peptide may have a significant impact on Aβ folding and assembly.^33,60^ These findings would suggest that not all β-hairpins formed by the Aβ peptide have an equal propensity to fold and assemble.

It is noteworthy that peptide 2a assembles to form oligomers of the same stoichiometry as the tetramer and octamer reported by Carulla and coworkers, but with significant differences in structure.^4^ The tetramer reported by Carulla and coworkers is planar and does not form a β-barrel-like assembly; further the octamer is proposed to adopt a β-sandwich conformation. Neither the planar tetramer nor the β-sandwich contain a pore. Instead, the β-barrel-like tetramer formed by peptide 2a more closely resembles the hexameric “cylindrin” formed by peptide fragments of αB crystallin, reported by Eisenberg and coworkers.^61^ Cylindrin-like assemblies from fragments of Aβ have also been reported.^62–64^ The tetramers and octamers described herein provide additional models of how Aβ may assemble into oligomers in membrane-like environments and the crystal state.

## Data availability

Data analyzed in this study are available from the authors on request. Crystallographic data for peptide 2a has been deposited at the Protein Data Bank under PDB number 7RTZ and can be obtained from https://doi.org/10.2210/pdb7RTZ/pdb.

## Author contributions

Tuan D. Samdin: conceptualization, methodology, investigation, validation, formal analysis, writing – original draft, writing – reviewing & editing, visualization. Chelsea R. Jones: investigation. Gretchen Guaglianone: investigation. Adam G. Kreutzer: conceptualization, methodology, investigation. J. Alfredo Freites: methodology, software, validation, formal analysis, resources, visualization, supervision. Michał Wierzbicki: investigation. James S. Nowick: conceptualization, methodology, resources, writing – reviewing & editing, supervision, funding acquisition.

## Conflicts of interest

The authors declare no competing financial interest.

## Supplementary Material

SC-015-D3SC05185D-s001

SC-015-D3SC05185D-s002
