# Supplementary material for: A β-barrel-like tetramer formed by a β-hairpin derived from Aβ
Source: Chem Sci. 2023 Nov 28;15(1):285–97. doi: 10.1039/d3sc05185d (PMC10732006; doi:10.1039/d3sc05185d)
Supplement: SC-015-D3SC05185D-s001 [file SC-015-D3SC05185D-s001.pdf]

Electronic Supplementary Material (ESI) for Chemical Science.
This journal is © The Royal Society of Chemistry 2023

Supporting information for:

A β-Barrel-Like Tetramer Formed by a β-Hairpin Derived From Aβ

Tuan D. Samdin[a], Chelsea R. Jones[a], Gretchen Guaglianone[a], Adam G. Kreutzer[a], J. Alfredo Freites[a]; Michał Wierzbicki[a], and James S. Nowick*[a,b]

[a]Department of Chemistry, University of California, Irvine
[b]Department of Pharmaceutical Sciences, University of California, Irvine
Irvine, California 92697, United States

Table of Contents

Supporting Figures and Tables 1
- Figure S1 3
- Figure S2 4
- Table S1 5
- Figure S3 6
- Figure S4 7
- Figure S5 8
- Figure S6 9
- Figure S7 10
- Figure S8 11
- Figure S9 12
- Figure S10 13
- Figure S11 14

Materials and Methods 15
- General information 15
- Synthesis of peptides **1a–i** 16
- Synthesis of peptides **2a–d** 19
- SDS-PAGE, TCEP reduction, and silver staining 23
- Circular dichroism spectroscopy 24
- Crystallization of peptide **2a** 24
- X-ray crystallographic data collection, data processing, and structure determination of peptide **2a** 25

Replica Exchange Molecular Dynamics Simulation of an Aβ_{9-42} β-barrel-like tetramer 25

Molecular Dynamics Simulation of an Aβ_{9-42} β-barrel-like tetramer in a lipid membrane 26

Caspase-3/7 activation assay 27

Dye-Leakage Assay 28

Dynamic light scattering 30

References 31

Characterization Data 34

Characterization of peptide **1a** 34

Characterization of peptide **1b** 36

Characterization of peptide **1c** 38

Characterization of peptide **1d** 40

Characterization of peptide **1e** 42

Characterization of peptide **1f** 44

Characterization of peptide **1g** 46

Characterization of peptide **1h** 48

Characterization of peptide **1i** 50

Characterization of peptide **2a** 52

Characterization of peptide **2b** 54

Characterization of peptide **2c** 56

Characterization of peptide **2d** 59

Supporting Figures and Tables

Tetramer of A$\beta_{1–42}$, formed by the Aβ β-hairpin reported by Carulla et al. (PDB 6RHY), *Nat. Commun.*, **11**, 1–14 (2020)

a

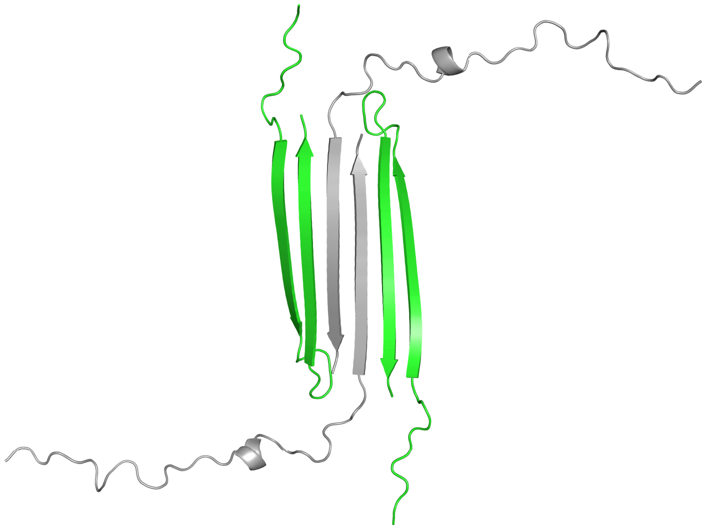

Barrel-like-hexamer of A$\beta_{16–42}$, formed by the Aβ β-hairpin reported by Härd et al., *Angew. Chemie - Int. Ed.*, **53**, 12756–12760 (2014)

b

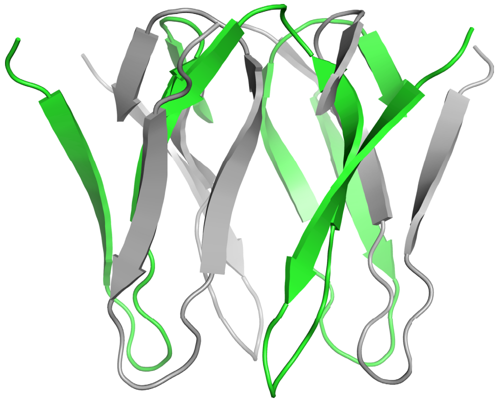

Aβ_{40} Fibrils with Aβ β-hairpins proposed by Tycko et al., *Proc. Natl. Acad. Sci.*, **118**, e2023089118 (2021)

c

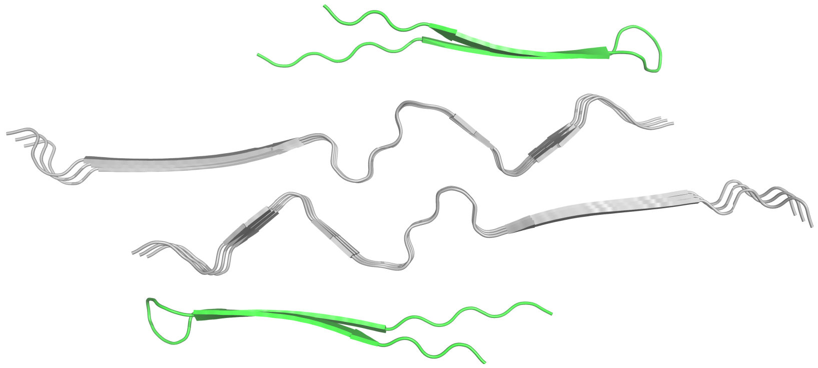

Figure S1. (a) NMR structure of the tetramer formed by the β-hairpin reported by Carulla and coworkers (PDB 6RHY) (b) NMR structure of the barrel-like hexamer formed by an Aβ derived β-hairpin reported by Härd and coworkers (c) Aβ_{40} fibril structure with proposed Aβ_{40} β-hairpins reported by and coworkers.

CD spectra of peptides **1a**, **i** and **2a**, **c-d**

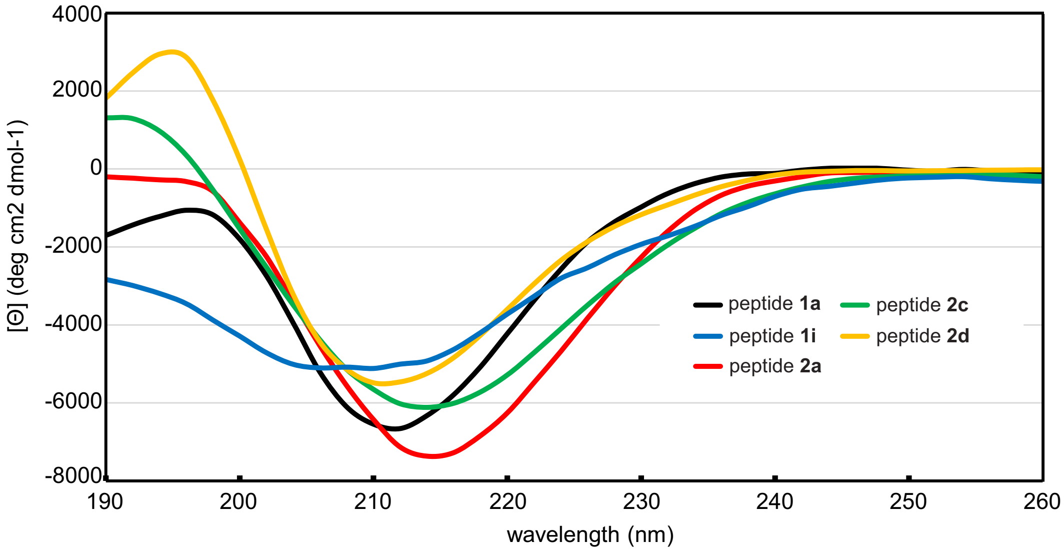

Figure S2. Circular dichroism (CD) spectra of peptides **1a**, **1i**, **2a**, and **2c–d**. CD spectra were acquired for each peptide at 50 µM in 10 mM phosphate buffer at pH 7.4; ellipticity was normalized for the number of residues in each peptide.

Supplementary Table 1. Crystallographic properties, crystallization conditions, data collection, and model refinement statistics for peptide **2a**.

peptide	**2a**
Wavelength (Å)	1.0
Resolution range	36.52 - 2.1 (2.175 - 2.1)
Space group	P 41 21 2
Unit cell	44.3265, 44.3265, 64.4265 90, 90, 90
Total reflections	198405 (19442)
Unique reflections	4086 (393)
Multiplicity	48.6 (49.5)
Completeness (%)	99.83 (100.00)
Mean I/sigma(I)	21.83 (2.00)
Wilson B-factor	43.80
R-merge	0.3661 (1.108)
R-meas	0.37 (1.118)
R-pim	0.05249 (0.1504)
CC1/2	0.998 (0.795)
CC*	1 (0.941)
Reflections used in refinement	4079 (393)
Reflections used for R-free	406 (39)
R-work[a]	0.2639 (0.3241)
R-free[a]	0.2941 (0.3099)
CC(work)	0.911 (0.810)
CC(free)	0.879 (0.730)
Number of non-hydrogen atoms	376
RMS(bonds)	0.016
RMS(angles)	0.89
Ramachandran favored (%)	100.00
Ramachandran allowed (%)	0.00
Ramachandran outliers (%)	0.00
Rotamer outliers (%)	0.00
Clashscore	5.59
Average B-factor[a]	54.16
crystallization conditions	0.2 M Mg acetate tetrahydrate, 0.1M Na cacodylate trihydrate (pH 7.0), 30% v/v 2-methyl-2,4-pentanediol

[a]The R-work and R-free values are somewhat higher than would be expected for an X-ray crystallographic structure at 2.1 Å resolution and the average B-factor is relatively large. We believe that this is a reflection of the loose packing the lattice, which consist of columns of concatenated tetramers with only loose contacts between the columns. The contacts between the columns involve the flexible loops regions of the peptide and should result in considerable mobility and disorder. The higher R-values do not reflect a lack of data in the higher resolution shells, as the completeness in the highest resolution shell is 99.8% and the figure of merit in that shell is 0.62, which both fall in line with the values in the other resolution shells.

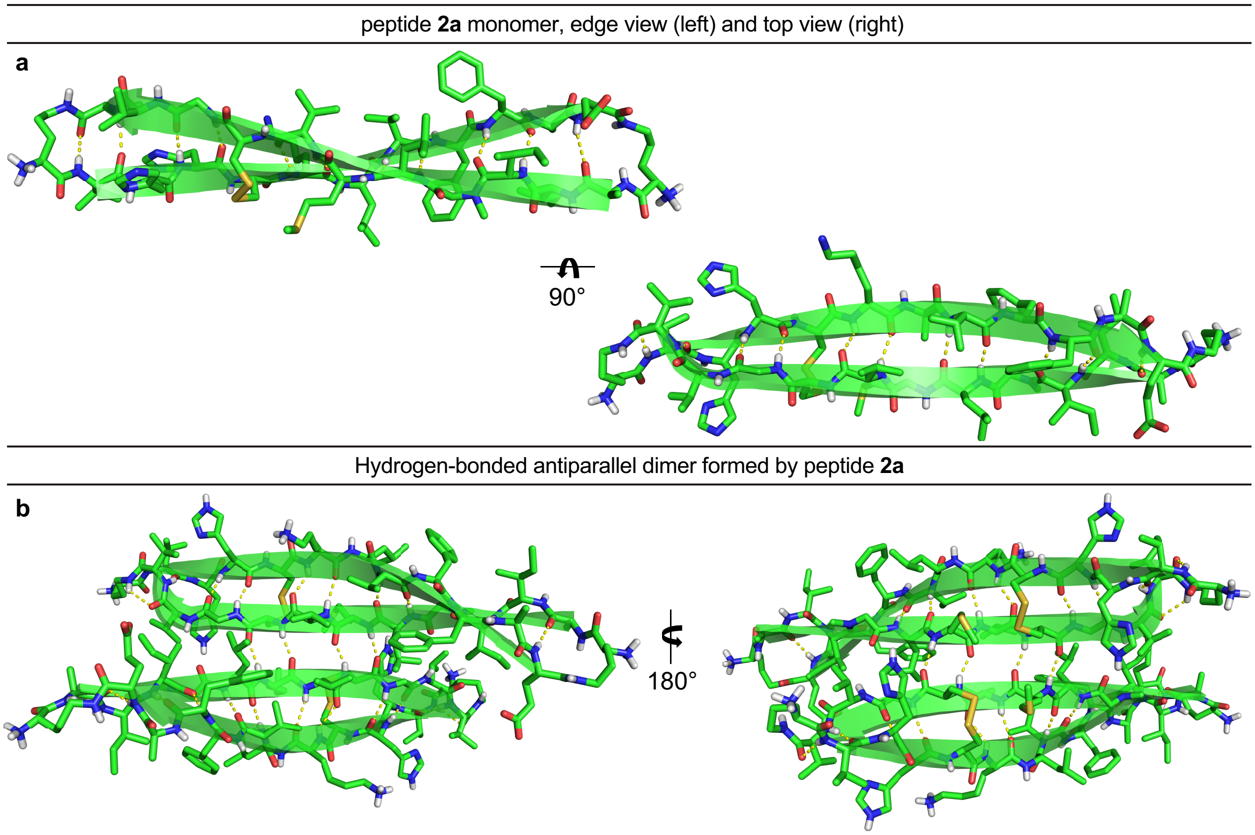

Figure S3. X-ray crystallographic structure of the (a) monomer and (b) dimer formed by peptide **2a** (PDB 7RTZ).

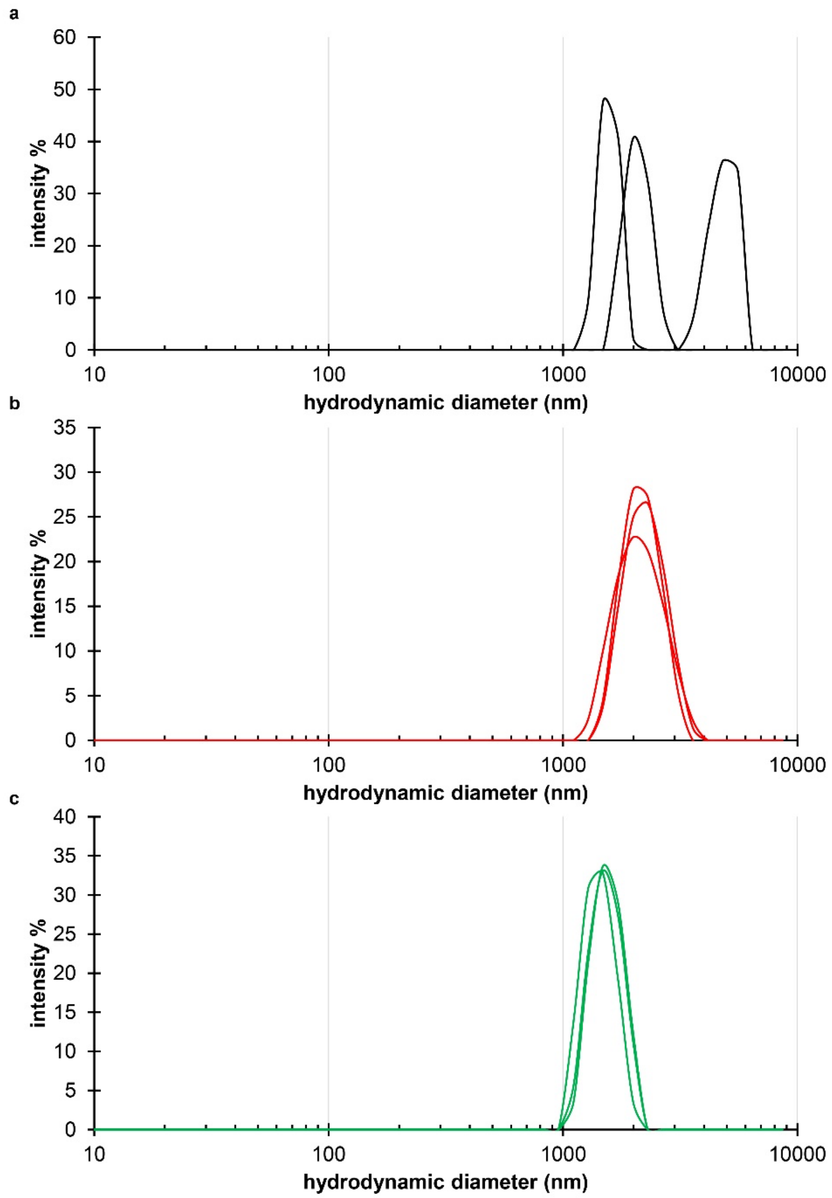

Figure S4. Dynamic light scattering (DLS) of peptides (a) **1a**, (b) **2a**, and (c) **2b**. DLS spectra for each peptide were acquired at 70 µM in 10 mM phosphate buffer at pH 7.4.

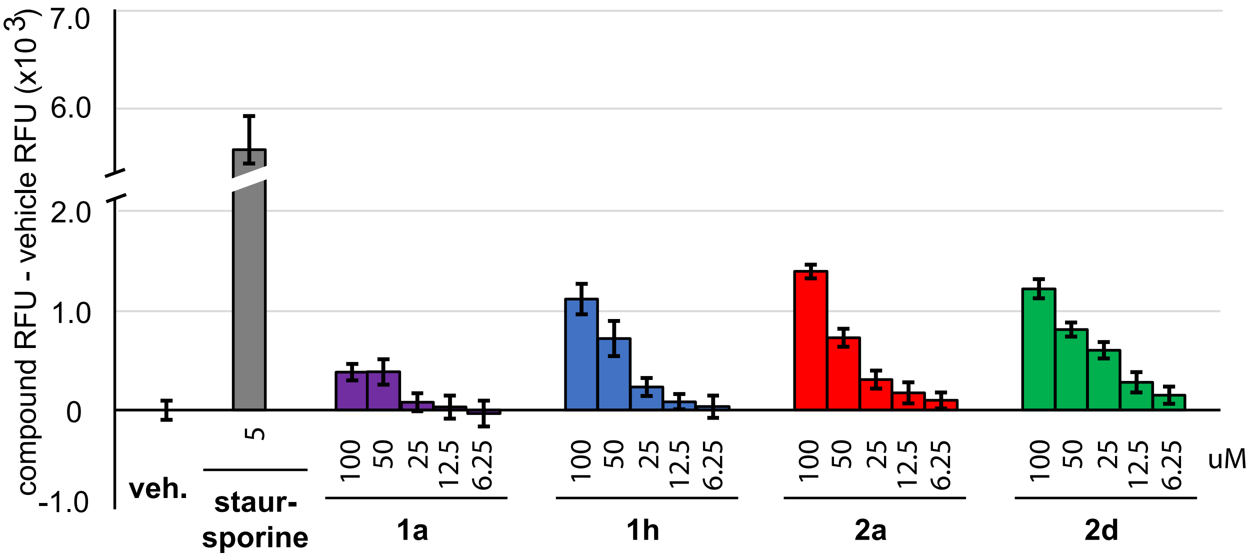

Figure S5. Caspase-3 activation assay in SH-SY5Y cells treated with peptides **1a 1h**, **2a**, and **2d** in SH-SY5Y. Data represent the mean of three replicate wells ± s.d. Deionized water (vehicle, veh.) was used as a negative control. Staurosporine (5 μM) was used as a positive control.

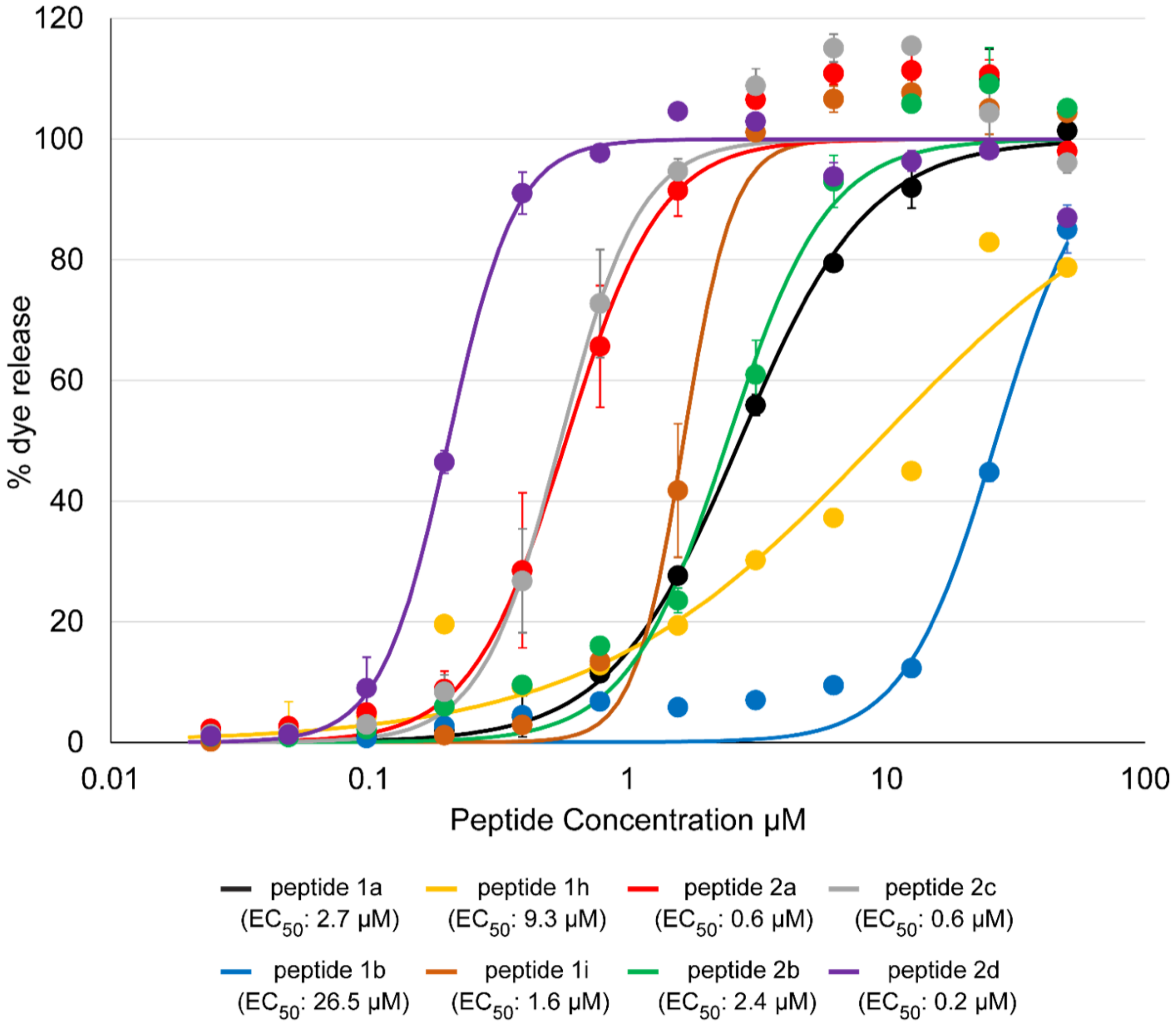

Figure S6. Membrane destabilization by peptides **1a**, **1b**, **1h**, **1i**, **2a–2d** as determined by dye-leakage assays. Large unilamellar vesicles (LUVs) consisting of 1:1 PC/PS containing calcein were treated with peptide at concentrations ranging from 0.24 to 50 µM. Data points represent averages of three replicate runs, error bars represent the corresponding standard deviations (but are obscured by data points), and curves show nonlinear regression fits to the data.

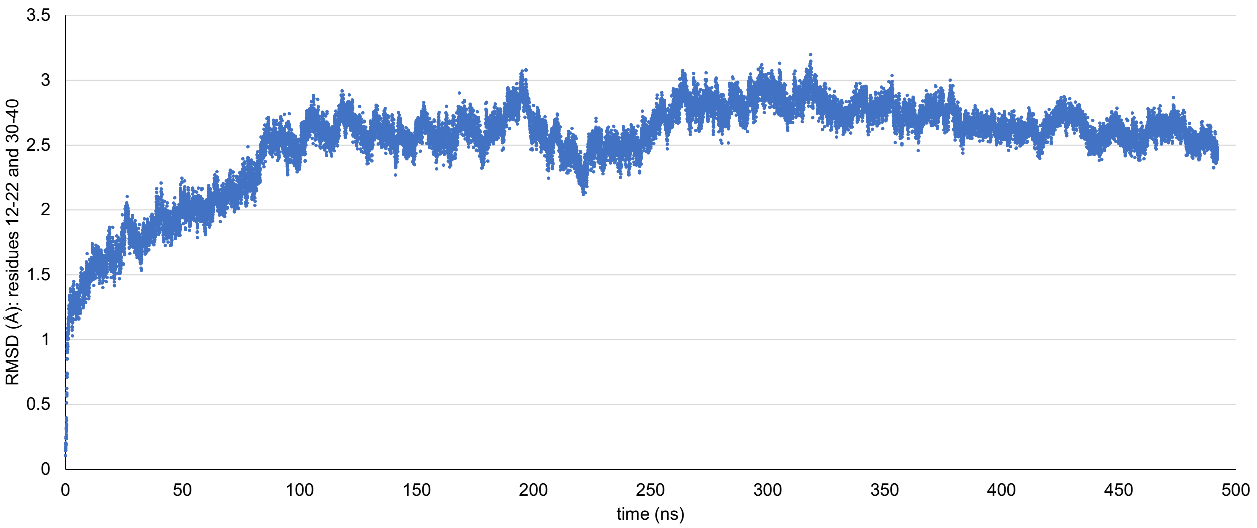

Figure S7. Molecular dynamics simulation of a β-barrel-like tetramer formed by A$\beta_{9\text{–}42}$ in a POPC lipid bilayer. The simulation was set up using CHARMM-GUI[36,38], and run for 500 ns at constant temperature and pressure using NAMD 2.14[39] with the CHARMM36 forcefield.[37,40] Cα RMSD from the crystallographic structure, residues 12–22 and 30–40, was measured over time.

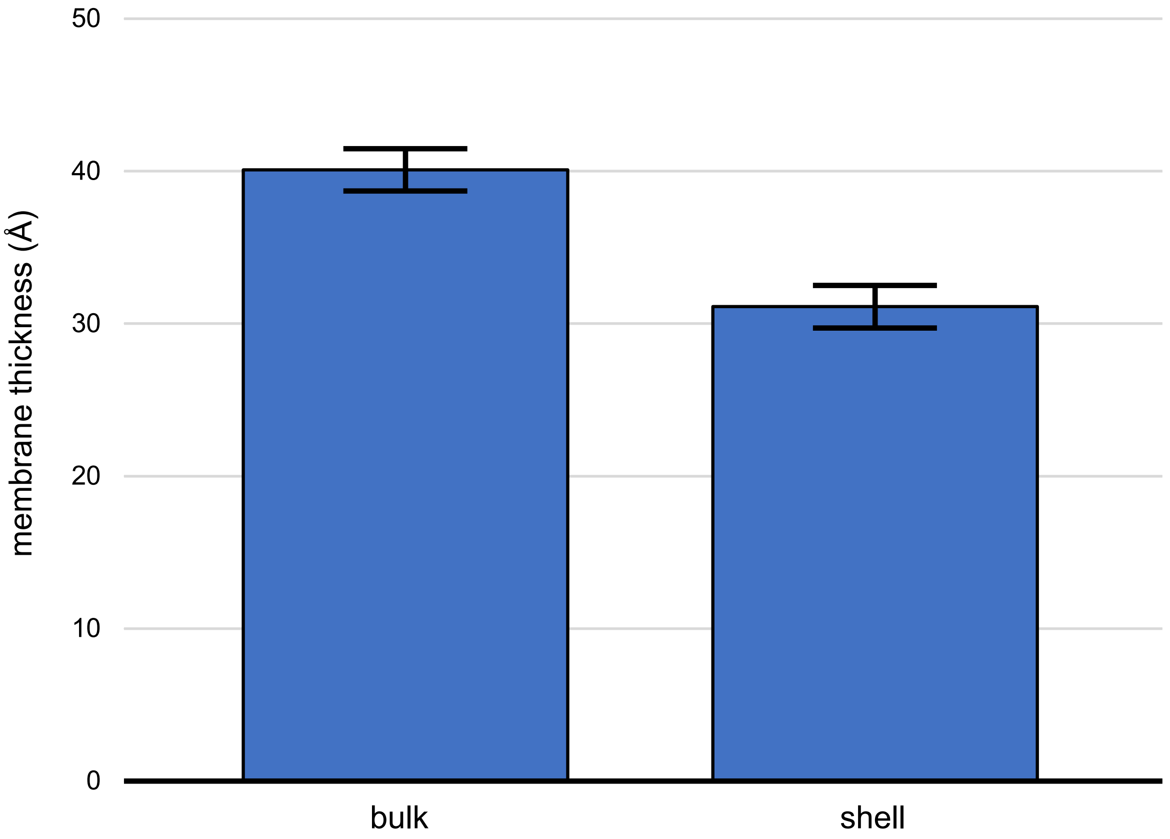

Figure S8. Effect of the β-barrel-like tetramer on the lipid bilayer thickness measured as the mean distance between lipid phosphate P atoms in opposite lipid bilayer leaflets. The values shown correspond to the lipids in immediate neighborhood of the tetramer (solvation shell, P atoms within 6.5 Å of any protein heavy atom), and the lipids not interacting with the tetramer (bulk, P atoms not within 12 Å of any protein heavy atom). The error bars are 95% confidence intervals.

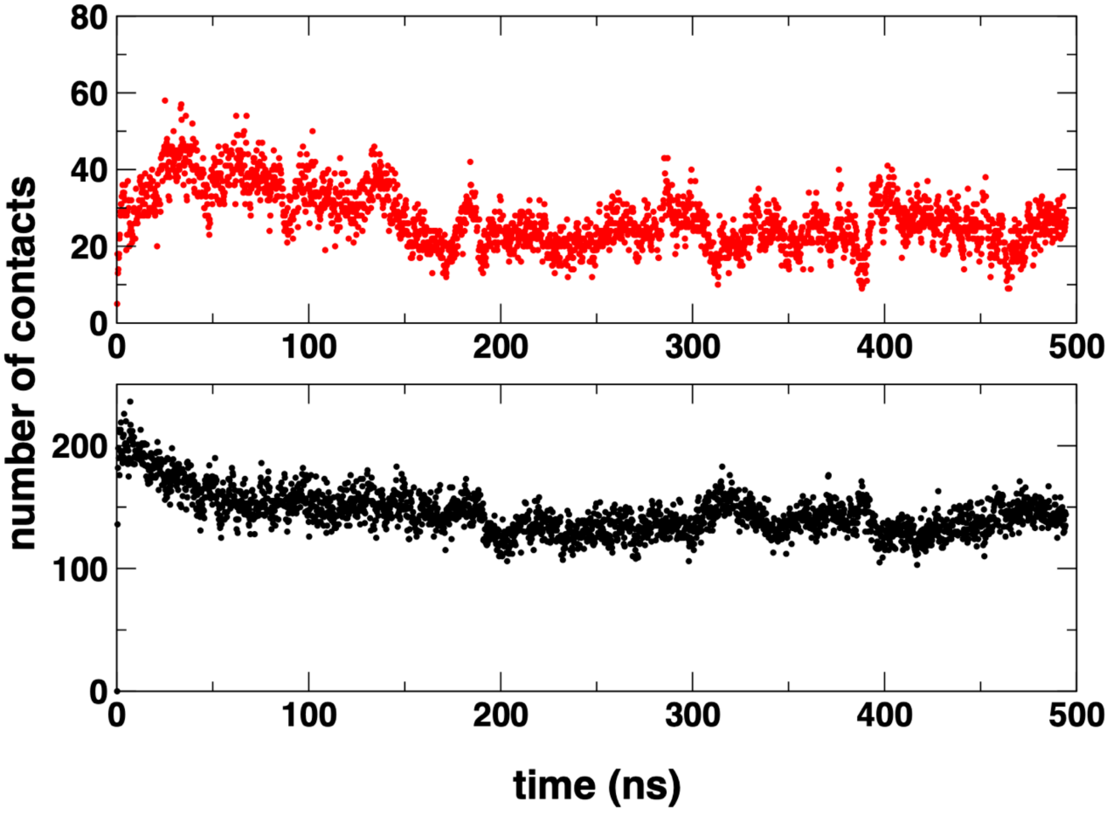

Figure S9. The number of contacts between protein polar moieties and (red) lipid phosphate groups and (black) waters within the lipid bilayer hydrocarbon core. Contacts were defined as any interatomic distance of less than 3.5 Å. The lipid hydrocarbon core was defined as being within ±12 Å of the center of the lipid bilayer.

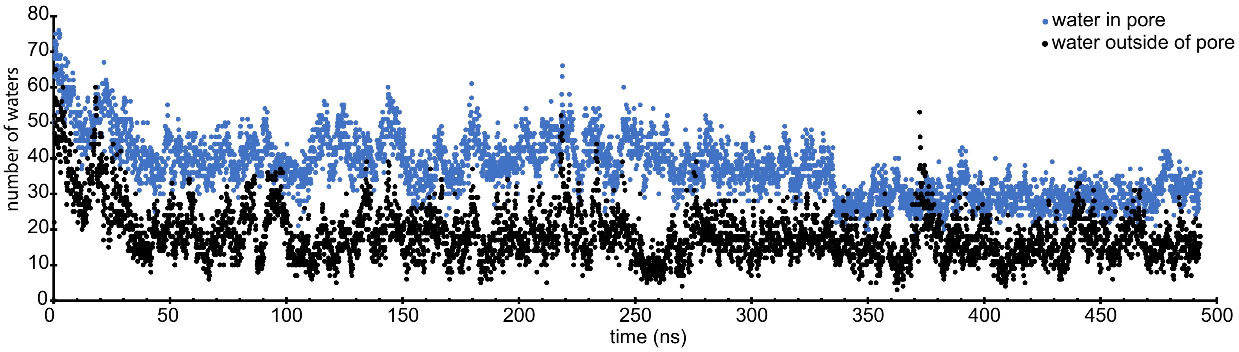

Figure S10. The number of waters within the lipid bilayer hydrocarbon core, inside (blue) and outside (black) the tetramer pore, as a function of time. The lipid hydrocarbon core was defined as being within ±12 Å of the center of the lipid bilayer. The radius of gyration of the tetramer (12.4 Å) was used to define the inside and outside regions of the tetramer pore. Waters within 12.2 Å of the pore axis were considered to be inside the pore, while waters not within 12.6 Å of the pore axis were considered to be outside the pore.

Distribution of water molecules within the tetramer formed by A$\beta_{9–42}$ during the MD simulation

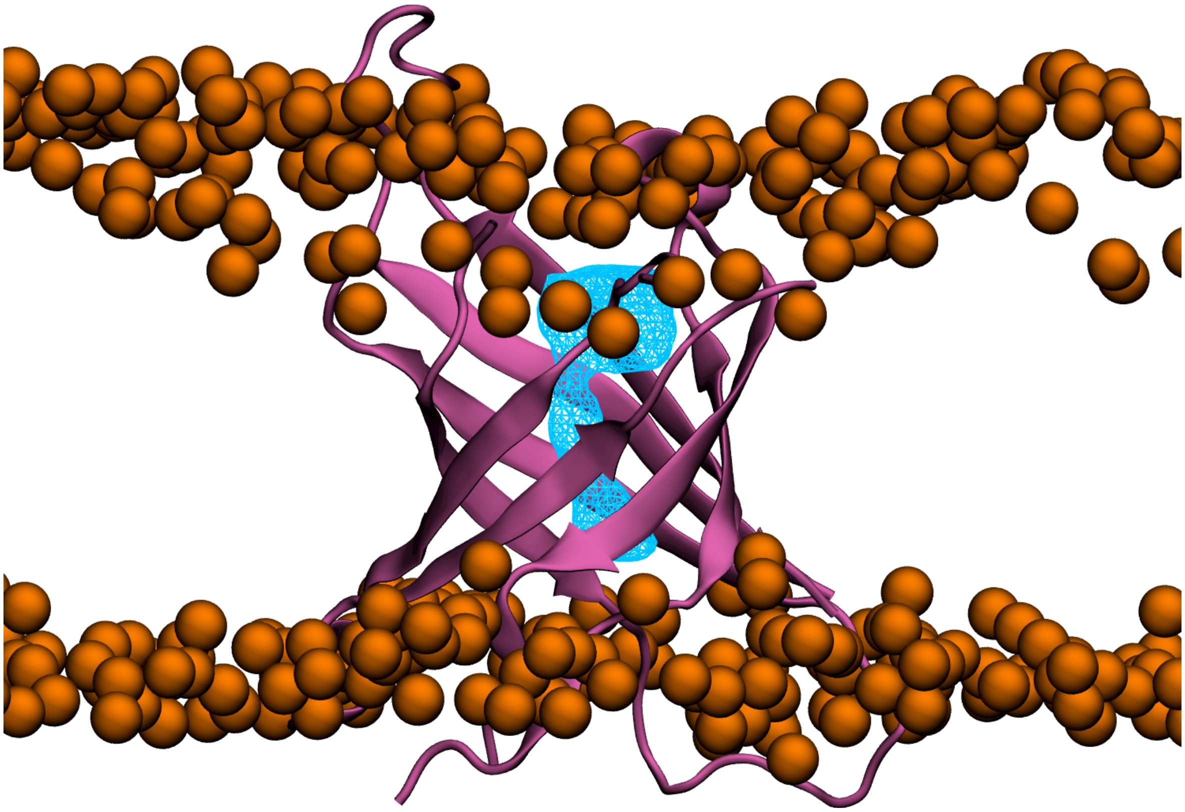

Figure S11. Configuration snapshot from the molecular dynamics simulation of the β-barrel-like tetramer assembly in a lipid bilayer. The surface corresponding to a 24% occupancy by water molecules within the pore over the last 400 ns of the simulation trajectory is shown in blue. The tetramer in purple in secondary structure representation, and the lipid carbonyl carbons as orange spheres. The rest of the system was omitted for clarity.

Materials and Methods[1]

General information

All chemicals were used as received unless otherwise noted. Methylene chloride (CH_2Cl_2) was passed through alumina under nitrogen prior to use. Anhydrous, amine-free *N,N*-dimethylformamide (DMF) was purchased from Alfa Aesar. Deionized water (18 MΩ) was obtained from a Barnstead NANOpure Diamond water purification system. Analytical reverse-phase HPLC was performed on an Agilent 1260 Infinity II instrument equipped with a Phenomonex Aeris PEPTIDE 2.6u XB-C18 column. Preparative reverse-phase HPLC was performed on a Rainin instrument equipped with an Agilent Zorbax SB-C18 column. HPLC grade acetonitrile and deionized water, each containing 0.1% trifluoracetic acid (TFA), were used for analytical and preparative reverse-phase HPLC. All peptides were prepared and used as the trifluoroacetate salts and were assumed to have one trifluoracetic acid molecule per amine group on each peptide.

Synthesis of peptides **1a–i**

a. Loading the resin. For peptides **1a–b** and **1e–h**, 2-chlorotrityl chloride resin (300 mg, 1.4 mmol/g) was added to a Bio-RAD Poly-Prep chromatography column (10 mL). Dry CH_2Cl_2 (8 mL) was used to suspend and swell the resin for 30 min with gentle rocking. The solution was drained from the resin and a solution of Fmoc-Val-OH (0.78 equiv, 50 mg, 0.33 mmol) in 6% (v/v) 2,4,6-collidine in dry CH_2Cl_2 (8 mL) was added immediately and the suspension was gently rocked for 12 h. The solution was then drained and a mixture of CH_2Cl_2/MeOH/*N*,*N*-diisopropylethylamine (DIPEA) (17:2:1, 10 mL) was added immediately. The resin was gently rocked for 1 h, to cap the unreacted 2- chlorotrityl chloride resin sites. The resin was then washed twice with dry CH_2Cl_2 and dried by passing nitrogen through the vessel. This procedure typically yields 0.18 mmol of loaded resin (0.6 mmol/g loading).

For peptides **1c–d**, Rink amide AM resin (300 mg, 0.68 mmol/g) was added to a Bio-RAD Poly-Prep chromatography column (10 mL). Dry DMF (8 mL) was used to suspend and swell the resin for 30 min with gentle rocking. The solution was drained from the resin and a solution of 20% (v/v) piperidine in DMF was added immediately and the suspension was gently rocked for 1 h. The 20% (v/v) piperidine in DMF was drained from the resin, and the resin was then washed twice with dry DMF. A solution of Fmoc-Cys-Trt (5 equiv, 600 mg, 1.02 mmol), HATU (4.5 equiv), HOAt (4.5 equiv), in 20% (v/v) 2,4,6-collidine in dry CH_2Cl_2 (8 mL) was added immediately and the suspension was gently rocked for 12 h. The solution was then drained and a mixture of acetic anhydride/pyridine (3:2, 8 mL) was added. The resin was gently rocked for 0.5 h, to cap the unreacted rink amide resin sites. The resin was then washed twice with dry CH_2Cl_2 and dried by passing nitrogen through the vessel. This procedure typically yields 0.18 mmol of loaded resin (0.6 mmol/g loading).

b. Manual peptide coupling. The resin loaded with Boc-Orn(Fmoc) was suspended in dry DMF and then transferred to a solid-phase peptide synthesis vessel. For peptides **1a–b** and **1e–h**, residues 40 through 32 were manually coupled using Fmoc-protected amino acid building blocks. For peptides **1c–d**, residues 37 through 32 were manually coupled using Fmoc-protected amino acid building blocks. Each manual coupling cycle consisted of *i*. Fmoc-deprotection with of 20% (v/v) piperidine in DMF for 5 min at ambient temperature (5 mL), *ii*. washing with dry DMF (2x, 5 mL), *iii*. coupling of the amino acid (0.44 mmol, 4 equiv) with HCTU (174 mg, 0.44 mmol, 4 equiv) in 20% (v/v) 2,4,6-collidine in dry DMF (5 mL) for 30 min, and *iv.* washing the with dry DMF (2x, 5 mL). Residue 32, which follows N-Me-Gly_{33}, was double coupled (4 equiv per coupling) using HATU (6 equiv) and HOAt (6 equiv) for 1 hr per coupling to ensure complete reaction. (We have found that coupling after N-methyl amino acids is difficult and requires rigorous coupling to minimize incomplete reaction.[2])

c. Microwave-assisted coupling of residues 31 through 12. For peptides **1a–i**, a CEM Liberty Blue Automated Microwave Peptide Synthesizer was used to couple residues 31 to 12. For peptides **1a–f** and **1i**, Fmoc-Orn(Dde)-OH replaces residues 23–29 in the natural sequence. Each coupling cycle consisted of *i.* Fmoc-deprotection with 20% (v/v) piperidine with 0.1 M Oxyma Pure in DMF for 2 min. at 50 ºC, *ii*. washing with DMF (3x), *iii*. coupling of the amino acid (0.75 mmol, 5 equiv) in the presence of HCTU (0.675 mmol, 4.5 equiv) and 20% (v/v/) *N*-methylmorpholine (NMM) in DMF for 10 min. at 50 ºC, iv. washing with DMF (3x).

d. Cleavage and global deprotection of the peptide from resin. The resin was then transferred to a 10-mL Bio-Rad Poly-Prep chromatography column, and washed 3x with dry CH_2Cl_2. The linear

peptide was cleaved from the resin by rocking the resin for 1 h with a solution of trifluoracetic acid (TFA) (TIPS)/H_2O (18:1:1, 10 mL). During the 1 h deprotection, two 50-mL conical tubes containing 40-mL of dry ether were chilled on dry ice. The solution was drained and split between the two conical tubes of ether. The tubes were then centrifuged at 800 x g for 25 min. The ether supernatant was poured off and the pelleted peptide was dried overnight under vacuum.

e. Cysteine zxidation to form the disulfide bridge. The crude, pelleted peptide was resuspended in a solution of 20% v/v dimethyl sulfoxide (DMSO), 50 mL in each tube. The suspension was gently rocked at room temperature for 48 h. The reaction mixture was concentrated under reduced pressure until only DMSO remained.

f. Reverse-phase HPLC purification. The peptide: DMSO mixture was dissolved in H_2O and acetonitrile (8:2, 5 mL), and the solution was filtered through a 0.2 µm syringe filter and purified by RP-HPLC. The solution of crude peptide was injected at 20% acetonitrile and eluted with a gradient of 20-60% CH_3CN over 90 min. Each peptide eluted between 29-36% CH_3CN. The collected fractions were analyzed by analytical HPLC and MALDI-TOF, and the pure fractions were concentrated by rotary evaporation and lyophilized. Typical syntheses yielded between 5 and 62 mg of the peptide as the TFA salt.

*g. Cysteine oxidation and reverse-phase HPLC purification of peptide **1h***. Peptide **1h** contains two disulfide bridges formed by a Cys(Trt) protected pair that replaces residues 15 and 37, and a Cys(Acm) pair that replaces residues 23 and 29. Following cleavage and global deprotection of peptide **1h**, the disulfide bridge across residues 15 and 37 is formed as described in step *e*. The crude single-disulfide and Cys(Acm) protected peptide **1h** is then isolated as described in step *f*.

The crude peptide **1h** is then dissolved in a solution of 0.1 M iodine in glacial acetic acid (15 mL), 50% aq. acetic acid (250 mL), and 1 M HCl (25 mL) to remove the Acm protecting groups and oxidize the cysteines to form a disulfide bridge across residues 23 and 29. The solution was concentrated by rotary evaporation to a volume less than 20 mL and purified as described in step *f*, to give peptide **1h** as a TFA salt.

Synthesis of peptides **2a–d**

The synthesis of peptides **2a–d** generally followed the same procedures as for peptides **1a–i**, except for the following:

a. Loading the resin. For peptides **2a–d**, 2-chlorotrityl chloride resin (300 mg, 1.4 mmol/g) was added to a Bio-RAD Poly-Prep chromatography column (10 mL). Dry CH_2Cl_2 (8 mL) was used to suspend and swell the resin for 30 min with gentle rocking. The solution was drained from the resin and a solution of Boc-Orn-Fmoc-OH (0.78 equiv, 150 mg, 0.33 mmol) in 6% (v/v) 2,4,6-collidine in dry CH_2Cl_2 (8 mL) was added immediately and the suspension was gently rocked for 12 h. The solution was then drained and a mixture of CH_2Cl_2/MeOH/*N*,*N*-diisopropylethylamine (DIPEA) (17:2:1, 10 mL) was added immediately. The resin was gently rocked for 1 h, to cap the unreacted 2- chlorotrityl chloride resin sites. The resin was then washed twice with dry CH_2Cl_2 and dried by passing nitrogen through the vessel. This procedure typically yields 0.18 mmol of loaded resin (0.6 mmol/g loading).

b. Manual peptide coupling. The resin loaded with Boc-Orn(Fmoc) was suspended in dry DMF and then transferred to a solid-phase peptide synthesis vessel. Residues 40 through 32 were manually coupled using Fmoc-protected amino acid building blocks. Each manual coupling cycle consisted of *i*. Fmoc-deprotection with of 20% (v/v) piperidine in DMF for 5 min at ambient

temperature (5 mL), *ii*. washing with dry DMF (2x, 5 mL), *iii*. coupling of the amino acid (0.44 mmol, 4 equiv) with HCTU (174.0 mg, 0.44 mmol, 4 equiv) in 20% (v/v) 2,4,6-collidine in dry DMF (5 mL) for 30 min, and *iv.* washing the with dry DMF (2x, 5 mL). Residue 32, which follows N-Me-Gly_{33}, was double coupled (4 equiv per coupling) using HATU (6 equiv) and HOAt (6 equiv) for 1 hr per coupling to ensure complete reaction. (We have found that coupling after N-methyl amino acids is difficult and requires rigorous coupling to minimize incomplete reaction.[2])

c. Microwave-assisted coupling of residues 31 through 12. A CEM Liberty Blue Automated Microwave Peptide Synthesizer was used to couple residues 31 to 12. Fmoc-Orn(Dde)-OH replaces residues 23–29 in the natural sequence. Each coupling cycle consisted of *i.* Fmoc-deprotection with 20% (v/v) piperidine with 0.1 M Oxyma Pure in DMF for 2 min. at 50 ºC, *ii.* washing with DMF (3x), *iii*. coupling of the amino acid (0.75 mmol, 5 equiv) in the presence of HCTU (0.675 mmol, 4.5 equiv) and 20% (v/v/) *N*-methylmorpholine (NMM) in DMF for 10 min. at 50 ºC, iv. washing with DMF (3x).

e. Cleavage of the peptide from resin. The resin was then transferred to a 10-mL Bio-Rad Poly-Prep chromatography column, and washed 3x with dry CH_2Cl_2. The acyclic peptide was cleaved from the resin by rocking the resin for 1 h with a solution of 1,1,1,3,3,3-hexafluoroispropanol (HFIP) in CH_2Cl_2 (1:4, 8 mL). The suspension was filtered and the filtrate was collected in a 250-mL round-bottomed flask. The resin was washed with additional HFIP in CH_2Cl_2 (1:4, 8 mL). The combined filtrates were concentrated by rotary evaporation to give a white solid. The white solid was further dried under vacuum to afford the crude protected linear peptide, which was cyclized without further purification.

f. Cyclization of the acyclic peptide. The crude protected linear peptide was dissolved in dry DMF (150 mL). PyBOP (370 mg, 0.711 mmol, 6 equiv) and *N*-methylmorpholine (NMM) (0.33 mL, 1.8 mmol, 12 equiv) was added to the solution and the mixture was stirred under nitrogen for 48 h. The mixture was concentrated under reduced pressure to afford the crude protected cyclic peptide, a yellow film.

e. *Global deprotection of the cyclic peptide.* The protected cyclic peptide was dissolved in TFA/triisopropylsilane (TIPS)/H_2O (18:1:1, 10 mL) in a 250-mL round-bottomed flask equipped with a nitrogen-inlet adaptor. The solution was stirred for 1 h under nitrogen. The reaction mixture was then concentrated by rotary evaporation under reduced pressure to afford the crude cyclic peptide. During the 1 h deprotection, two 50-mL conical tubes containing 40-mL of dry ether were chilled on dry ice. The solution was drained and split between the two conical tubes of ether. The tubes were then centrifuged at 800 x g for 25 min. The ether supernatant was poured off and the pelleted peptide was dried overnight under vacuum.

e. Oxidation to form the disulfide bridge. The crude, pelleted peptide was resuspended in a solution of 20% v/v dimethyl sulfoxide (DMSO), 50 mL in each tube. The suspension was gently rocked at room temperature for 48 h. The reaction mixture was concentrated under reduced pressure until only DMSO remained.

g. Reverse-phase HPLC purification. The peptide: DMSO mixture was dissolved in H_2O and acetonitrile (8:2, 5 mL), and the solution was filtered through a 0.2 μm syringe filter and purified by RP-HPLC. The solution of crude peptide was injected at 20% acetonitrile and eluted with a gradient of 20-60% CH_3CN over 90 min. Each peptide eluted between 29-36% CH_3CN. The collected fractions were analyzed by analytical HPLC and MALDI-TOF, and the pure fractions

were concentrated by rotary evaporation and lyophilized. Typical syntheses yielded between 8 and 38 mg of the peptide as the TFA salt.

SDS-PAGE, TCEP reduction, and silver staining

SDS-PAGE was performed on peptides **1a–i** and **2a–d** using the reagents, recipes, and procedures for Tricine SDS-PAGE detailed in Schägger, H. *Nat. Protoc.* **2006**, *1*, 16–22. Each peptide was run on a 16% polyacrylamide gel with a 4% stacking polyacrylamide gel at 60 volts. A Spectra™ Multicolor Low Range Protein Ladder (ThermoFischer Scientific, catalog #: 26628) was loaded into the first lane of the gel. The remaining lanes were loaded with 8.0 µL aliquots of each peptide as 50 µM solutions in SDS-PAGE loading buffer, which were prepared as follows: A 10 mg/mL stock solution of each peptide was prepared with deionized water. Aliquots of the 10 mg/mL solutions were then diluted further with deionized water and 6X SDS-PAGE loading buffer (G-Biosciences catalog #: 786-701) to create 100 µM working solutions of each peptide.

To reduce the disulfide bonds present in peptides **1a**, **2a**, and **2b** 50 µM solutions of each peptide were sonicated for 1 h in 10mM TCEP prior to gel loading. After 1 h had elapsed, the solution was diluted further with 6X SDS-PAGE loading buffer (G-Biosciences catalog #: 786-701) to create 100 µM working solutions of each peptide.

Staining with silver nitrate was used to visualize peptides **1a–i** and **2a–d** in the SDS-PAGE gel. Reagents for silver staining were prepared according to procedures detailed in Simpson, R. J. *Cold Spring Harbor Protocol* **2007**. [We have found it important to prepare sodium thiosulfate, silver nitrate, and developing solutions fresh each time and to use high purity sodium carbonate to prepare the developing solution.] The gel was removed from the casting glass and rocked for 20 min in fixing solution of 50% (v/v) methanol and 5% (v/v) acetic acid in deionized water. The fixing solution was then discarded and replaced with 50% (v/v) aqueous methanol for another 10 min of rocking. Next, the 50% methanol was discarded and replaced with deionized water for another 10 min of rocking. Next, the water was discarded and the gel was rocked in 0.02% (w/v) sodium thiosulfate in deionized water for 1 min. The sodium thiosulfate was discarded and the gel

was rinsed twice with deionized water for 0.5 min. The gel was then submerged in pre-chilled 0.1% (w/v) silver nitrate in deionized water and rocked at 4 ºC for 20 min. The silver nitrate solution was discarded and the gel was rinsed twice with deionized water. The gel was incubated in developing solution (2% (w/v) sodium carbonate, 0.04% (w/v) formaldehyde) until the solution began to brown. The developing solution was then immediately discarded and fresh silver nitrate solution was added to the gel until the desired intensity of staining was reached. When the desired intensity of staining was reached, the developing solution was discarded and the gel was submerged in 5% aqueous acetic acid.

Circular dichroism spectroscopy

A 50 µM solution of each peptide was prepared by diluting the 10 mg/mL stock solution with 10 mM sodium phosphate buffer at pH 7.4. Each solution was transferred to a 1 mm quartz cuvette for data acquisition. Circular dichroism spectra were acquired on a Jasco J-810 circular dichroism spectropolarimeter at ambient temperature. Data were collected using 2.0 nm intervals from 260 nm to 190 nm and averaged over five accumulations with 7-point smoothing.

*Crystallization of peptide **2a***

The hanging-drop vapor-diffusion method was used to determine initial crystallization conditions for peptide **2a**. Peptide **2a** was screened in 96-well plate format using three crystallization kits (Crystal Screen, Index, and PEG/ION) from Hampton Research. A TTP LabTech Mosquito liquid handling robot was used to make three 150 nL hanging drops for each well condition. The three hanging drops differed in the ratio of peptide to well solution for each condition in the 96-well plate. A 10 mg/mL solution of each peptide in deionized water was combined with a well solution in ratios of 1:1, 1:2, and 2:1 peptide:well solution at appropriate volumes to create the three 150 nL hanging drops. Crystals of peptide **2a** grew in well conditions

of 0.2 M magnesium acetate tetrahydrate, 0.1 M sodium cacodylate trihydrate pH 6.5, and 30% (v/v) 2-methyl-2,4-pentanediol.

Crystallization conditions for each peptide were optimized using a 4x6 matrix Hampton 24-well plate. For peptide **2a** the 0.1 M sodium cacodylate trihydrate buffer was varied in each row in increments of 0.5 pH units (6.0, 7.5, 7.0, and 7.5) and the percentage of isopropyl alcohol in each column in increments of 2% (v/v) (26%, 28%, 30%, 32%, 34%, 36%). Three hanging-drops were prepared on borosilicate glass slides by combing a 10 mg/mL solution of peptide **2a** in deionized water with the well solution in the following amounts: 1 μL:1 μL, 2 μL:1 μL, and 1 μL:2 μL. Slides were inverted and pressed firmly against the silicone grease surrounding each well. Crystals were harvested with a nylon loop attached to a copper or steel pin, soaked briefly in MPD, and flash frozen in liquid nitrogen prior to data collection. The optimized crystallization conditions for peptide **2a** are summarized in Supplementary Table 1.

*X-ray crystallographic data collection, data processing, and structure determination of peptide **2a***

Diffraction data for **2a** were collected at the Stanford Synchrotron Radiation Light source, beamline 12.2, at 1.00 Å wavelength. Datasets were indexed and integrated with XDS.[2] Scaling and merging was done with pointless and aimless in CCP4.[3] The structures were solved with molecular replacement in Phaser[4] using a dimer formed by macrocyclic β-hairpin peptide derived from $A\beta_{16\text{–}36}$ (PDB 6WXM) as the search model. The refinement was done with phenix.refine module of the Phenix suite, with manipulation of the model performed using Coot.[6]

Replica-exchange molecular dynamics (REMD) simulation of an $A\beta_{9\text{–}42}$ β-barrel-like tetramer

A model of an $A\beta_{9\text{–}42}$ β-barrel-like tetramer was generated by replica-exchange molecular dynamics as follows: Starting coordinates for $A\beta_{9\text{–}42}$ were generated from the crystallographic coordinates of peptide **2a**. Symmetry mates of peptide **2a** were generated using PyMOL.[7] The

delta-linked ornithine ($^{\delta}$Orn) residues were deleted from each macrocycle. Three alanine residues were added to the N-terminus of the β-hairpin, and two alanine residues were added to the C-terminus. Seven alanine residues were added to connect Glu_{22} and Ala_{30}. The configuration of these added residues were optimized in PyMOL using the clean function, ensuring that the crystallographic coordinates of $A\beta_{12–22}$ and $A\beta_{30–40}$ were not perturbed. After the energy minimization, each added alanine was mutated to its corresponding wild-type residue from Aβ. The configuration of the mutated residues were again optimized in PyMOL using the clean function. Residues of native Aβ were then introduced at positions 33, 15, and 37 as follows: Each N-Me-Gly_{33} residue was replaced with Gly. Each Cys_{15} residue was replaced with Gln. Each Cys_{37} residue was replaced with Gly.

The autopsf plugin in VMD[8] was used to prepare the required files for simulation. The coordinates for $A\beta_{12–22}$ and $A\beta_{30–40}$ were fixed throughout the simulation. REMD simulations were run using NAMD 2.14[9] with the CHARMM22[10,11] force field and generalized Born implicit solvent (GBIS) on 32 replicas. The temperatures for these replicas varied between 300 and 800 K. The simulation was performed for 10 ns. Representative coordinates were selected uniformly from the last 9 ns of the simulation.

Molecular Dynamics Simulation of an $A\beta_{9–42}$ β-barrel-like tetramer in a lipid membrane

The CHARMM-GUI input generator was used to set up a β-barrel like tetramer formed by $A\beta_{9–42}$ embedded in a POPC lipid bilayer in excess water. The tetramer was oriented so that the axis of the pore-like opening was parallel to the membrane bilayer normal. The protonation states of titratable amino acids were chosen for a corresponding system pH of 7.4. The simulation system consisted of one $A\beta_{9–42}$ tetramer, 200 POPC lipids (100 per leaflet), 9049 waters, and 4 positive counterions, for a total of 55975 atoms and an initial simulation cell size of 87.8 Å x 87.8 Å x 80.0

Å. The simulation was carried out for 500 ns at constant temperature (300 K) and constant pressure (1 atm) using NAMD 2.14[9]. The CHARMM36m[10] and CHARMM36[11] forcefield were used to model the protein and lipids, respectively. The TIP3P model was used to model the water molecules.[12]

The initial equilibration of the system consisted of 10000 steps of energy minimization followed by six 100-ps runs with harmonic restraints applied to all the protein heavy atoms with decreasing force constants. a conjugate gradient algorithm. Once the pre-equilibration procedure was completed, the unrestrained system was run for 500 ns. A reversible multiple time-step algorithm[13] was used to integrate the equations of motion with time steps of 2 fs for the short-range non-bonded forces and bonded forces, and 4 fs for the long-range electrostatic interactions. These last interactions were computed using the smooth particle mesh Ewald summation.[14,15] The short-range interactions were cut off at 12 Å using a forced-based switching function between 10 and 12 Å. The SHAKE algorithm[16] was used to constrain the length of all bonds involving hydrogen atoms. The temperature was maintained at 300 K using a Langevin dynamics scheme, and a Nosé-Hoover Langevin piston was to keep the pressure constant at 1 atm.[17, 18] Molecular graphics and simulation analyses were generated with VMD 1.9.3.[8]

Caspase-3/7 activation assay

The toxicity of peptides **1a**, **1h**, **2a**, and **2d** toward SH-SY5Y cells was assessed by a caspase-3/7 activation assay. SH-SY5Y cells were plated in a 96-well plate at 30,000 cells per well. Cells were incubated in 100 μL of a 1:1 mixture of DMEM:F12 media supplemented with 10% fetal bovine serum, 100 U/ml penicillin, and 100 μg/mL streptomycin at 37°C in a 5% CO_2 atmosphere and allowed to adhere to the bottom of the plate for 24 hours. After 24 hours and prior to treatment with peptide, the media was replaced with serum and antibiotic free DMEM:F12.

Cells were incubated in the presence or absence of each peptide at concentrations of 6.25, 12.5, 25, 50 and 100 μM for 48 h in 96-well plates. After 43 h, 5 μM of staurosporine was added to the positive control wells. The caspase-3/7 assay was performed using the Promega Apo-One Homogeneous Caspase-3/7 Assay. Experiments were performed in replicates of 3, and an additional 6 wells were used for the controls. After 48 h, the Apo-one Reagent was added, and the plates were allowed to incubate for an additional 4 h at room temperature. The fluorescence of each well was measured with an excitation at 498 nm and emission at 523 nm using a Promega GloMax Discover System.

Dye-Leakage Assay

Chicken egg-derived L-α-phosphatidylcholine (PC, product number: 840051C) and porcine brain-derived L-α-phosphatidylserine (PS, product number: 840032) were purchased from Avanti Polar Lipids as 10 mg/mL solutions in chloroform. Liposomes were prepared using 2.6 μmols of lipids as a 1:1 molar ratio of PC and PS. A solution of 2.6 μmols lipid in chloroform was placed into a 10 x 75 mm disposable culture tube. Chloroform was removed under a stream of dry N_2 gas to yield a lipid film. The culture tube was put under vacuum (< 5 mTorr) for ca. 12 h to ensure complete removal of chloroform from the lipid film.

To prepare calcein-encapsulated LUVs, the lipids were resuspended in 500 μL of a 103 mM calcein solution (70 mg calcein dissolved in 865 μL 18 MΩ deionized H_2O and 135 μL 2 N NaOH). If calcein was not dissolved, then 2 N NaOH was added dropwise until complete dissolution was achieved. The lipids were allowed to rehydrate over 1 h and vortexed every 10 mins to ensure all lipids fell into solution before being extruded 21 times through 100 nm filters. The LUVs were then separated from free calcein by passage through a 10 x 1 cm column of Sephadex G-50 using leakage buffer (10 mM Tris pH 7.4, 150 mM NaCl and 1 mM EDTA). LUVs

were collected as the yellow-orange fractions that did not fluoresce under long-wave UV light, eluting before the free calcein dye.

Concentration of the lipids was determined using a modified phosphorus assay, as follows. To a 10 x 75 mm disposable culture tube was added 50 μL of the LUV suspension and to another culture tube was added 50 μL of leakage buffer as a control (both run in triplicate). Then 30 μL of a 10% (w/v) solution of $Mg(NO_3)_2$ in ethanol was added to the culture tubes. This mixture was ashed over a hot flame resulting in the formation of a grey precipitate. To dissolve the precipitate, 300 μL of 0.5 M HCl was added to the tubes. This solution was heated for 15 mins in a boiling water bath. After cooling to room temperature, 700 μL of a mixture of 1% (w/v) ascorbic acid and 0.378% (w/v) ammonium molybdate tetrahydrate dissolved in 0.45 M H_2SO_4 was added to the boiled solutions. This mixture was heated for 1 h at 37 °C. Over this time, the solutions with the LUVs developed a darker blue color compared to the control tubes. Absorbance of each solution was then measured in a 1 cm quartz cuvette at 820 nm. The concentration of phosphate was determined using a molar extinction coefficient of 120 M^{-1} cm^{-1}. The concentration of total lipid is assumed to be equal to the concentration of phosphate measure in this assay, as one mole of phospholipid contains one mole of phosphate.

The stock LUV suspension was diluted in leakage buffer to a final concentration of 11 μM lipid. Solutions of peptides were prepared gravimetrically by dissolving the lyophilized peptide in the appropriate amount of 18 MΩ deionized H_2O to achieve a 10 mg/mL stock. From the 10 mg/mL stock solutions, 10X solutions were made by dilution with 18 MΩ deionized H_2O. From these 10X stock solutions, 10 μL were added to the wells of the 96-well plate in triplicate. An additional 8 wells were used as controls. Four wells received 10 μL of 18 MΩ deionized H_2O (vehicle, negative control) and the other four wells received 10 μL of 10X lysis buffer (positive

control). To every well was added 190 μL of the 11 μM LUV suspension. The fluorescence was immediately recorded on a Thermo Scientific Varioskan Lux fluorescent plate reader. The excitation wavelength was set to 490 nm and emission was recorded at 520 nm. Data was averaged across the three replicate wells. Data is plotted using the below equation:

$$\% \text{ dye leakage} = [(F_{peptide} - F_{water})/(F_{lysis} - F_{water})] \times 100$$

Where $F_{peptide}$ is the average fluorescence of the given peptide treatments, F_{water} is the average fluorescence of the water treatments, and F_{lysis} is the average fluorescence of the lysis buffer treatments.

Dynamic light scattering (DLS)

A Malvern Zetasizer ZS Nano DLS was used to measure the dynamic light scattering of peptides **1a**, **2a**, and **2b**. 70 μM solutions of each peptide were prepared in 10 mM potassium phosphate buffer at pH 7.4 and transferred to a 1 cm disposable plastic cuvette. Data were collected in 10 second time intervals over 3 measurements, at ambient room temperature for each peptide. The scatter was measured with a 173° backscattering angle.

References

1. These procedures follow closely those that our laboratory has previously published. The procedures in this section are adapted from and in some cases taken verbatim from Kreutzer, A. G.; Hamza, I. L.; Spencer, R. K.; Nowick J. S. *J. Am. Chem. Soc.* **2016**, *138*, 4634–4642, Spencer, R. K.; Kreutzer, A. G.; Salveson, P. J.; Li, H.; Nowick, J. S. *J. Am. Chem. Soc.* **2015**, *137*, 6304–6311, Spencer, R. K.; Li. H.; Nowick, J. S. *J. Am. Chem. Soc.* **2014**, *136*, 5595– 5598, and Kreutzer, A. G.; Yoo, S.; Spencer, R. K.; Nowick, J. S. *J. Am. Chem, Soc.* **2017**, *139*, 966–975, Samdin, T. D.; Wierzbicki, M.; Kreutzer, A. G.; Howitz, W. J.; Valenzuela, M.; Smith, A.; Sahrai, V.; Truex, N. L.; Klun, M.; Nowick, J. S. *J. Am. Chem. Soc*. **2020**, *142*, 11593–11601; Howitz, W. J.; Guaglianone, G.; McKnelly K. J.; Haduong, K.; Ashby, S. N.; Laayouni, M.; Nowick, J. S. *ACS Chem. Neurosci.* **2022**, *13*, 6, 714–720.
2. Kabsch, W. *Acta Crystallogr., Sect. D: Biol. Crystallogr.* **2010**, *66*, 125–132.
3. Winn, M. D.; Ballard, C. C.; Cowtan, K. D.; Dodson, E. J.; Emsley, P.; Evans, P. R.; Keegan, R. M.; Krissinel, E. B.; Leslie, A. G. W.; McCoy, A.; McNicholas, S. J.; Murshudov, G. N.; Pannu, N. S.; Potterton, E. A.; Powell, H. R.; Read, R. J.; Vagin, A.; Wilson, K. S. *Acta Crystallogr., Sect. D: Biol Crystallogr.* **2011**, *67*, 235–242.
4. McCoy, A. J.; Grosse-Kunstleve, R. W.; Adams, P. D.; Winn, M. D.; Storoni, L. C.; Read, R. J. J. *Appl. Crystallogr*. **2007**, *40*, 658-674.
5. Adams, P. D.; Afonine, P. V.; Bunkoczi, G.; Chen, V. B.; Davis, I. W.; Echols, N.; Headd, J. J.; Hung, L. W.; Kapral, G. J.; Grosse-Kunstleve, R. W.; McCoy, A. J.; Moriarty, N. W.; Oeffner, R.; Read, R. J.; Richardson, D. C.; Richardson, J. S.;

Terwilliger, T. C.; Zwart, P. H. *Acta Crystallogr., Sect. D: Biol. Crystallogr.* **2010**, *66*, 213–221

6. Emsley, P.; Lohkamp, B.; Scott, W. G.; Cowtan, K. *Acta Crystallogr., Sect. D: Biol. Crystallogr.* **2010**, *66*, 486–501
7. The PyMOL Molecular Graphics System, Version 2.4.0 Schrödinger, LLC.
8. Humphrey, W., Dalke, A. & Schulten, K. VMD: Visual Molecular Dynamics. *J. Mol. Graph.* **14**, 33–38 (1996).
9. Phillips, J. C.; Hardy, D. J.; Maia, J. D. C.; Stone, J. E.; Ribeiro, J. V.; Bernardi, R. C.; Buch, R.; Fiorin, G.; Hénin, J.; Jiang, W.; McGreevy, R.; Melo, M. C. R.; Radak, B. K.; Skeel, R. D.; Singharoy, A.; Wang, Y.; Roux, B.; Aksimentiev, A.; Luthey-Schulten, Z.; Kalé, L. V.; Schulten, K.; Chipot, C.; Tajkhorshid, E. Scalable Molecular Dynamics on CPU and GPU Architectures with NAMD. *J. Chem. Phys.* **2020**, *153* (4).
10. Huang, J.; Rauscher, S.; Nawrocki, G.; Ran, T.; Feig, M.; De Groot, B. L.; Grubmüller, H.; MacKerell, A. D. CHARMM36m: An Improved Force Field for Folded and Intrinsically Disordered Proteins. *Nat. Methods* **2016**, *14* (1), 71–73.
11. Klauda, J. B.; Venable, R. M.; Freites, J. A.; O'Connor, J. W.; Tobias, D. J.; Mondragon-Ramirez, C.; Vorobyov, I.; MacKerell, A. D.; Pastor, R. W. Update of the CHARMM All-Atom Additive Force Field for Lipids: Validation on Six Lipid Types. *J. Phys. Chem. B* **2010**, *114* (23), 7830–7843.
12. Jorgensen, W. L., Chandrasekhar, J., Madura, J. D., Impey, R. W. & Klein, M. L. Comparison of simple potential functions for simulating liquid water. *J. Chem. Phys.* **79**, 926–935 (1983).

13. H. Grubmüller, H. Heller, A. Windemuth & K. Schulten Generalized Verlet. Algorithm for Efficient Molecular Dynamics Simulations with Long-range Interactions, *Molecular Simulation*, **6:1-3**, 121-142 (1991).

14. Darden, T., York, D. & Pedersen, L. Particle mesh Ewald: An N·log(N) method for Ewald sums in large systems. *J. Chem. Phys.* **98**, 10089–10092 (1993).

15. Essmann, U.; Perera, L.; Berkowitz, M. L.; Darden, T.; Lee, H.; Pedersen, L. G. A Smooth Particle Mesh Ewald Method. J. Chem. Phys. 1995, 103 (19), 8577–8593.

16. Ryckaert, J. P., Ciccotti, G. & Berendsen, H. J. C. Numerical integration of the cartesian equations of motion of a system with constraints: molecular dynamics of n-alkanes. *J. Comput. Phys.* **23**, 327–341 (1977).

17. Feller, S. E., Zhang, Y., Pastor, R. W. & Brooks, B. R. Constant pressure molecular dynamics simulation: The Langevin piston method. *J. Chem. Phys.* **103**, 4613–4621 (1995).

18. Martyna, G. J., Tobias, D. J. & Klein, M. L. Constant pressure molecular dynamics algorithms. *J. Chem. Phys.* **101**, 4177–4189 (1994).

Characterization Data

*Characterization of peptide **1a***

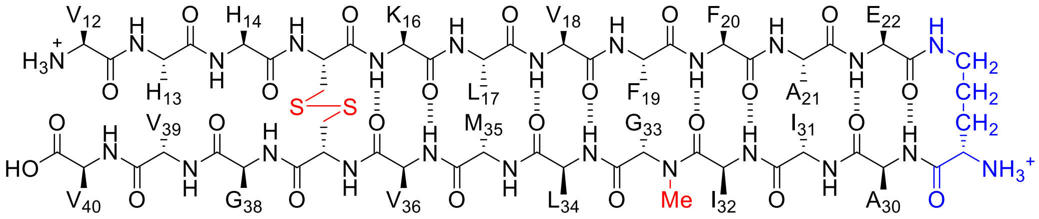

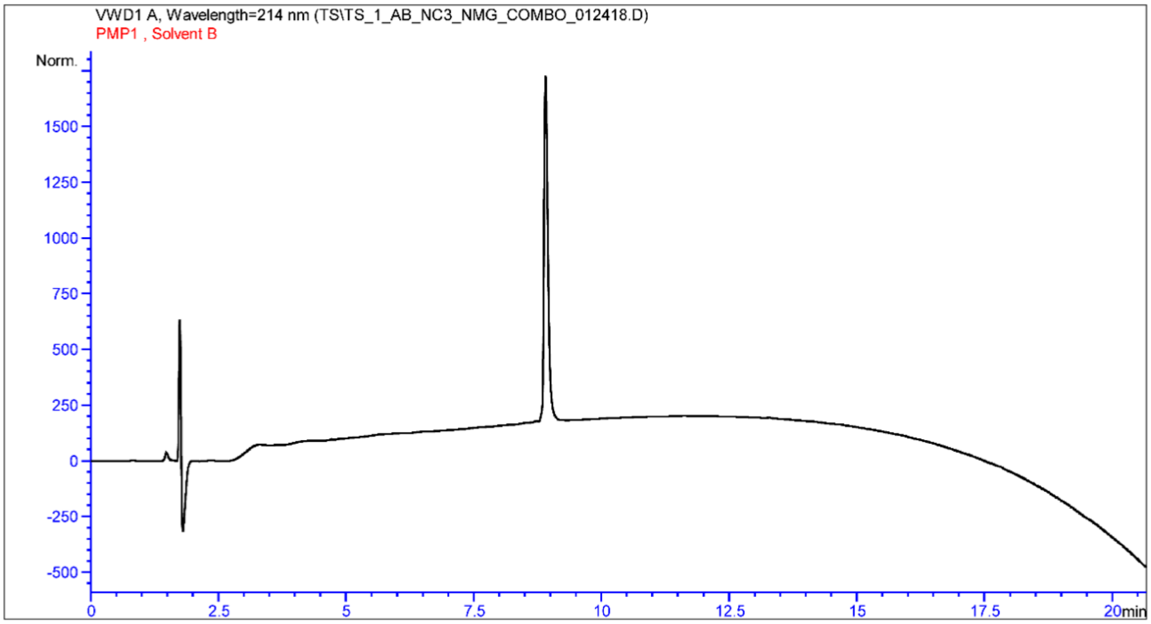

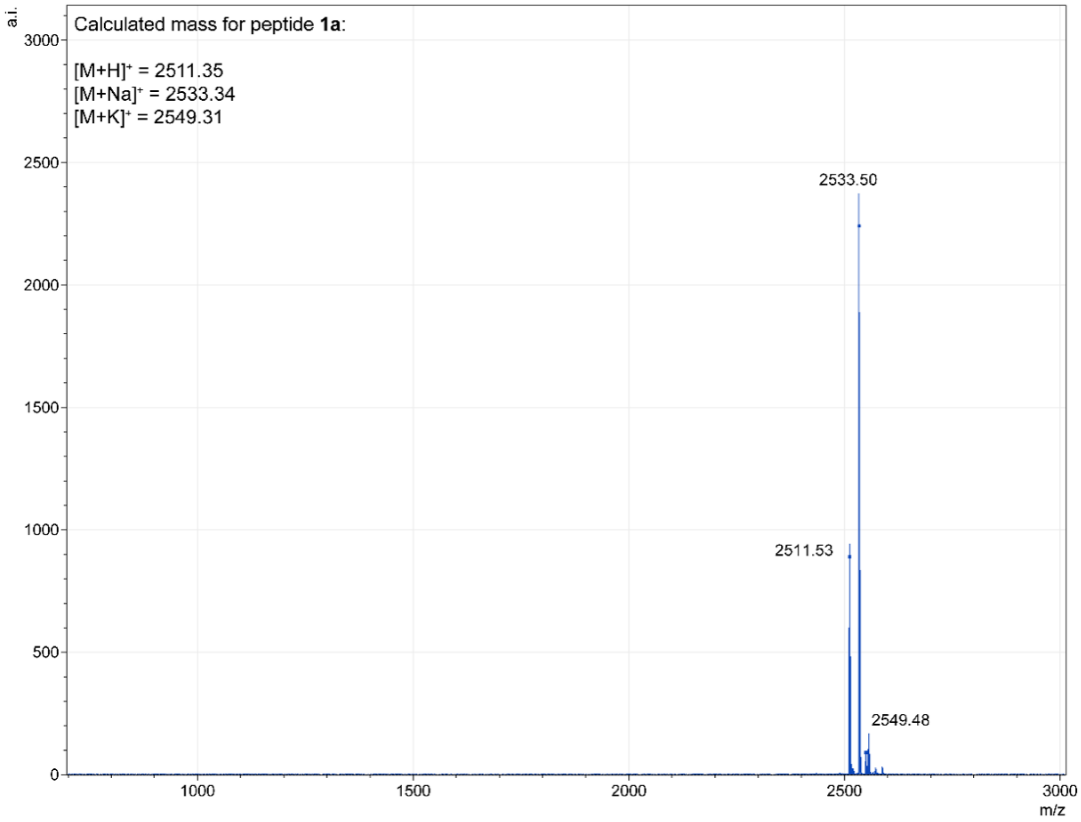

a.i.
Calculated mass for peptide **1a**:
[M+H]+ = 2511.35
[M+Na]+ = 2533.34
[M+K]+ = 2549.31
2533.50
2511.53
2549.48
3000
2500
2000
1500
1000
500
0
1000
1500
2000
2500
3000
m/z

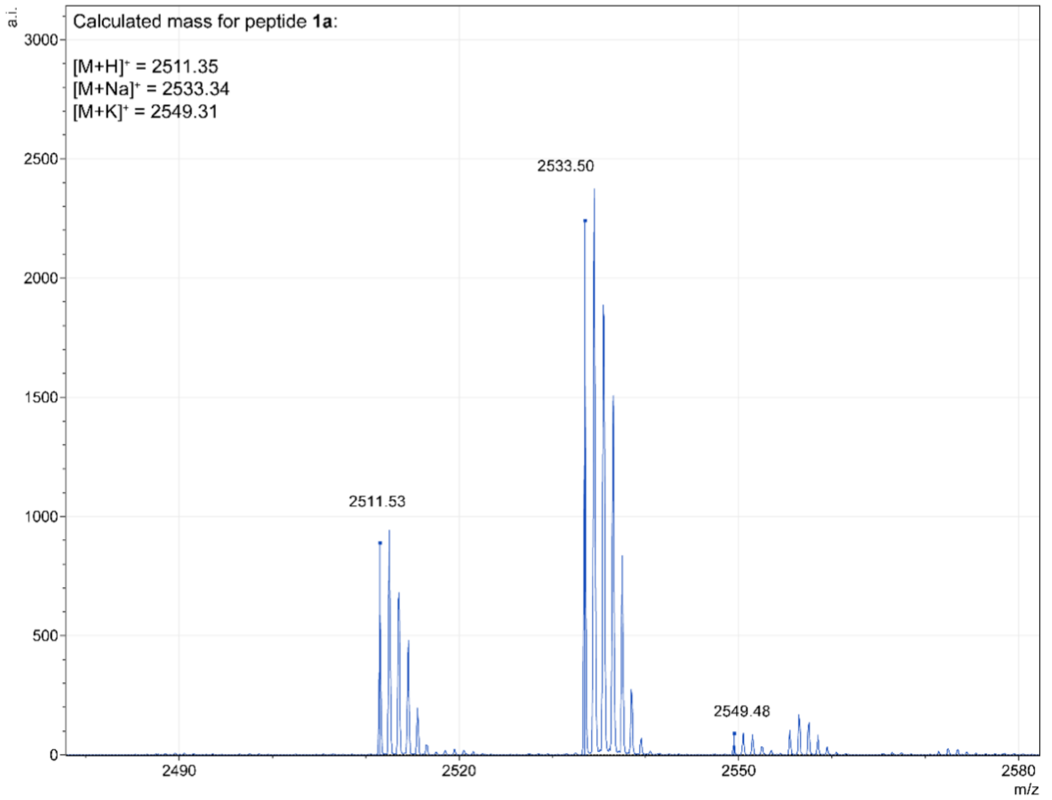

a.i.
Calculated mass for peptide **1a**:
[M+H]+ = 2511.35
[M+Na]+ = 2533.34
[M+K]+ = 2549.31
2533.50
2511.53
2549.48
3000
2500
2000
1500
1000
500
0
2490
2520
2550
2580
m/z

*Characterization of peptide **1b***

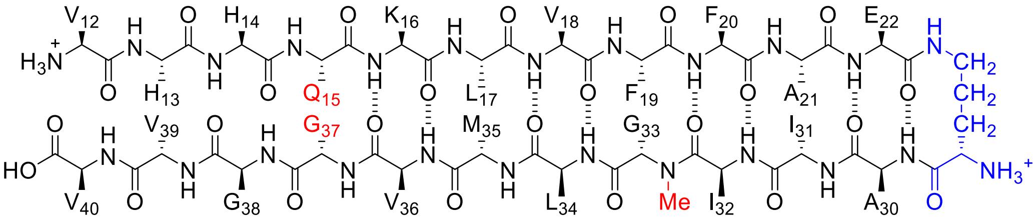

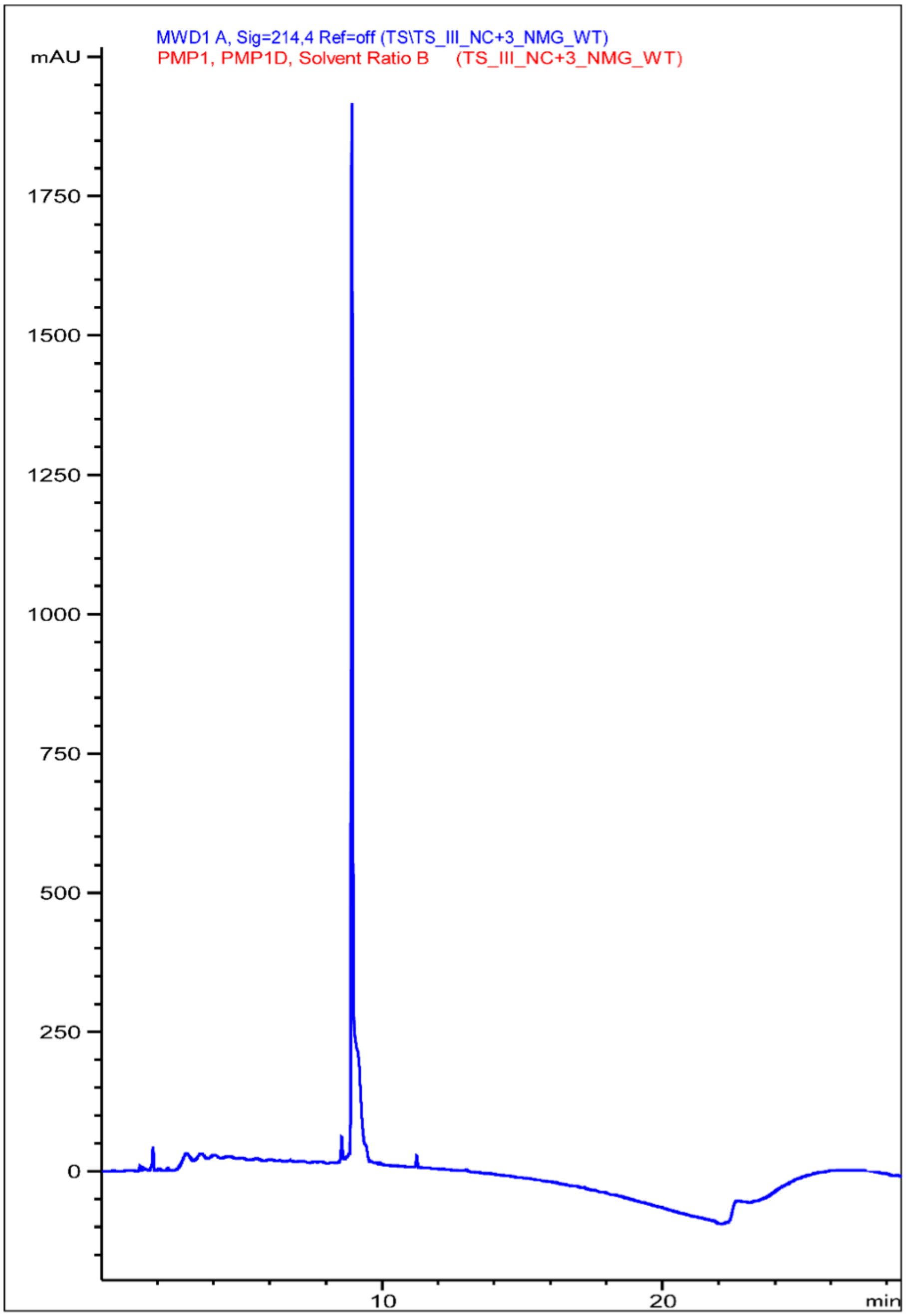

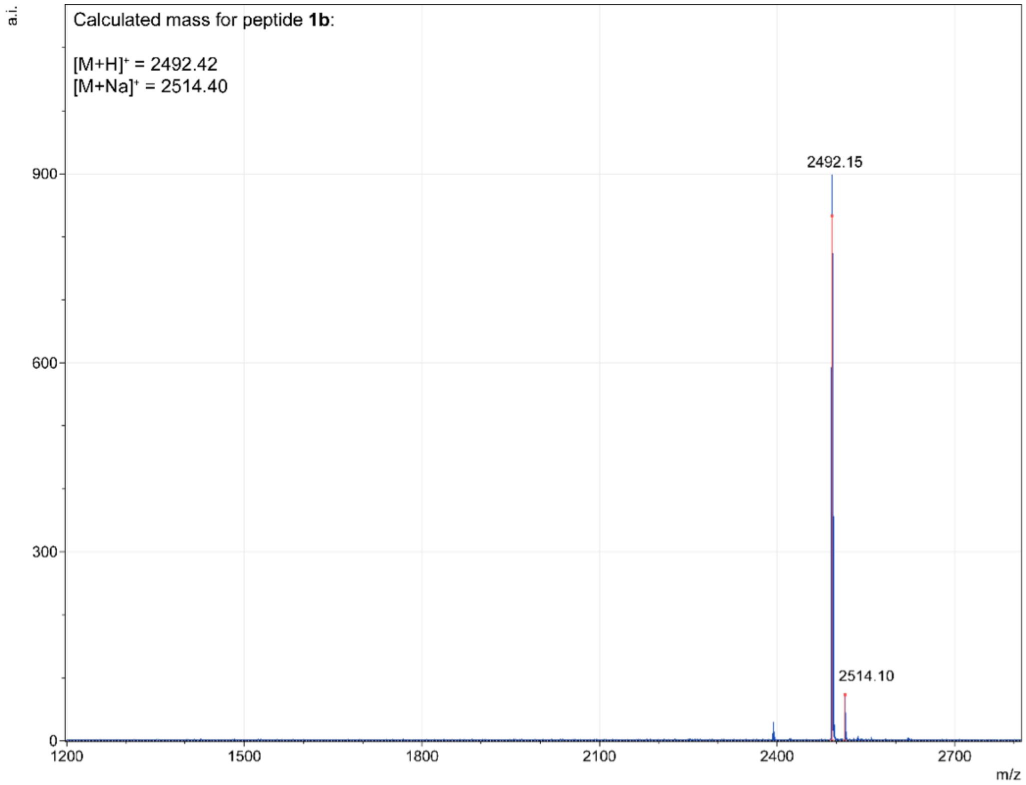
a.i.
Calculated mass for peptide **1b**:
[M+H]+ = 2492.42
[M+Na]+ = 2514.40
2492.15
2514.10
900
600
300
0
1200
1500
1800
2100
2400
2700
m/z

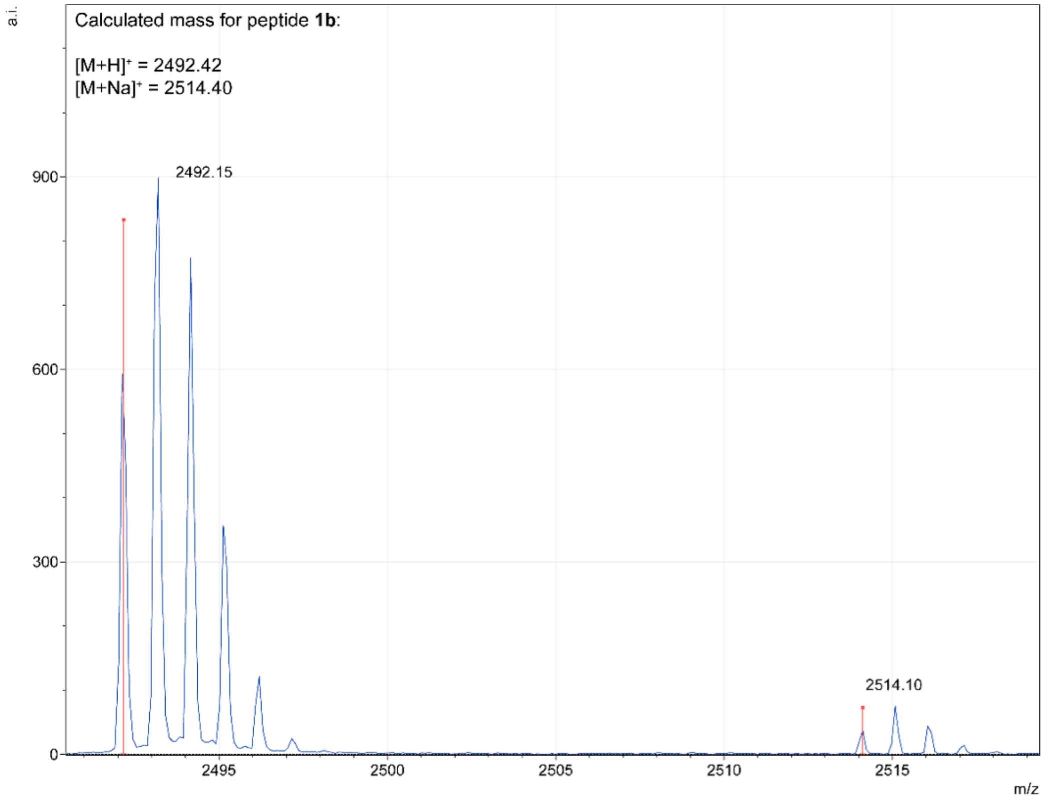
a.i.
Calculated mass for peptide **1b**:
[M+H]+ = 2492.42
[M+Na]+ = 2514.40
2492.15
2514.10
900
600
300
0
2495
2500
2505
2510
2515
m/z

*Characterization of peptide **1c***

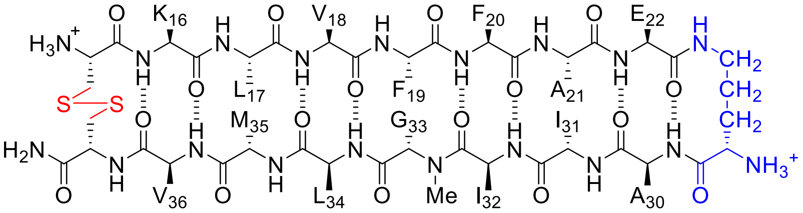

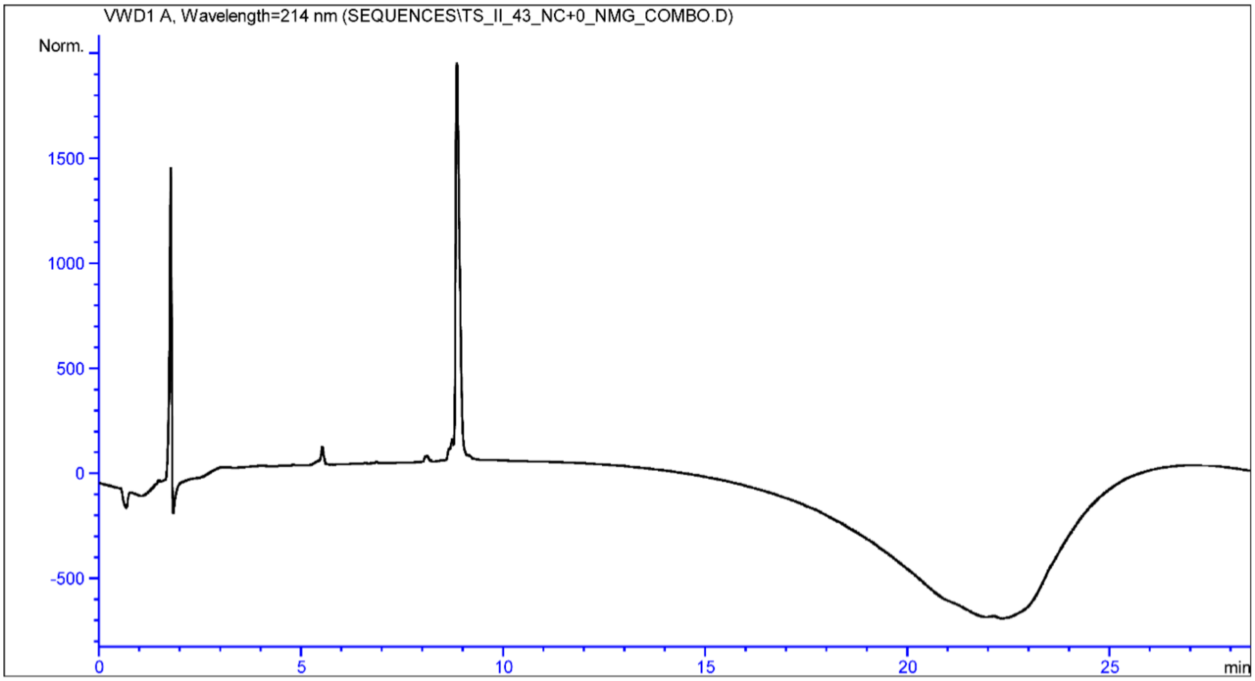

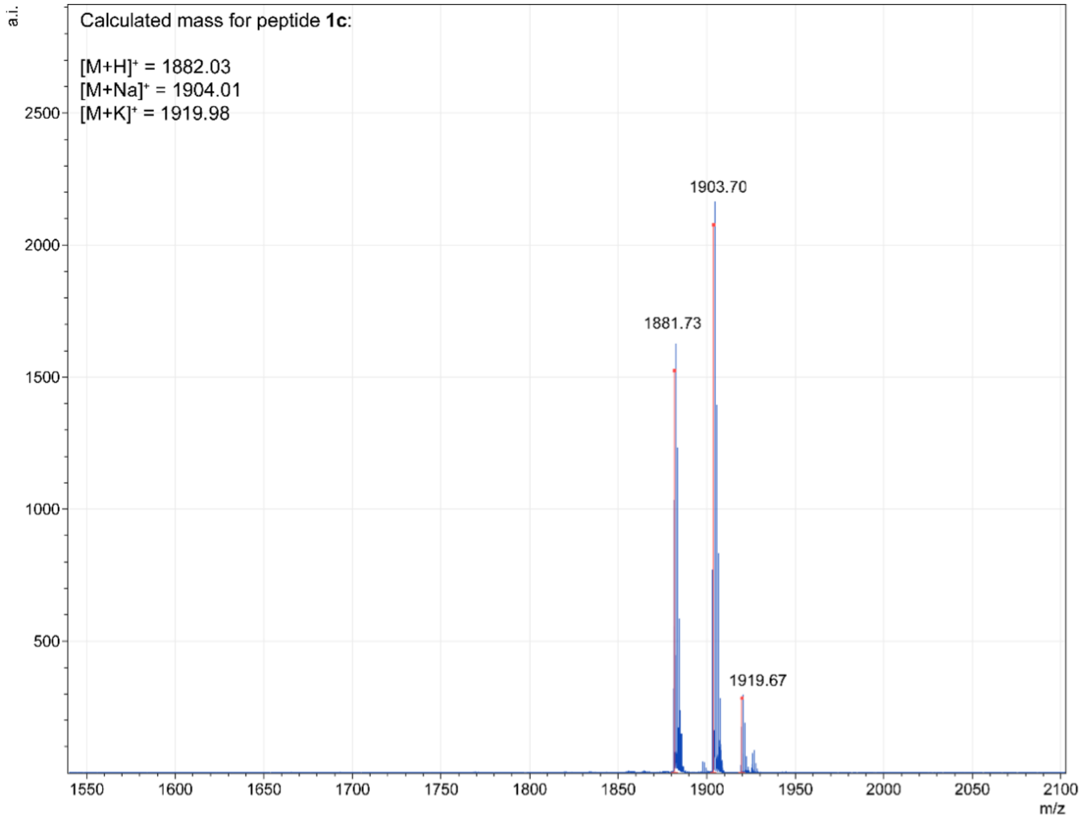
Calculated mass for peptide **1c**:
[M+H]+ = 1882.03
[M+Na]+ = 1904.01
[M+K]+ = 1919.98
1903.70
1881.73
1919.67
a.i.
m/z

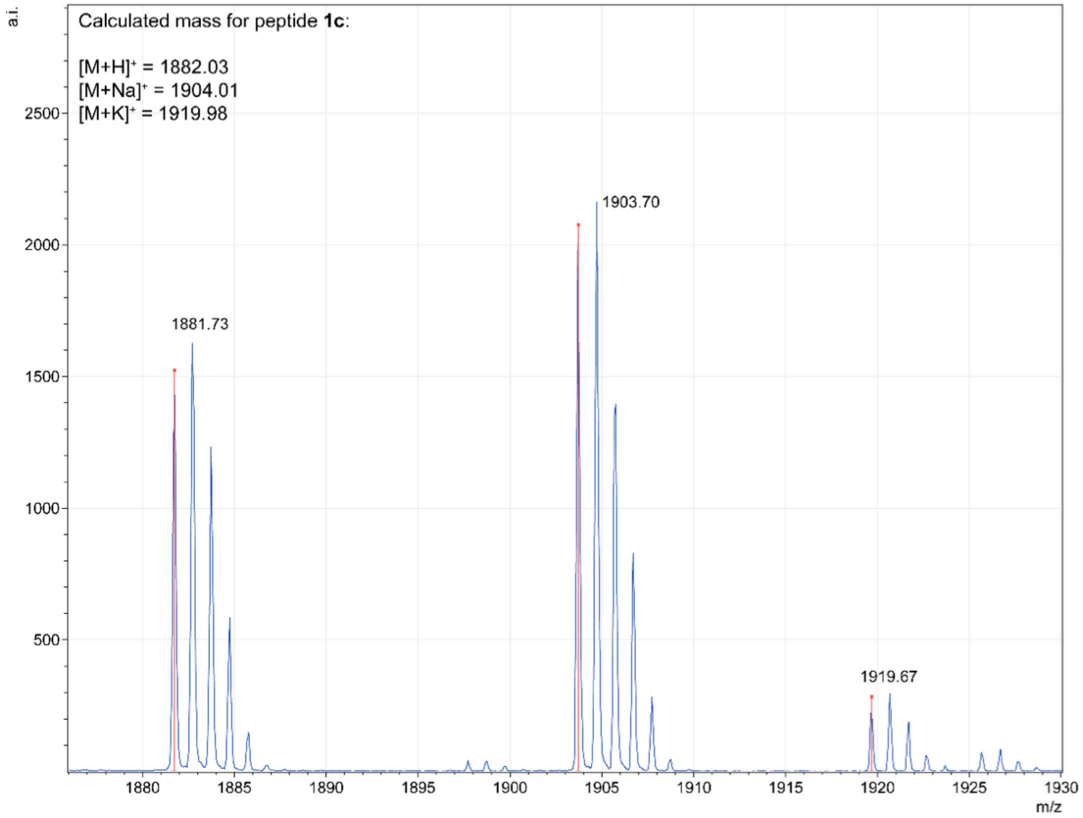
Calculated mass for peptide **1c**:
[M+H]+ = 1882.03
[M+Na]+ = 1904.01
[M+K]+ = 1919.98
1903.70
1881.73
1919.67
a.i.
m/z

*Characterization of peptide **1d***

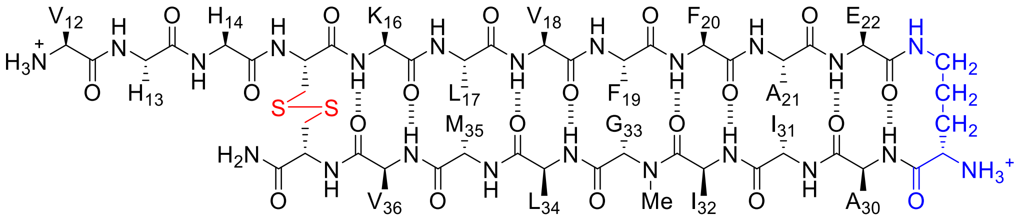

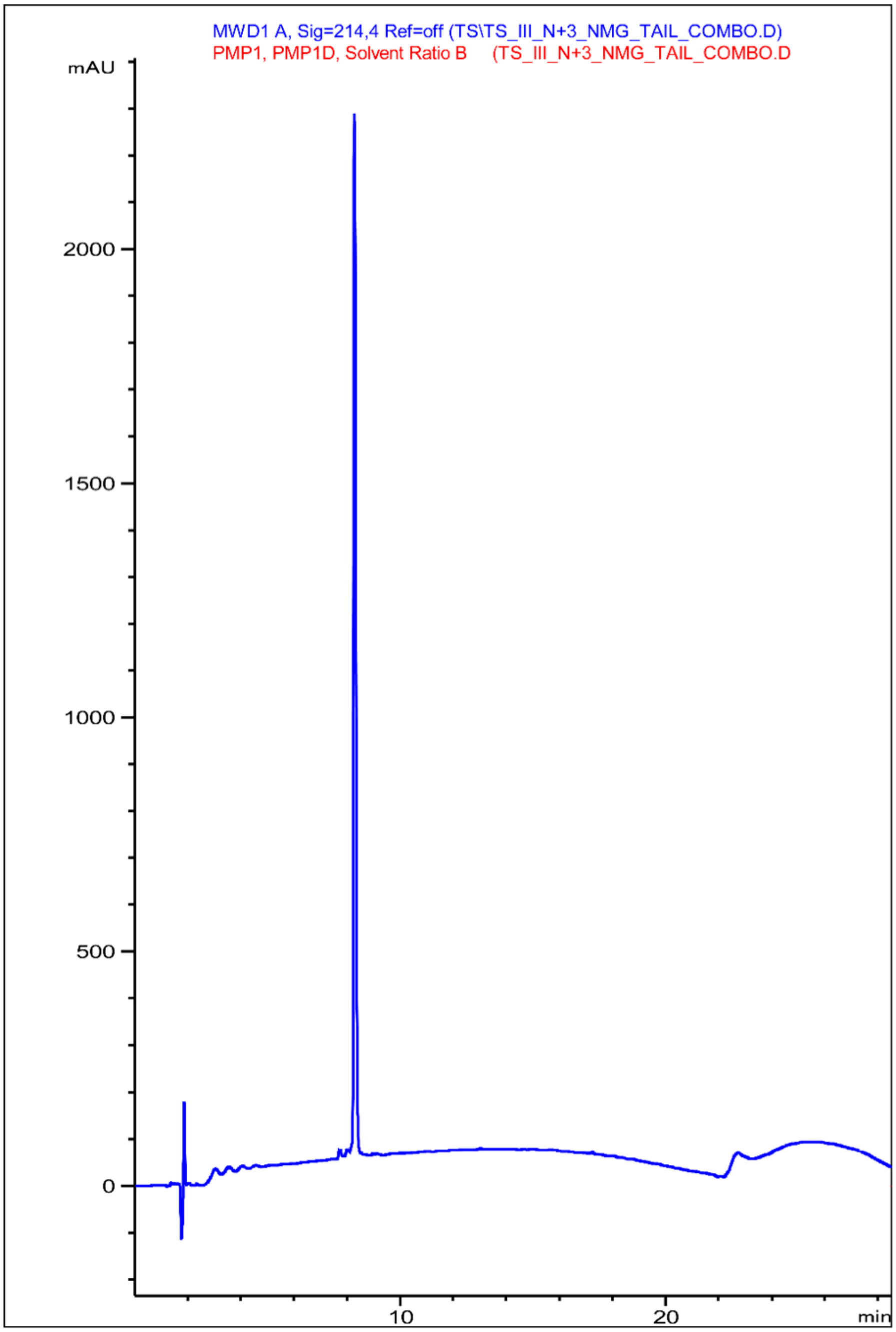

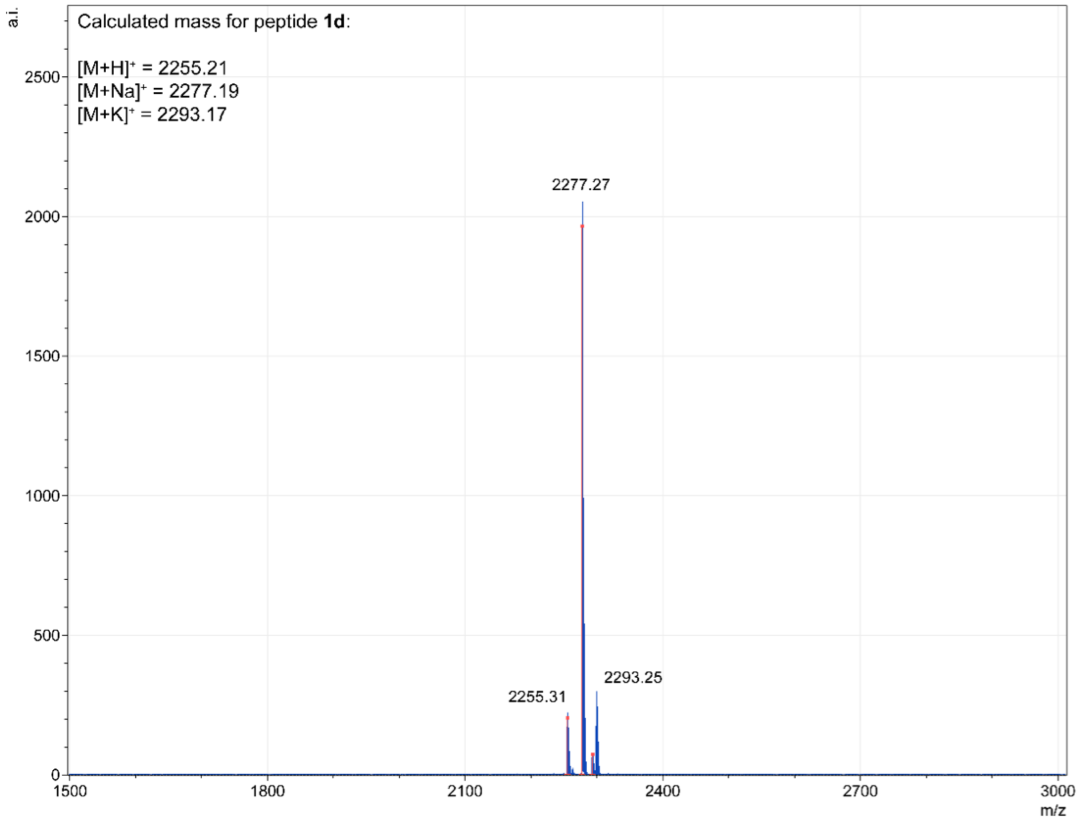
a.i.
Calculated mass for peptide **1d**:
[M+H]⁺ = 2255.21
[M+Na]⁺ = 2277.19
[M+K]⁺ = 2293.17
2277.27
2293.25
2255.31
m/z

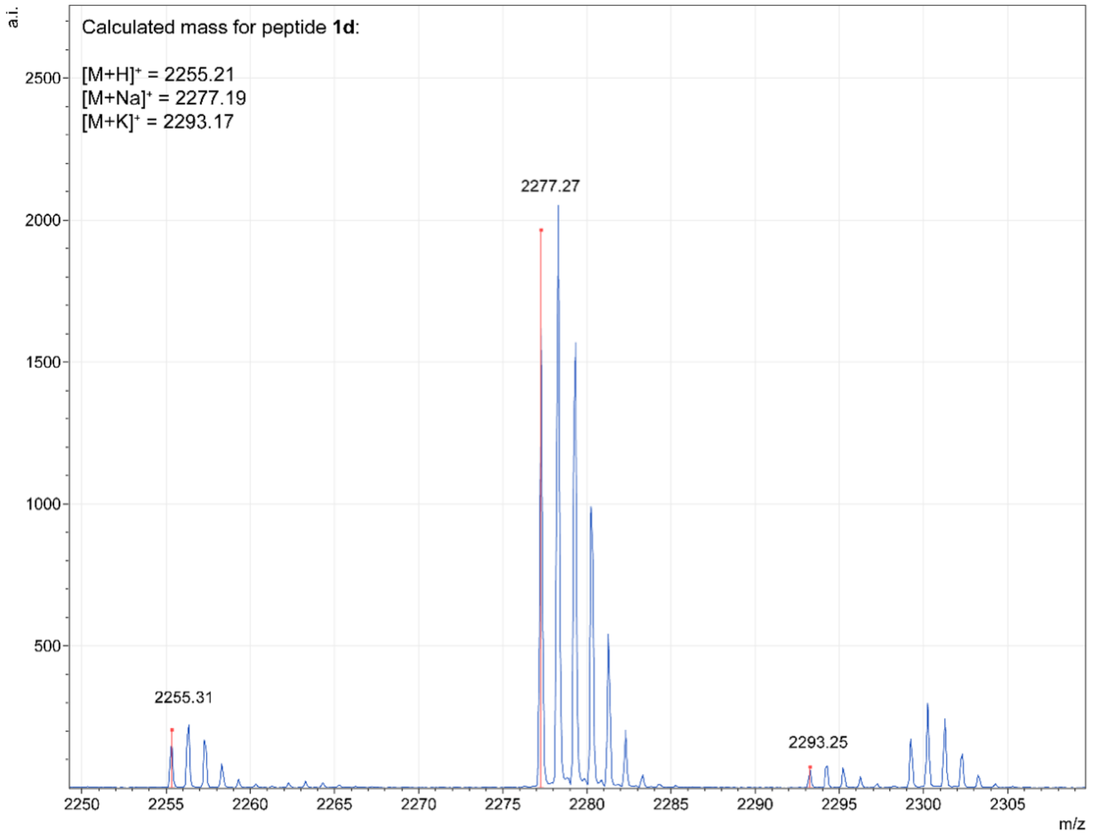
a.i.
Calculated mass for peptide **1d**:
[M+H]⁺ = 2255.21
[M+Na]⁺ = 2277.19
[M+K]⁺ = 2293.17
2277.27
2255.31
2293.25
m/z

*Characterization of peptide **1e***

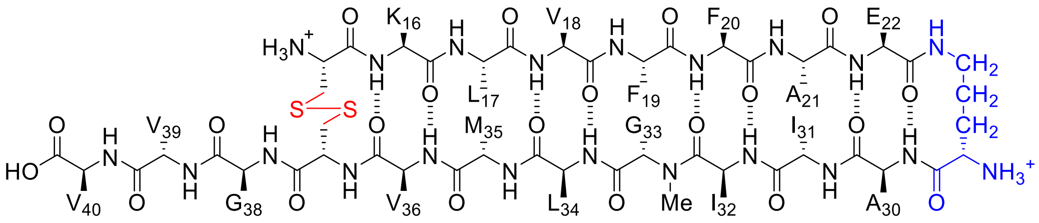

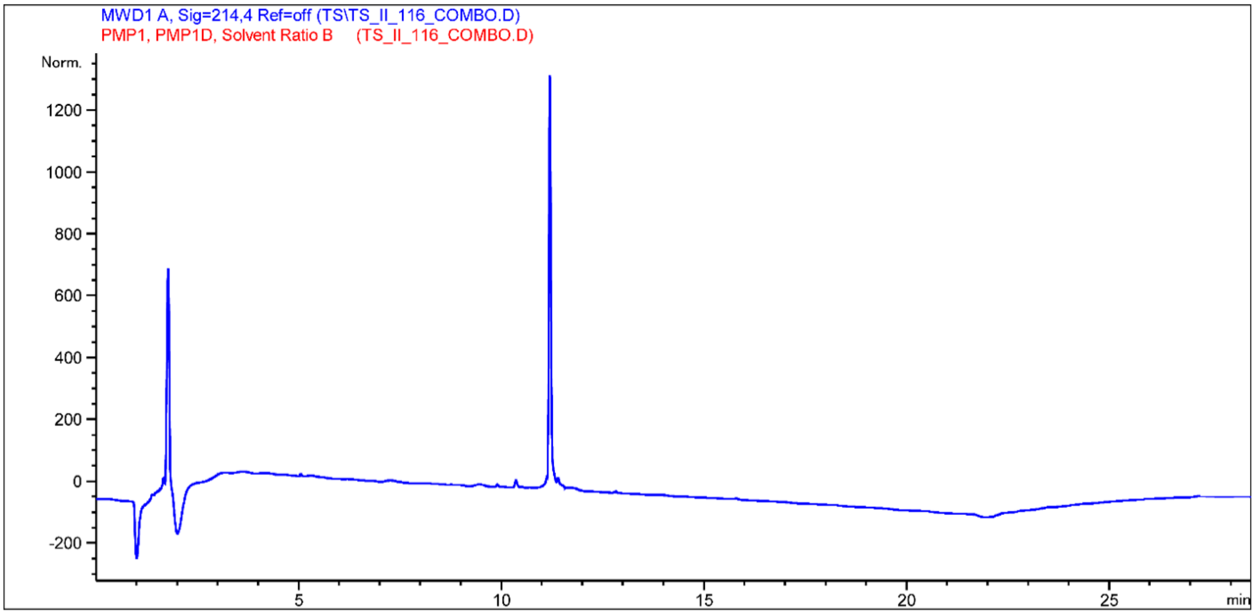

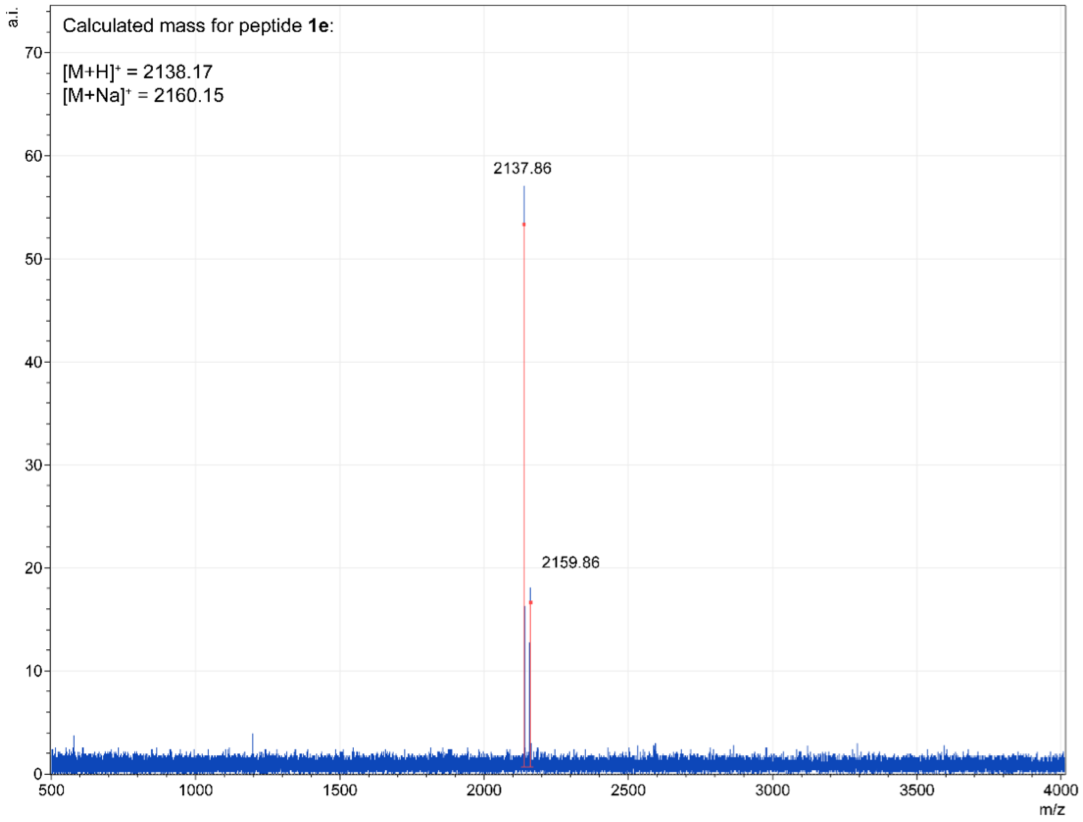
Calculated mass for peptide **1e**:
[M+H]+ = 2138.17
[M+Na]+ = 2160.15
2137.86
2159.86
a.i.
m/z

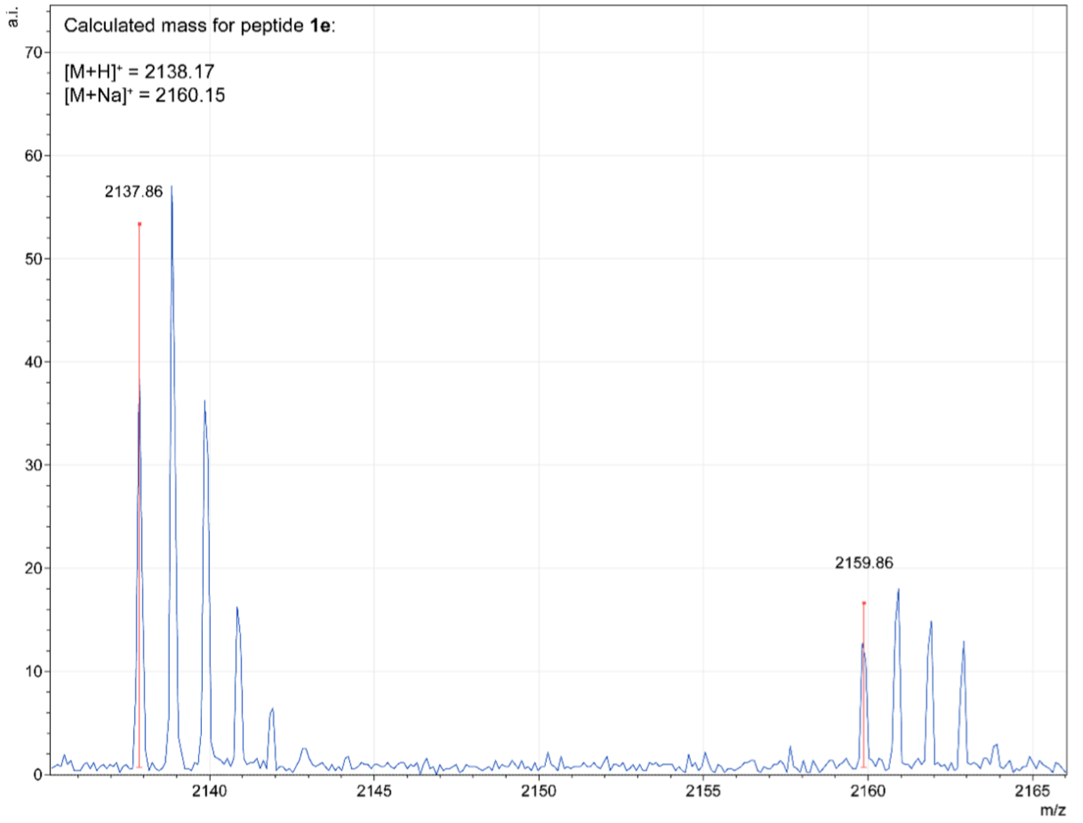
Calculated mass for peptide **1e**:
[M+H]+ = 2138.17
[M+Na]+ = 2160.15
2137.86
2159.86
a.i.
m/z

*Characterization of peptide **1f***

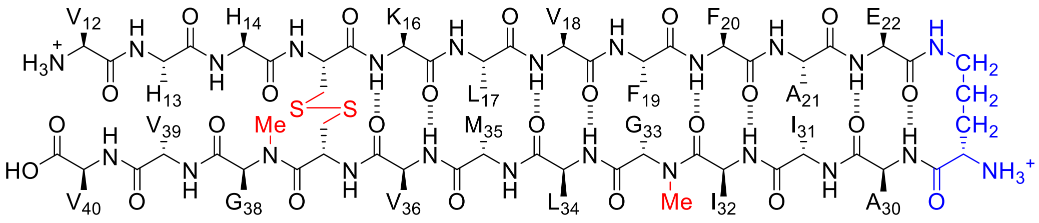

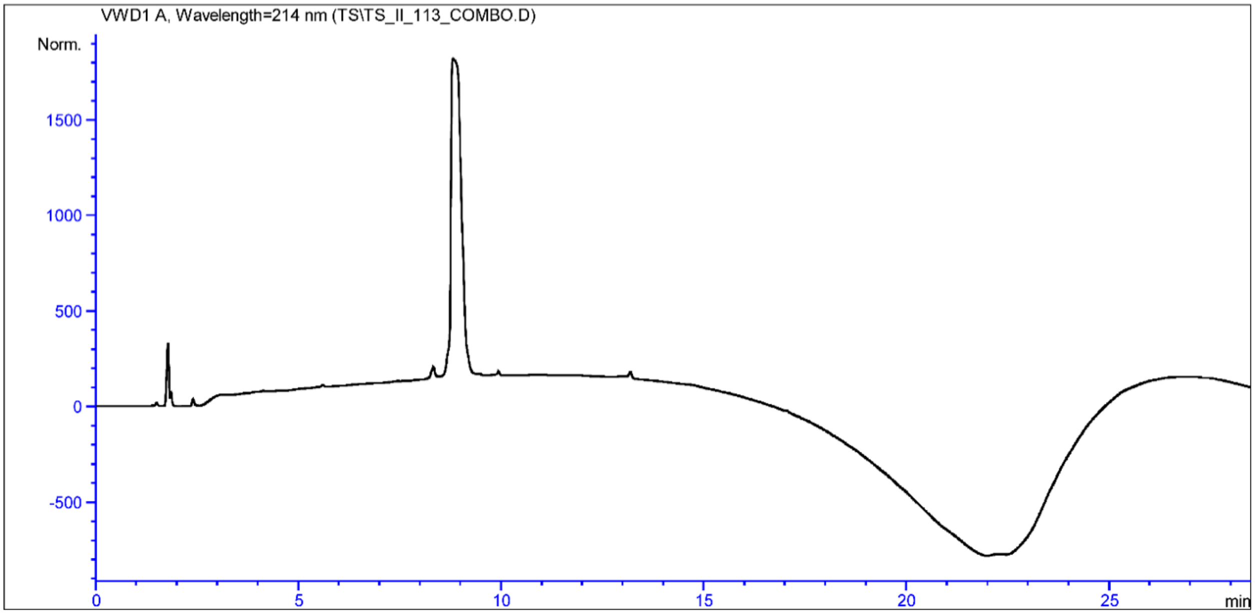

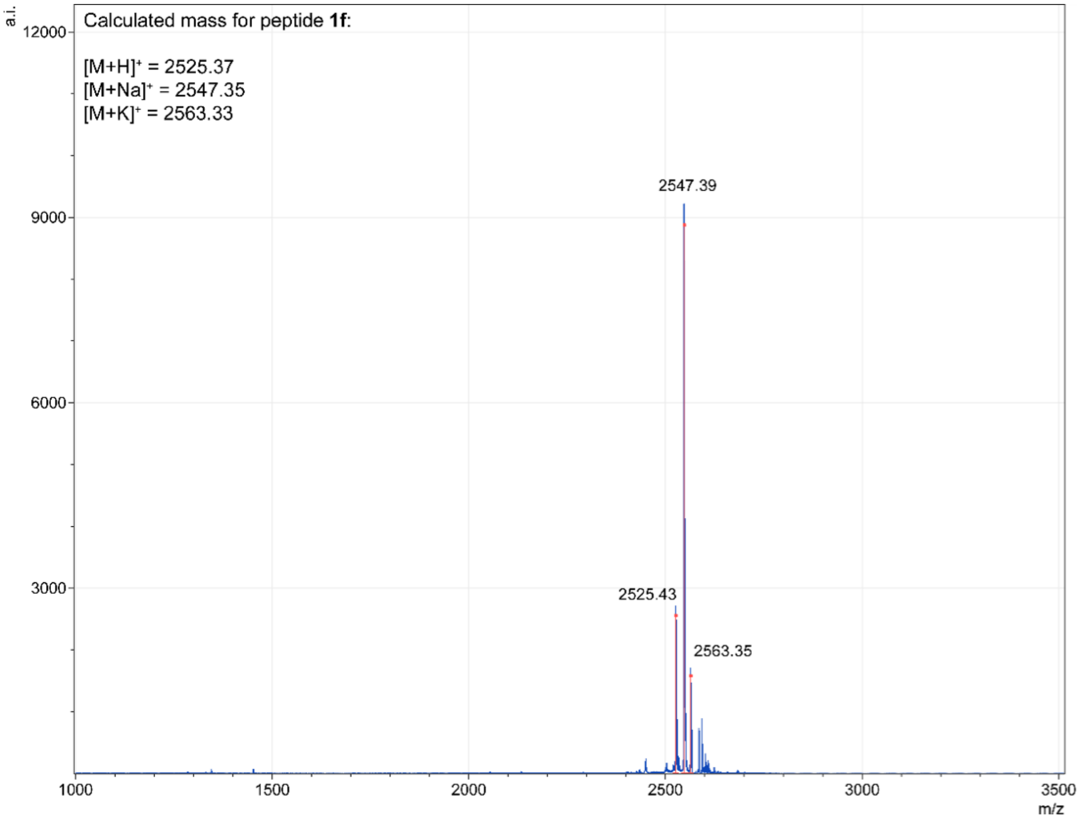
Calculated mass for peptide **1f**:
[M+H]⁺ = 2525.37
[M+Na]⁺ = 2547.35
[M+K]⁺ = 2563.33
2547.39
2525.43
2563.35
a.i.
m/z

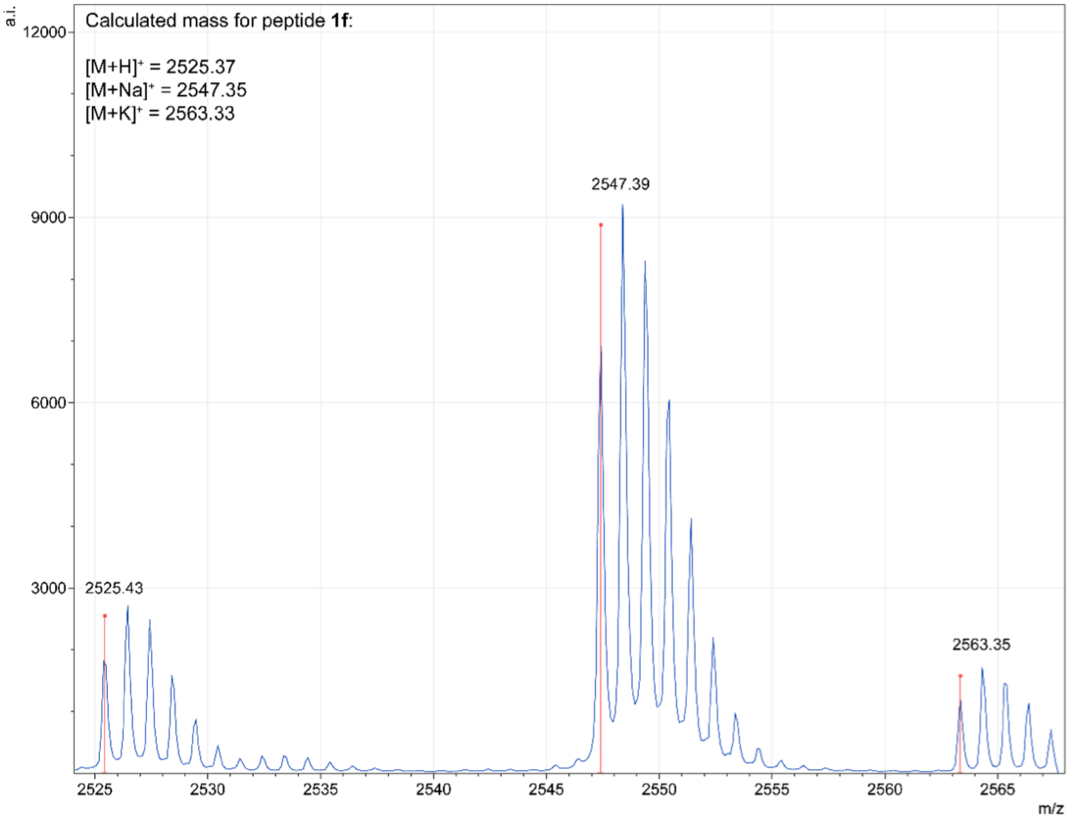
Calculated mass for peptide **1f**:
[M+H]⁺ = 2525.37
[M+Na]⁺ = 2547.35
[M+K]⁺ = 2563.33
2547.39
2525.43
2563.35
a.i.
m/z

*Characterization of peptide **1g***

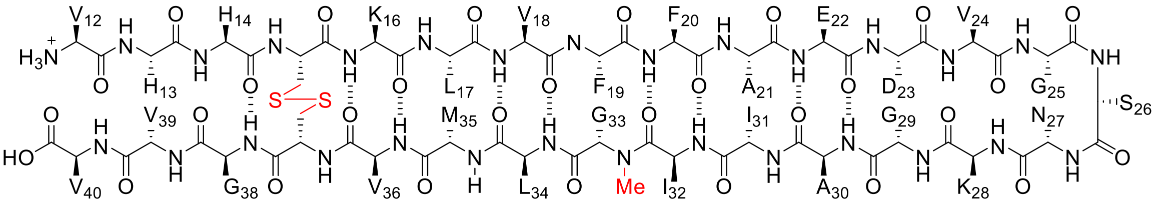

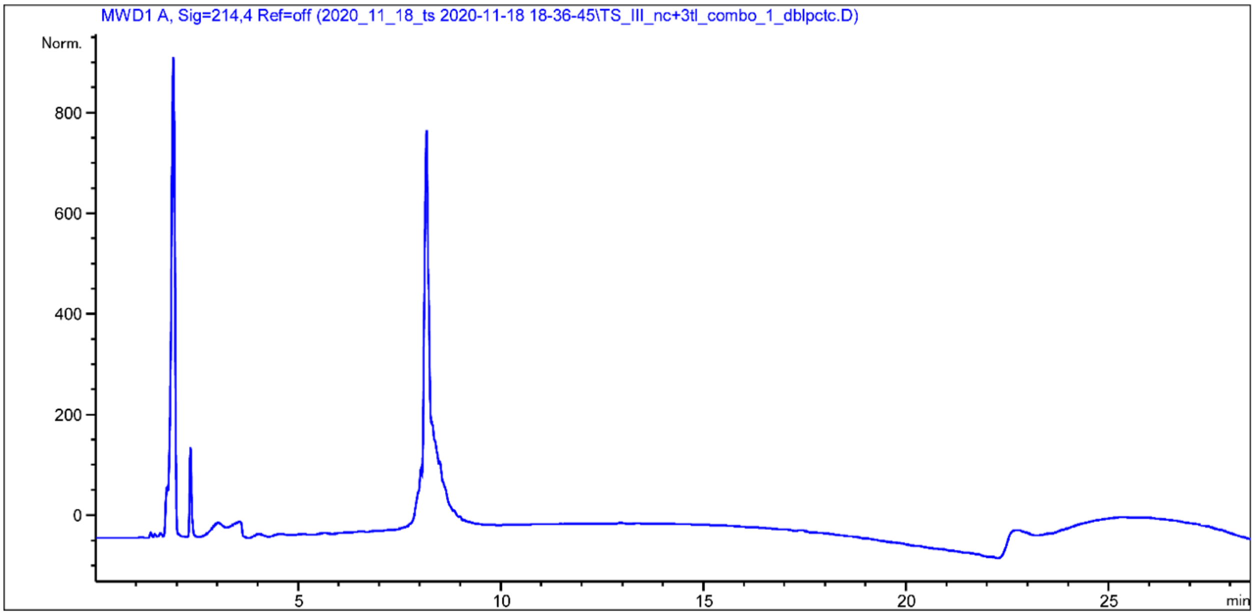

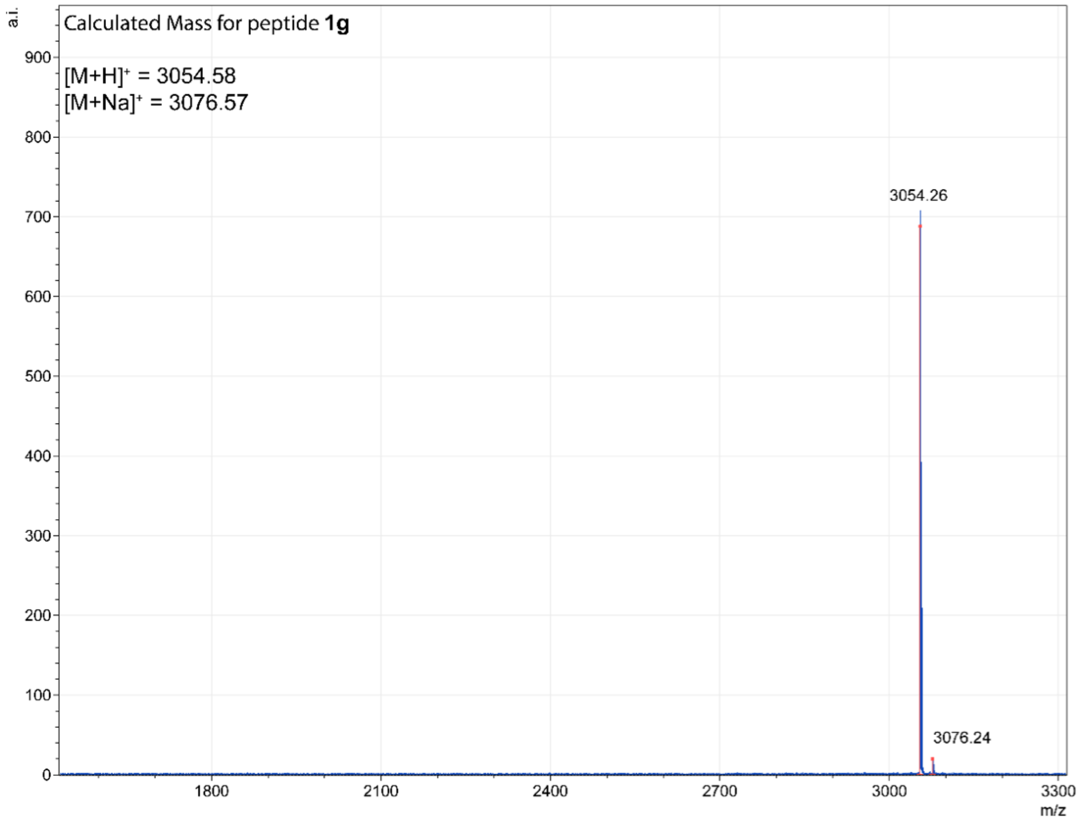
Calculated Mass for peptide **1g**
[M+H]+ = 3054.58
[M+Na]+ = 3076.57
3054.26
3076.24
a.i.
m/z

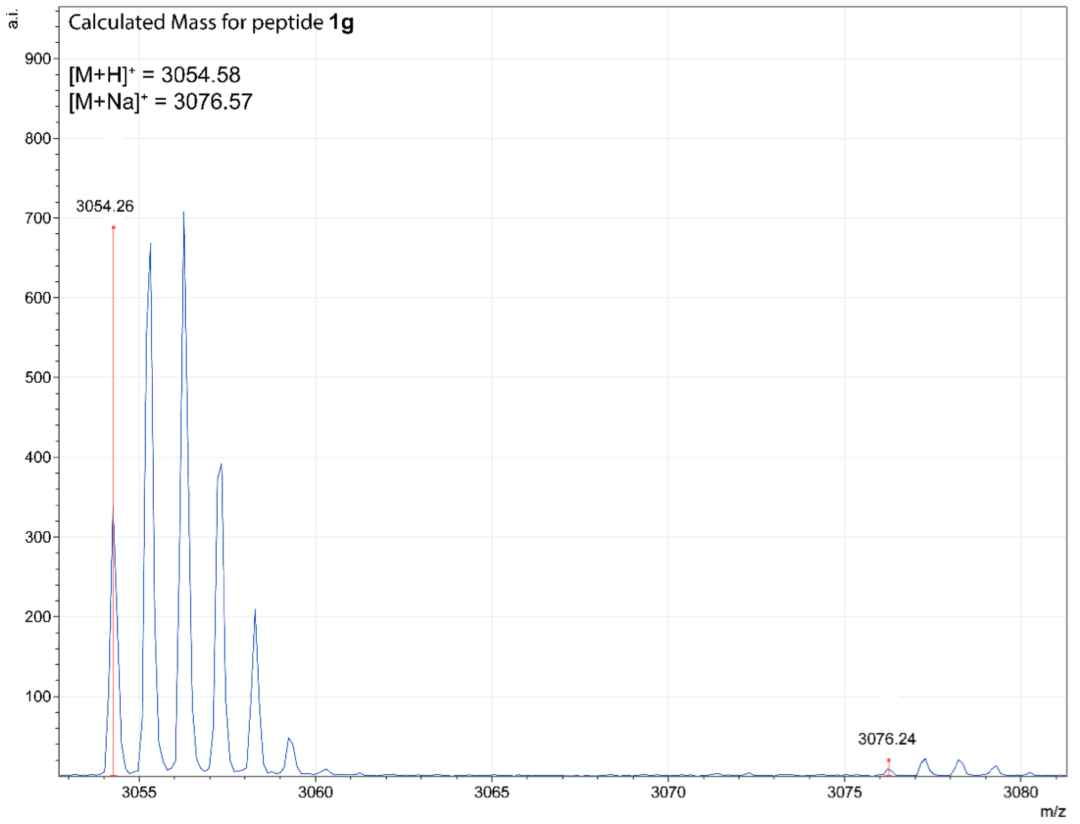
Calculated Mass for peptide **1g**
[M+H]+ = 3054.58
[M+Na]+ = 3076.57
3054.26
3076.24
a.i.
m/z

Characterization of peptide ***1h***

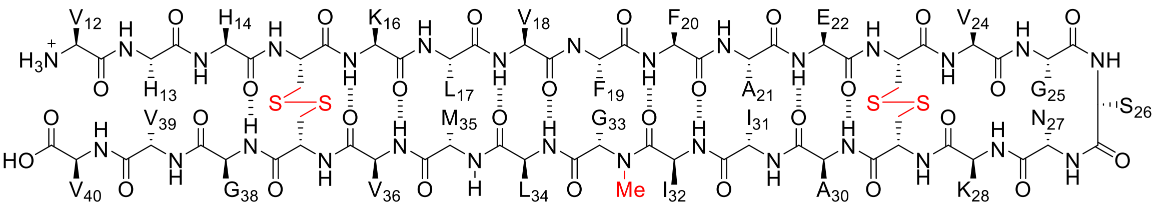

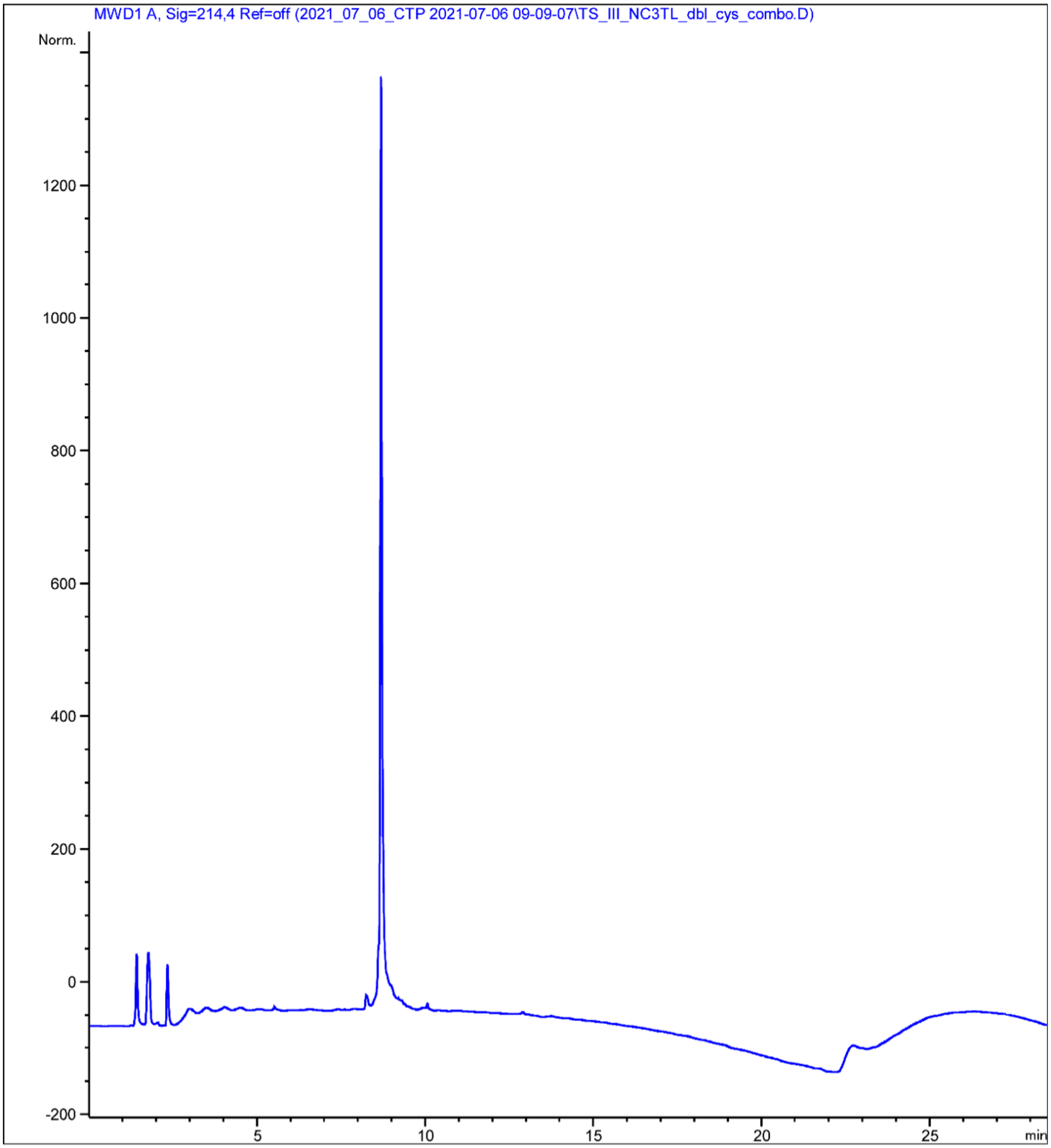

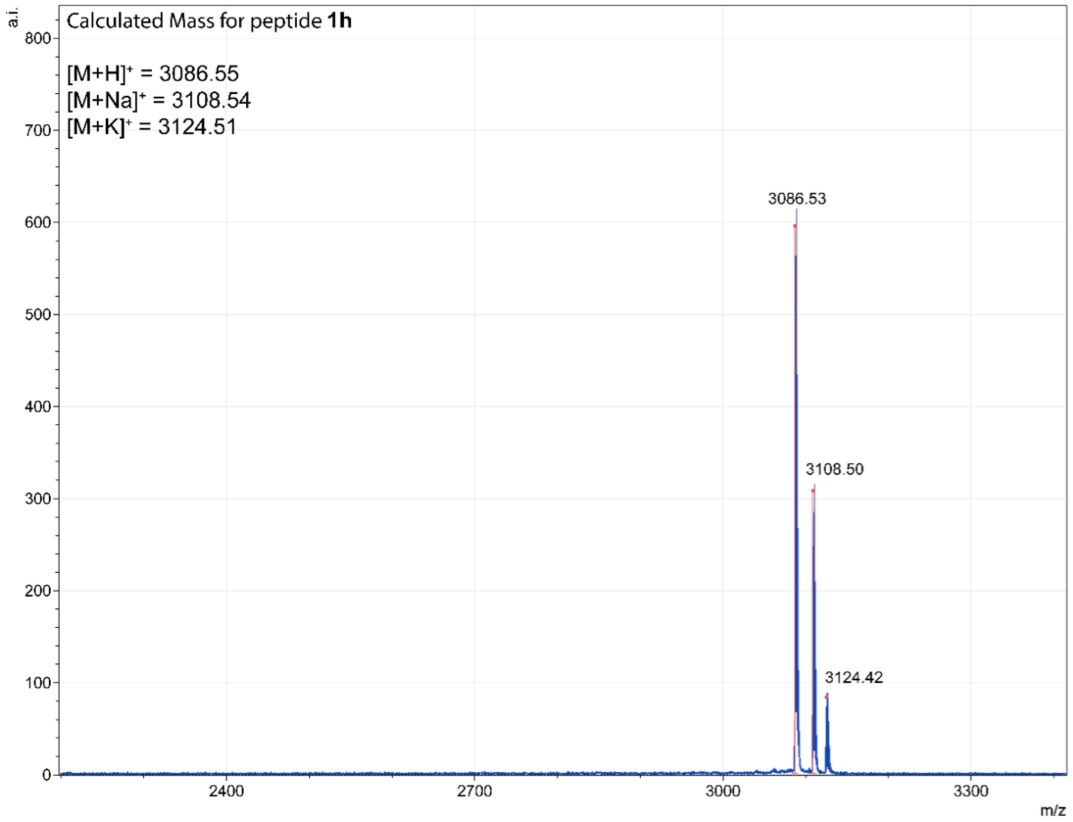

a.i.
Calculated Mass for peptide **1h**
[M+H]+ = 3086.55
[M+Na]+ = 3108.54
[M+K]+ = 3124.51
3086.53
3108.50
3124.42
m/z

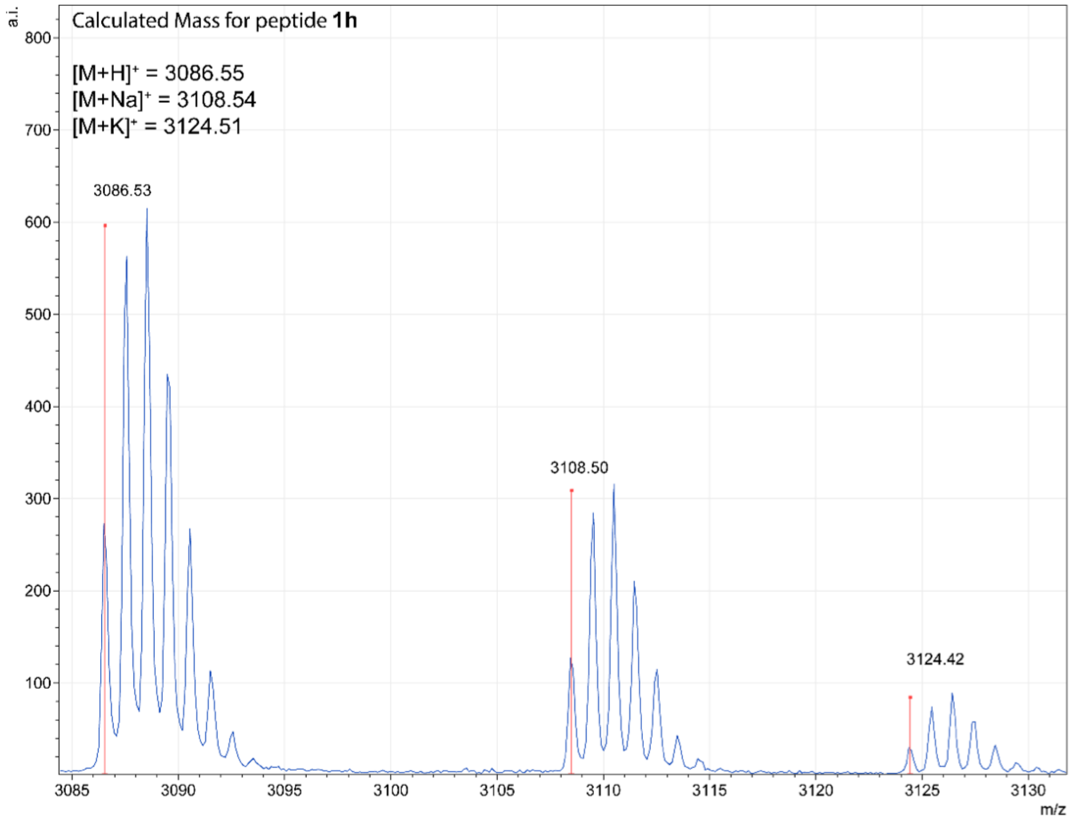

a.i.
Calculated Mass for peptide **1h**
[M+H]+ = 3086.55
[M+Na]+ = 3108.54
[M+K]+ = 3124.51
3086.53
3108.50
3124.42
m/z

*Characterization of peptide **1i***

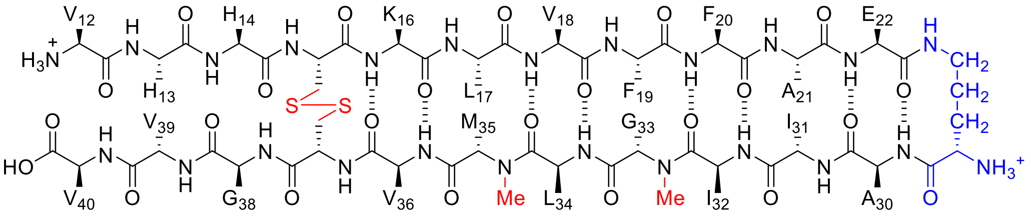

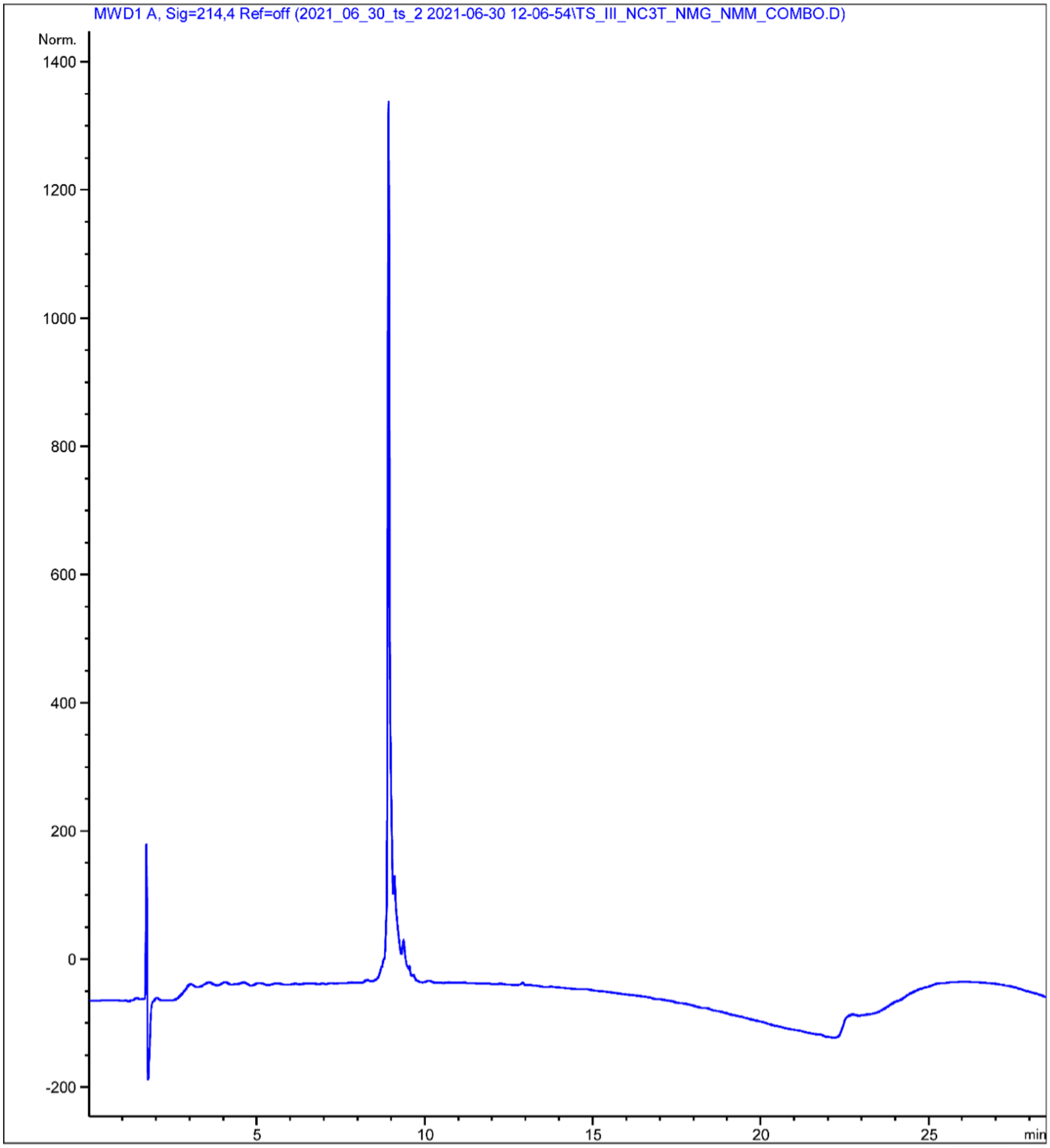

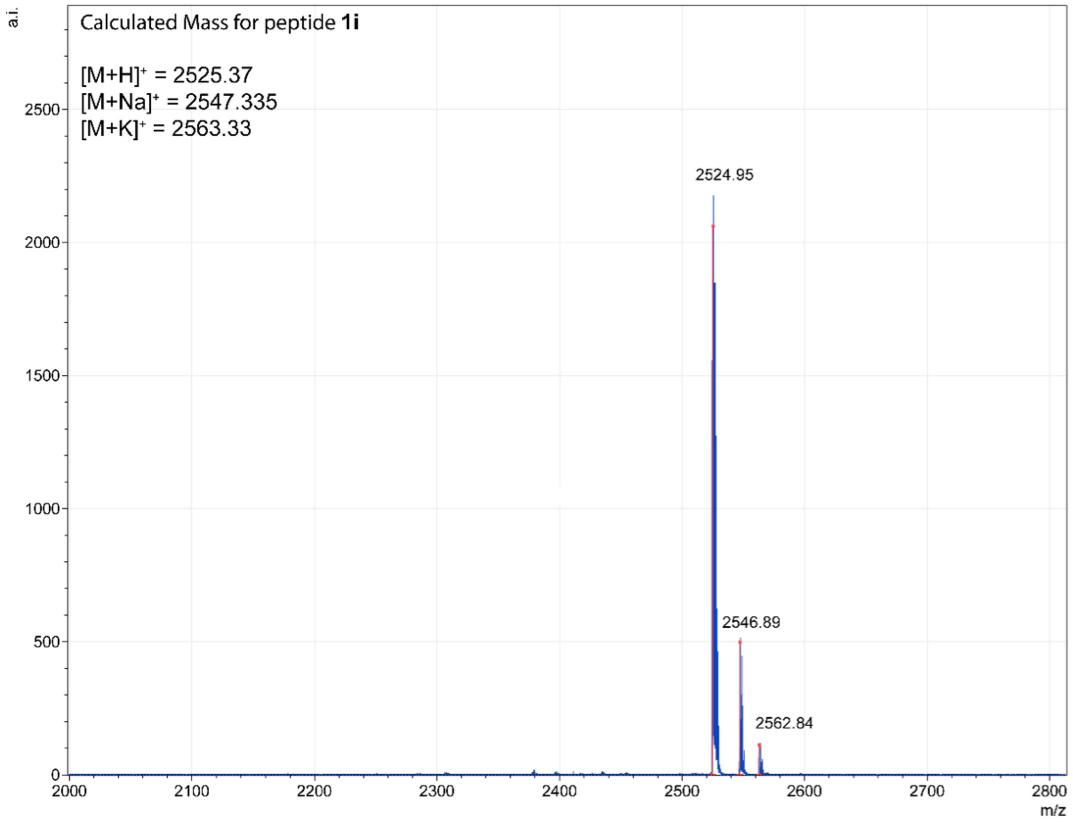

a.i.
Calculated Mass for peptide **1i**
[M+H]+ = 2525.37
[M+Na]+ = 2547.335
[M+K]+ = 2563.33
2524.95
2546.89
2562.84
m/z

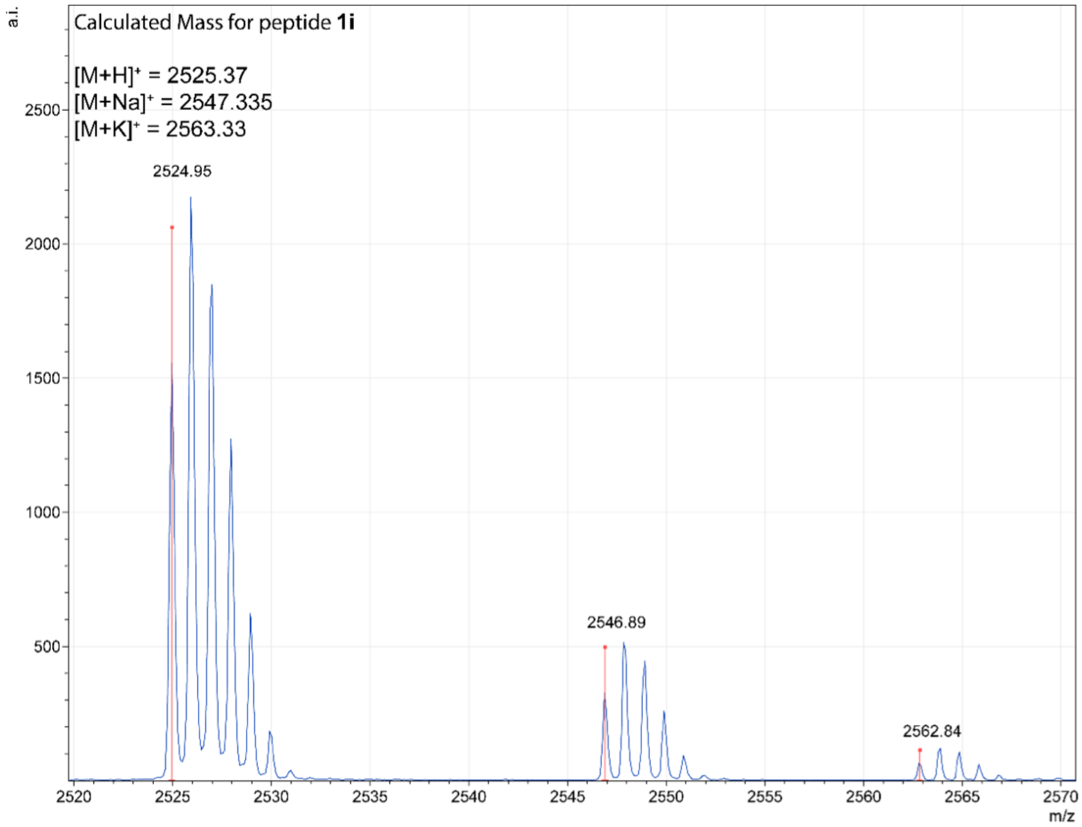

a.i.
Calculated Mass for peptide **1i**
[M+H]+ = 2525.37
[M+Na]+ = 2547.335
[M+K]+ = 2563.33
2524.95
2546.89
2562.84
m/z

Characterization of peptide ***2a***

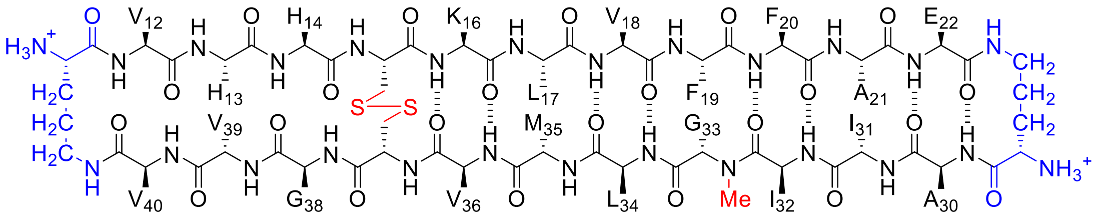

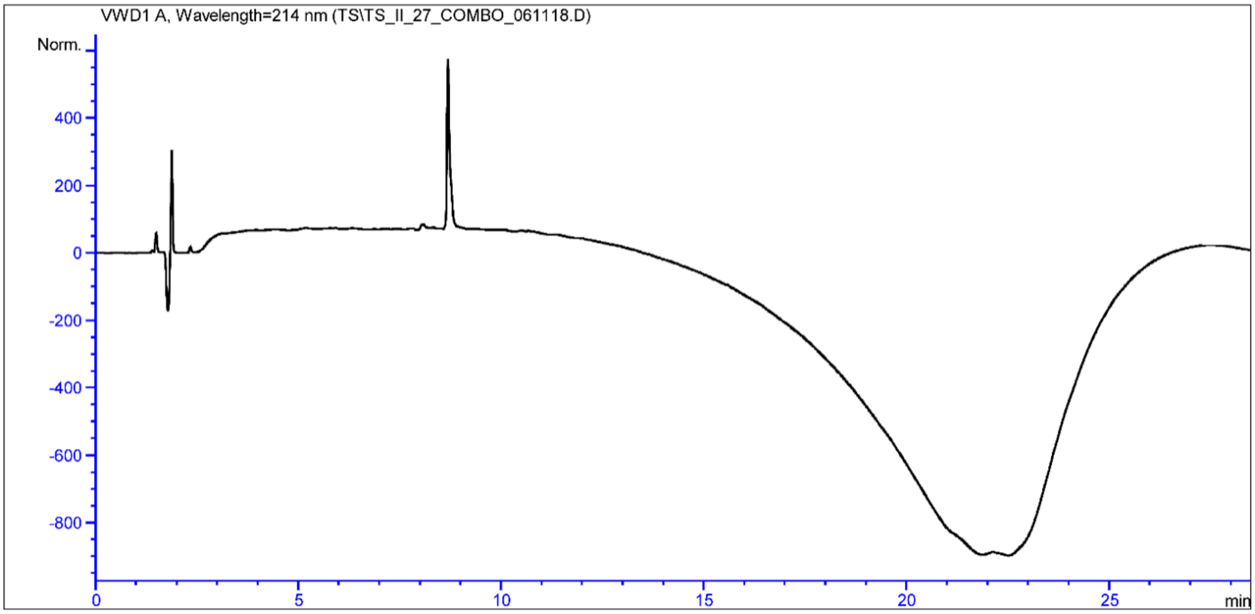

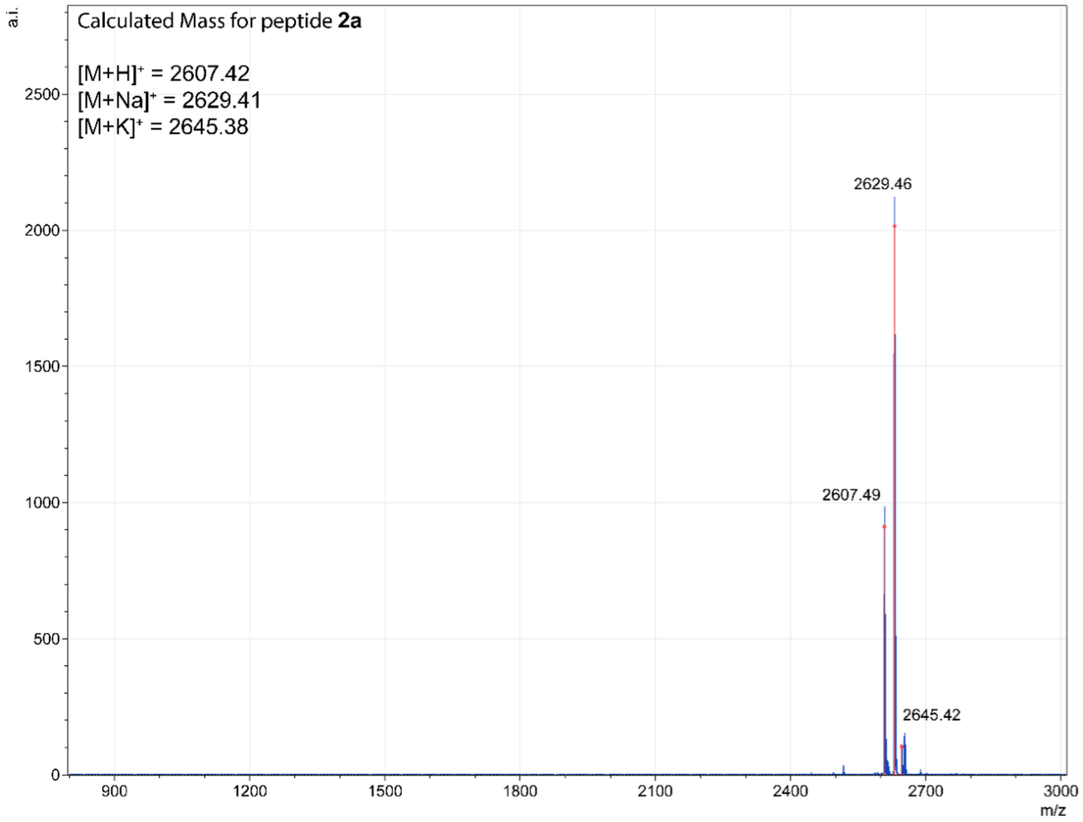
Calculated Mass for peptide 2a
[M+H]+ = 2607.42
[M+Na]+ = 2629.41
[M+K]+ = 2645.38
2629.46
2607.49
2645.42
a.i.
m/z

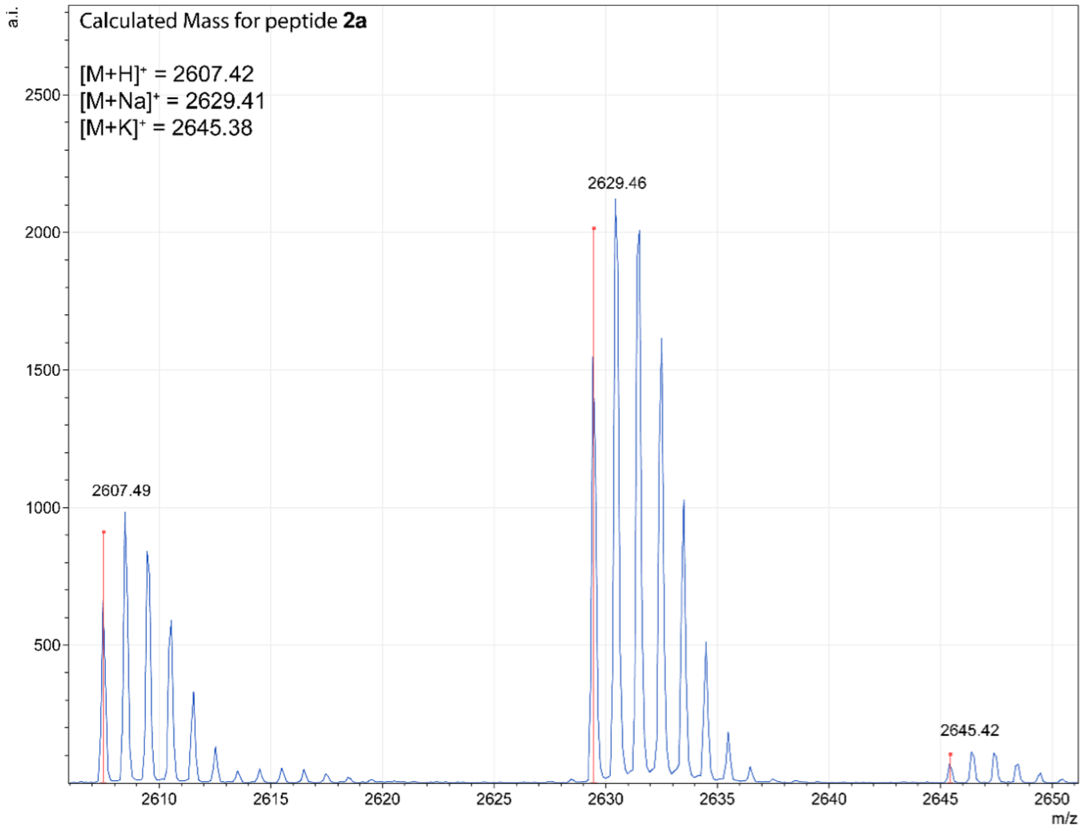
Calculated Mass for peptide 2a
[M+H]+ = 2607.42
[M+Na]+ = 2629.41
[M+K]+ = 2645.38
2629.46
2607.49
2645.42
a.i.
m/z

*Characterization of peptide **2b***

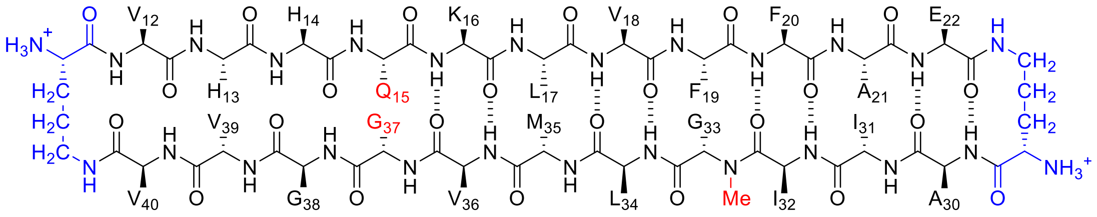

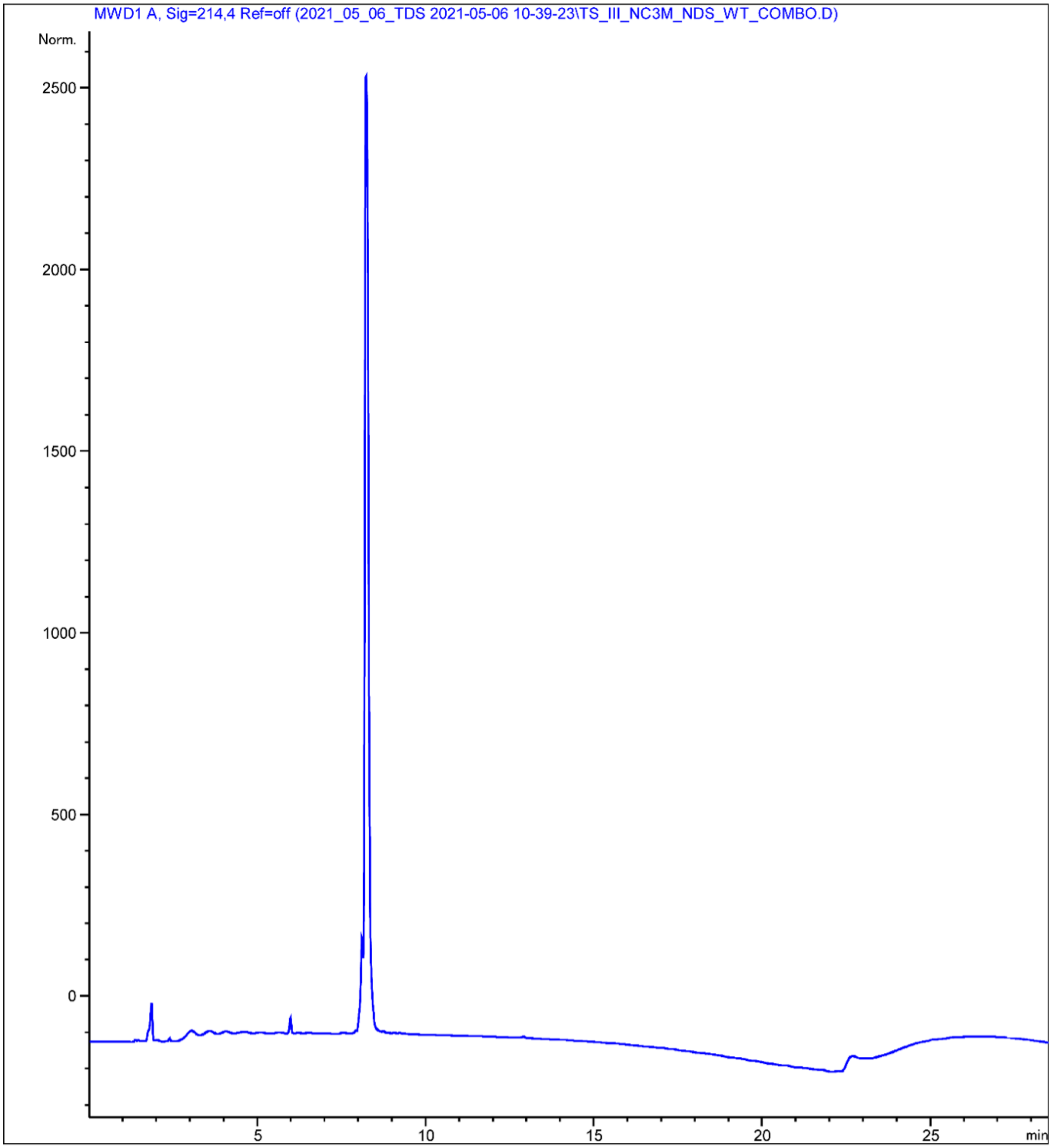

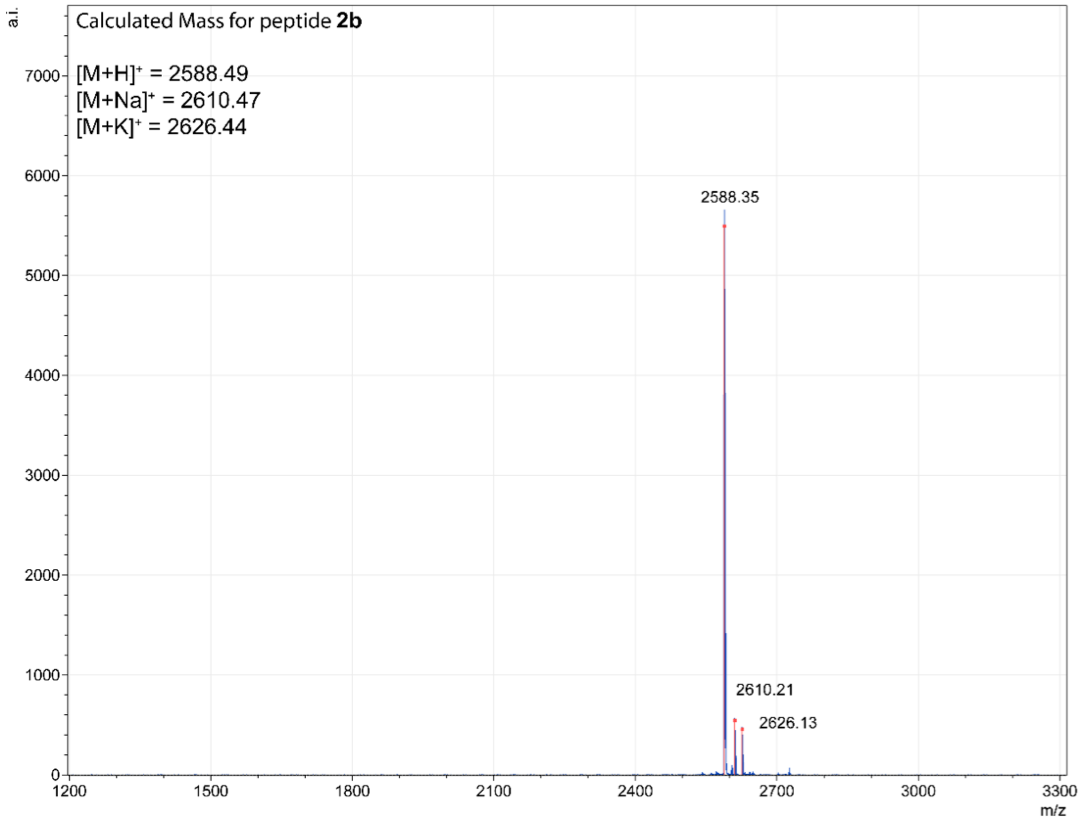
Calculated Mass for peptide 2b
[M+H]+ = 2588.49
[M+Na]+ = 2610.47
[M+K]+ = 2626.44
2588.35
2610.21
2626.13
a.i.
m/z

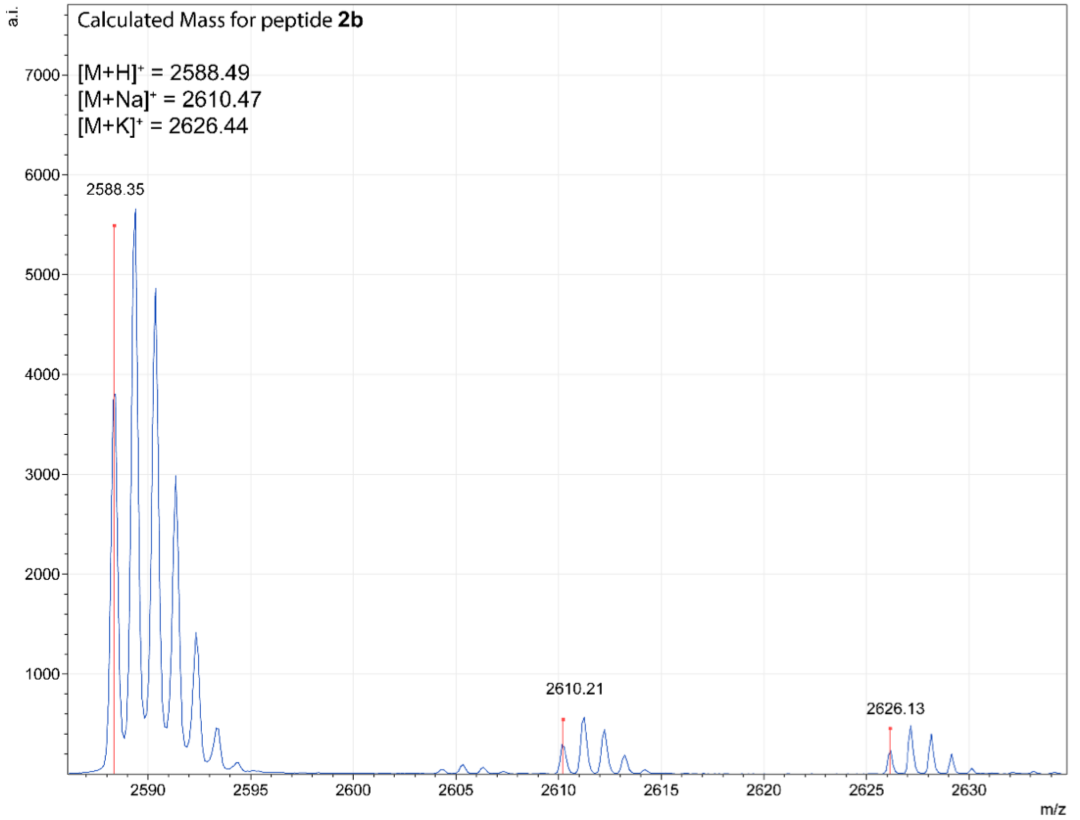
Calculated Mass for peptide 2b
[M+H]+ = 2588.49
[M+Na]+ = 2610.47
[M+K]+ = 2626.44
2588.35
2610.21
2626.13
a.i.
m/z

*Characterization of peptide **2c***

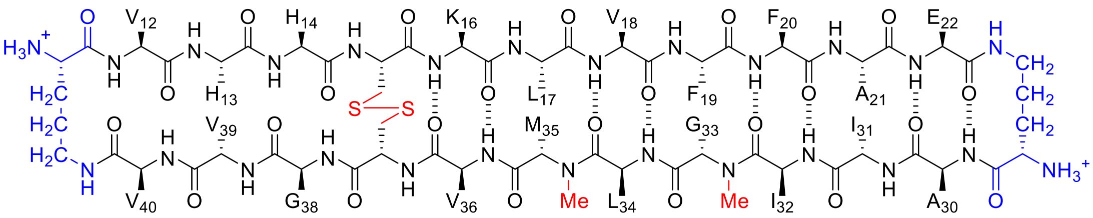

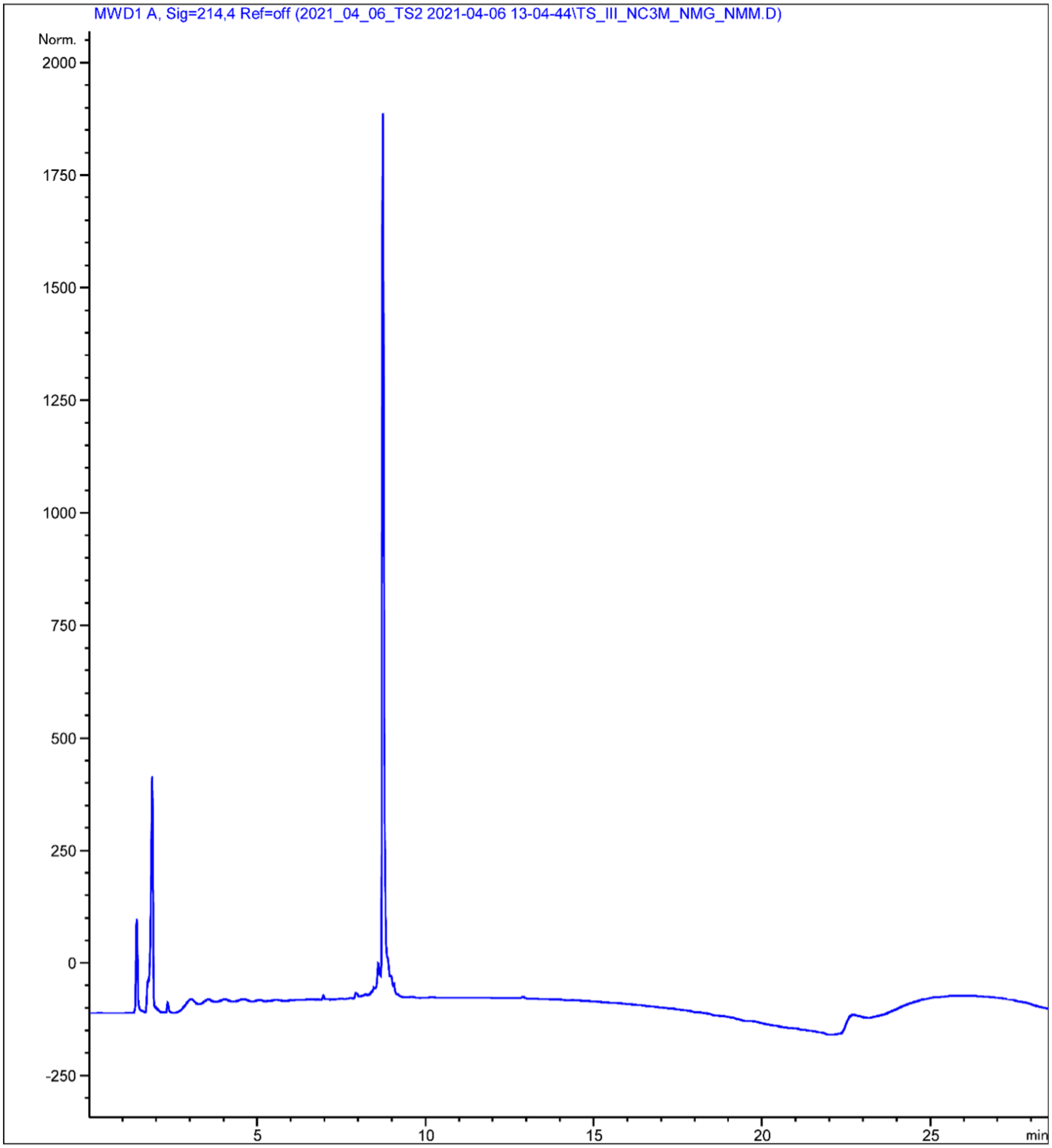

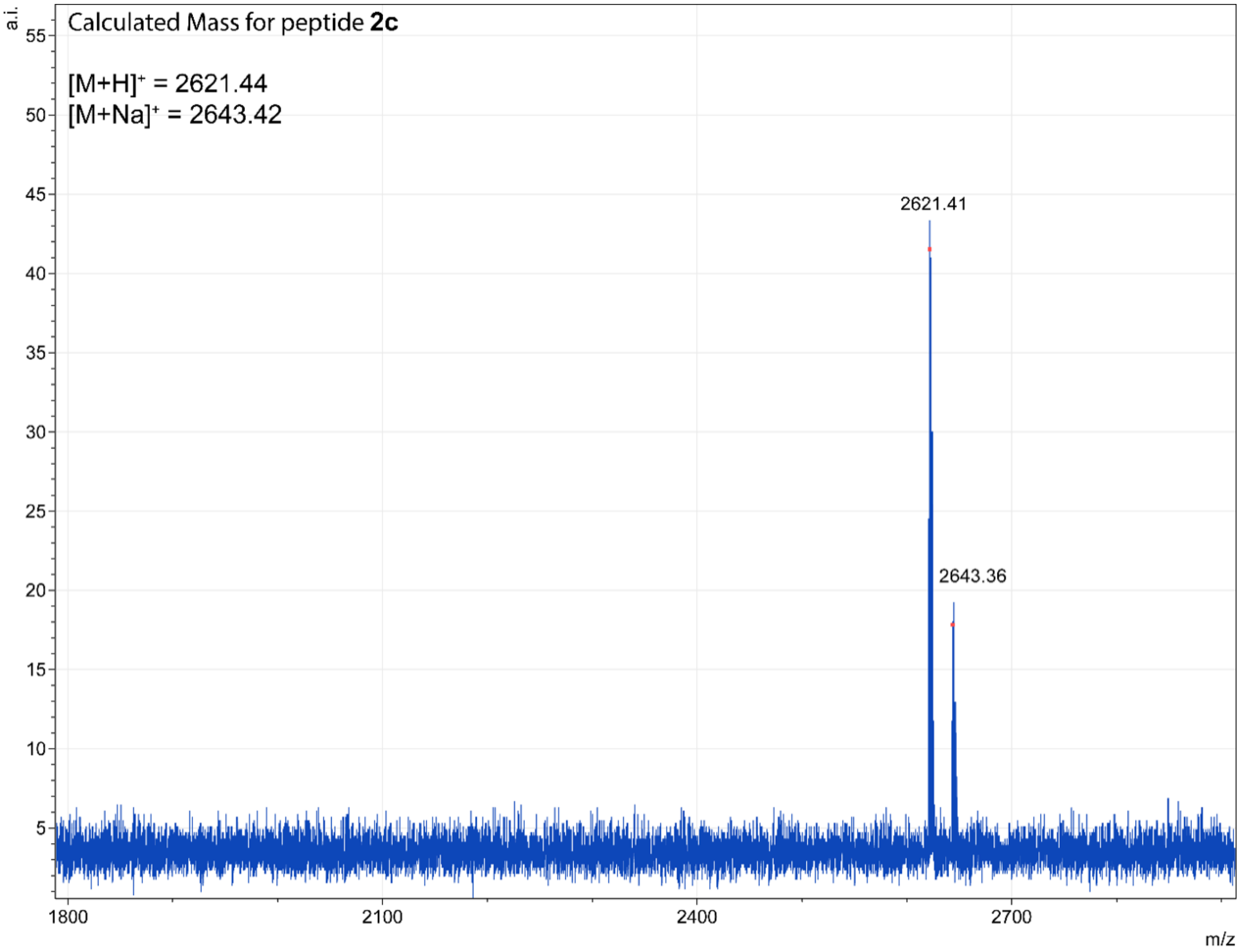
Calculated Mass for peptide **2c**
[M+H]+ = 2621.44
[M+Na]+ = 2643.42
2621.41
2643.36
a.i.
m/z

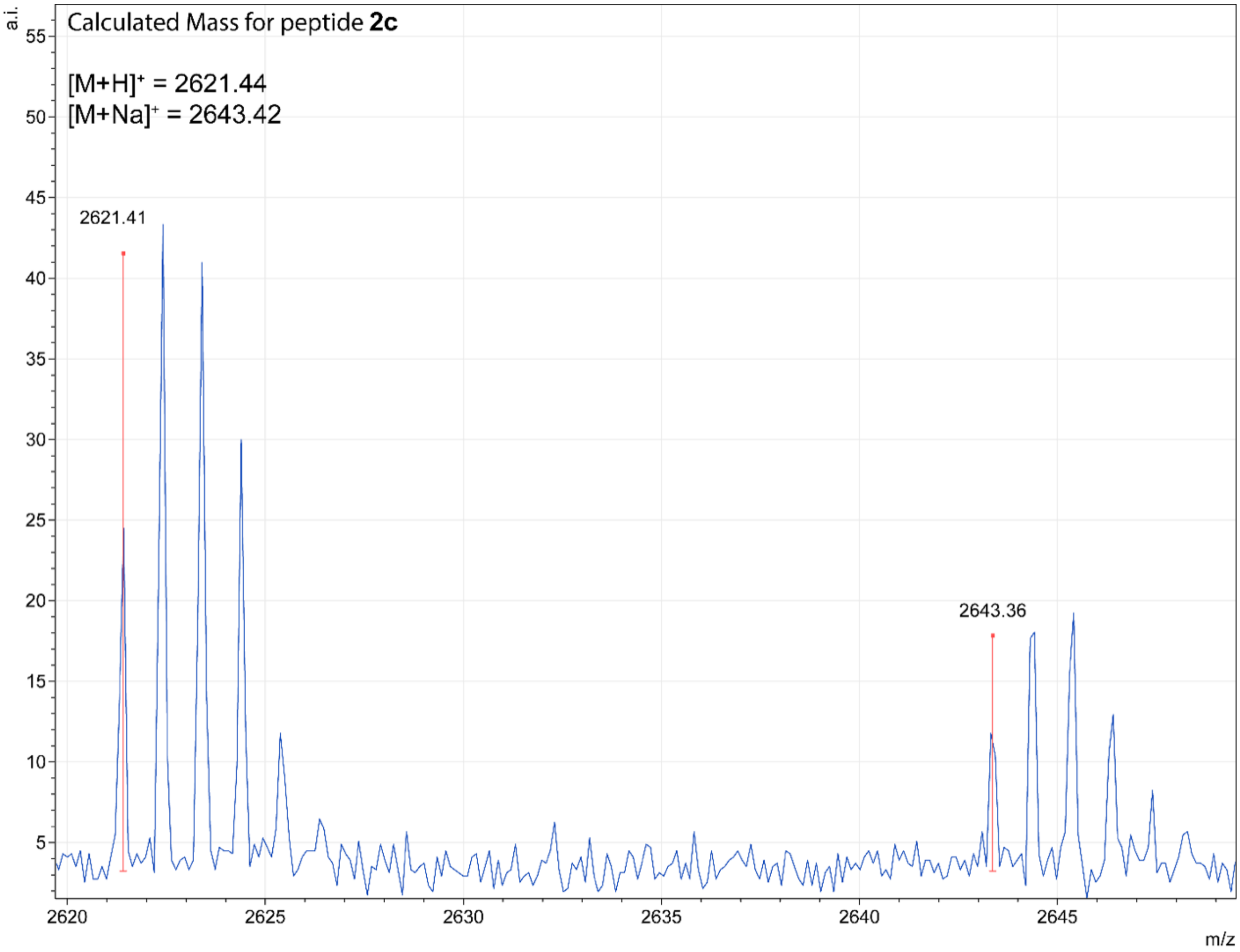
Calculated Mass for peptide **2c**
[M+H]+ = 2621.44
[M+Na]+ = 2643.42
2621.41
2643.36
a.i.
55
50
45
40
35
30
25
20
15
10
5
2620
2625
2630
2635
2640
2645
m/z

*Characterization of peptide **2d***

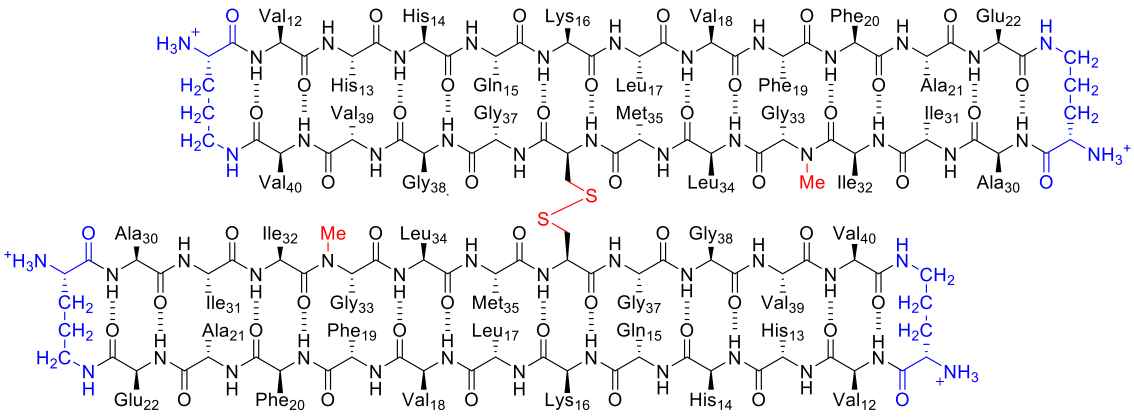

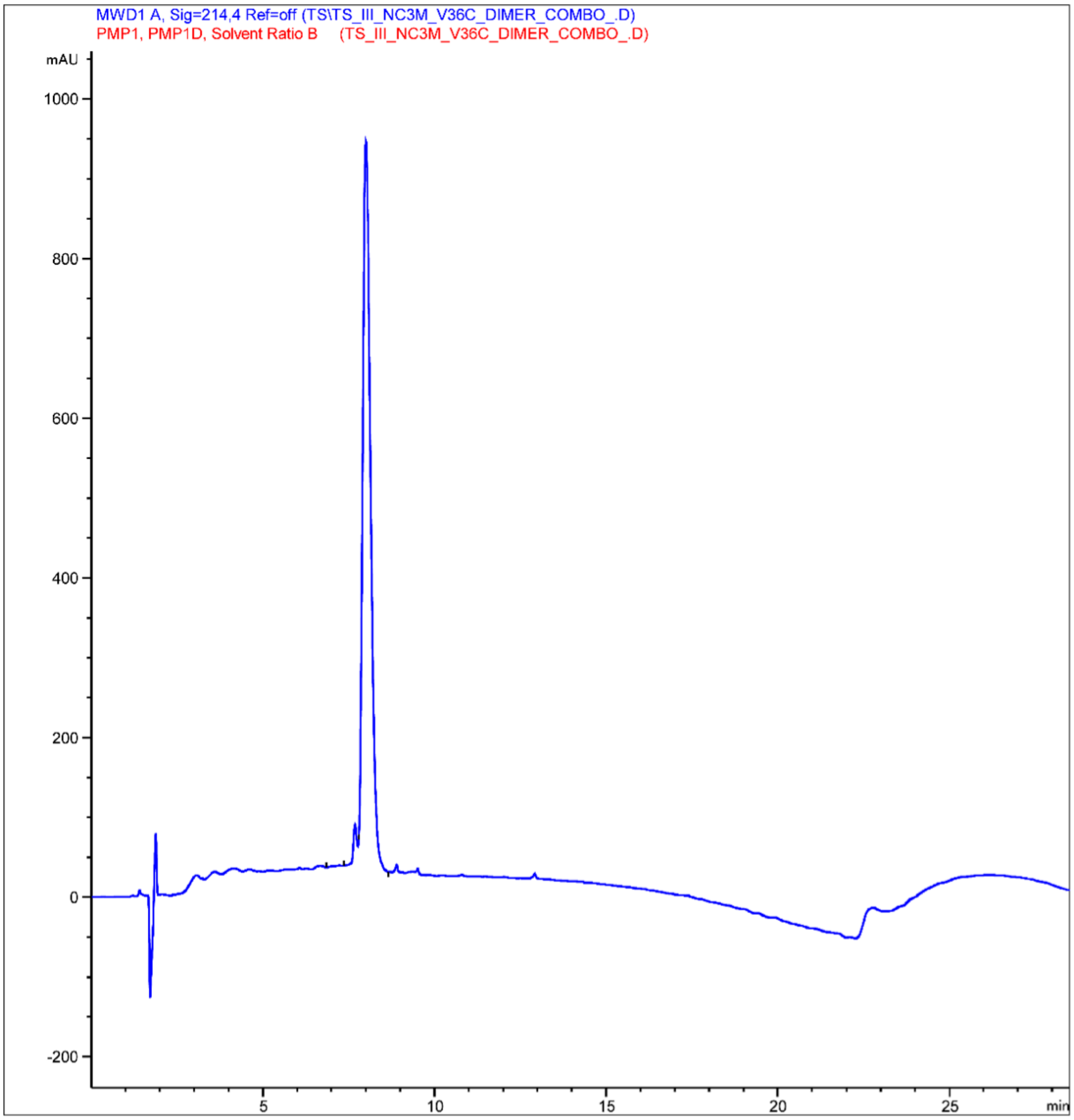

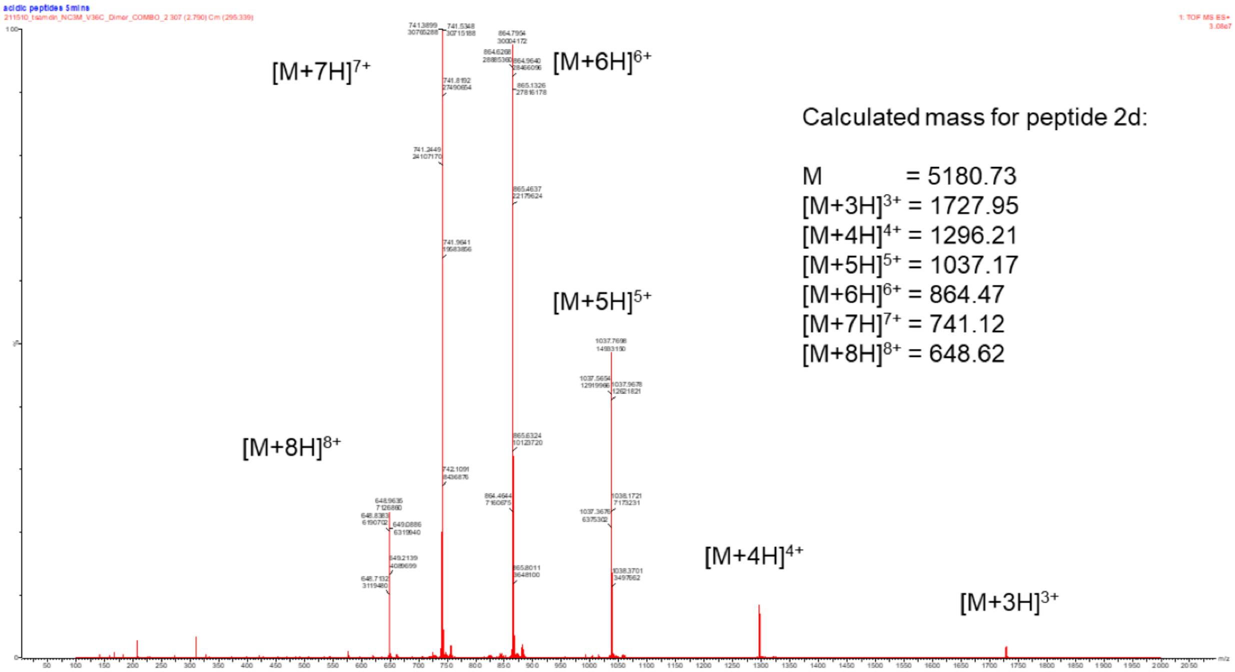
[M+7H]7+
[M+6H]6+
[M+5H]5+
[M+8H]8+
[M+4H]4+
[M+3H]3+
Calculated mass for peptide 2d:
M = 5180.73
[M+3H]3+ = 1727.95
[M+4H]4+ = 1296.21
[M+5H]5+ = 1037.17
[M+6H]6+ = 864.47
[M+7H]7+ = 741.12
[M+8H]8+ = 648.62

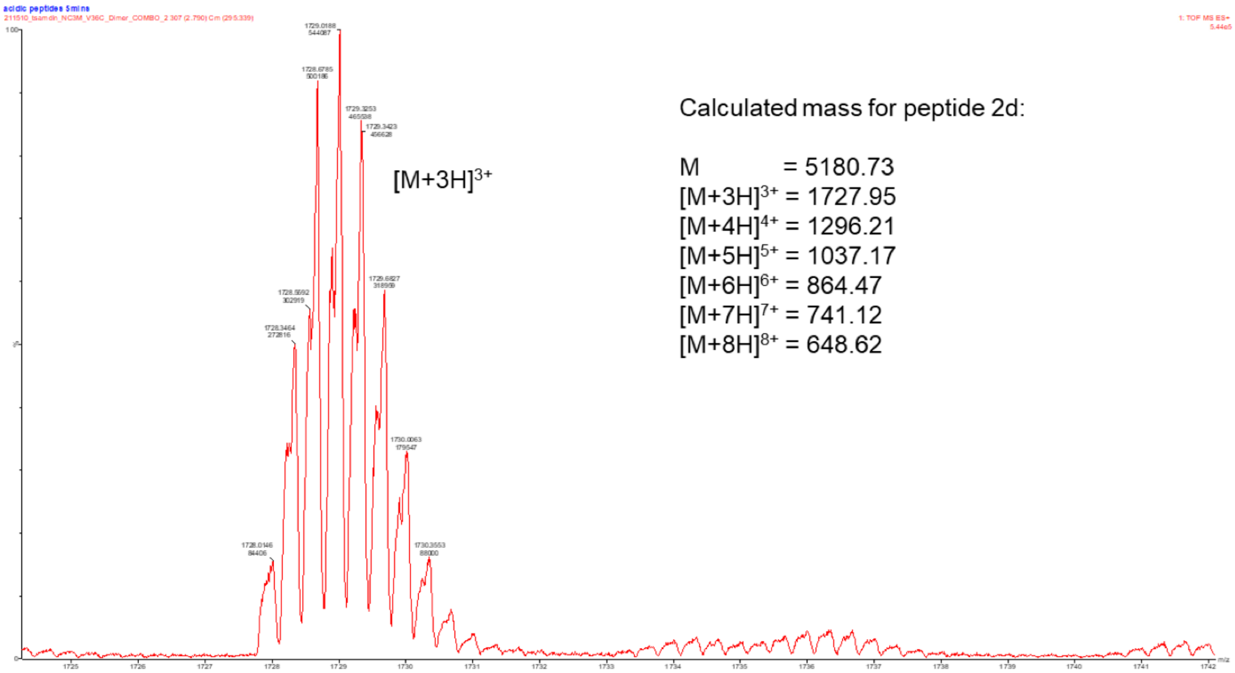
[M+3H]3+
Calculated mass for peptide 2d:
M = 5180.73
[M+3H]3+ = 1727.95
[M+4H]4+ = 1296.21
[M+5H]5+ = 1037.17
[M+6H]6+ = 864.47
[M+7H]7+ = 741.12
[M+8H]8+ = 648.62

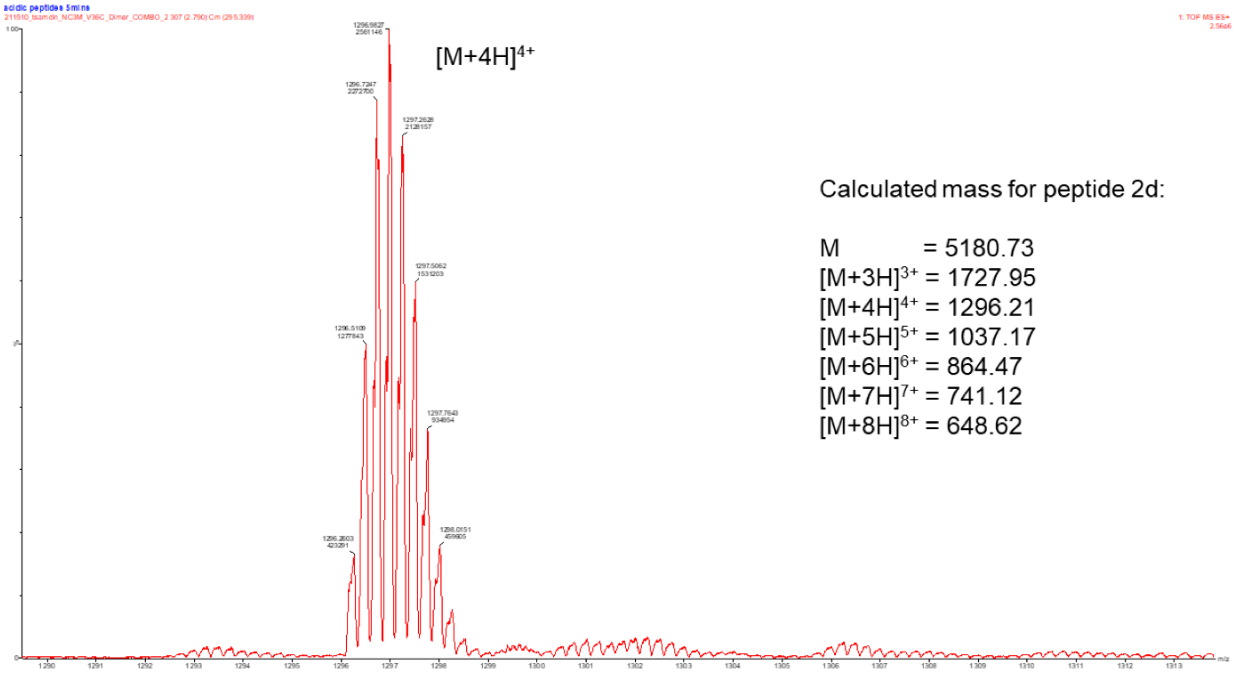
[M+4H]4+
Calculated mass for peptide 2d:
M = 5180.73
[M+3H]3+ = 1727.95
[M+4H]4+ = 1296.21
[M+5H]5+ = 1037.17
[M+6H]6+ = 864.47
[M+7H]7+ = 741.12
[M+8H]8+ = 648.62

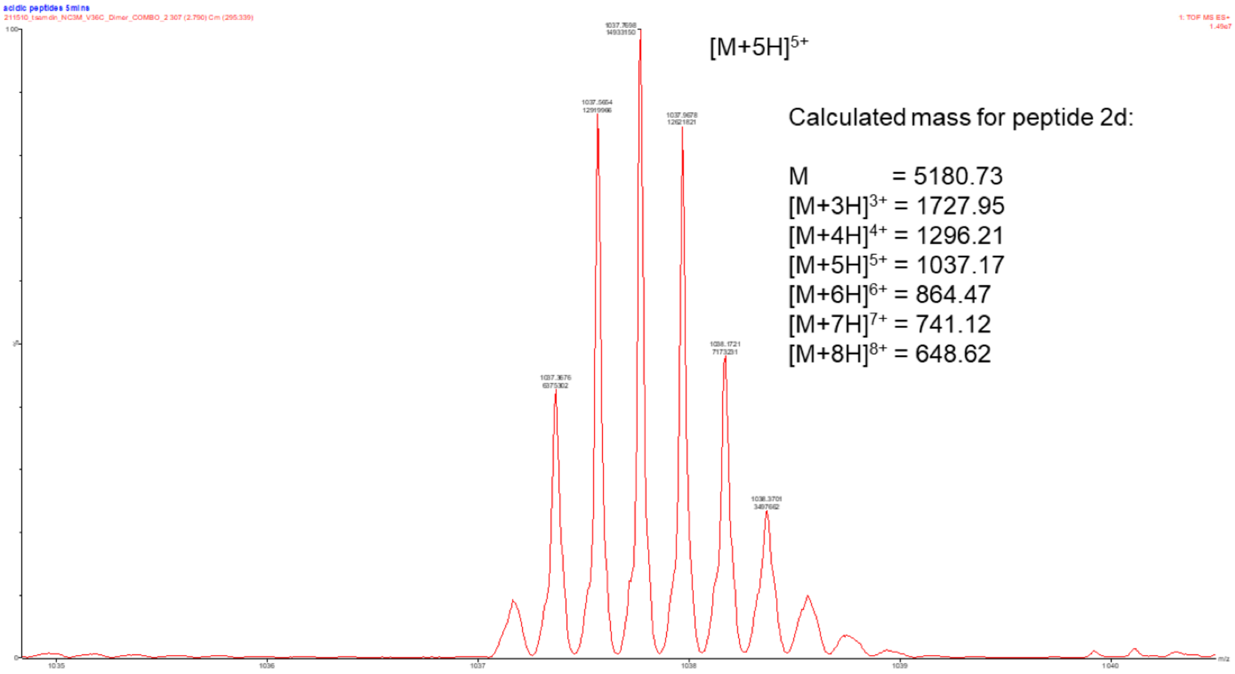
[M+5H]5+
Calculated mass for peptide 2d:
M = 5180.73
[M+3H]3+ = 1727.95
[M+4H]4+ = 1296.21
[M+5H]5+ = 1037.17
[M+6H]6+ = 864.47
[M+7H]7+ = 741.12
[M+8H]8+ = 648.62
